# JAK2V617F reprograms Hypoxia Inducible Factor-1 to induce a non-canonical hypoxia regulon in myeloproliferative neoplasms

**DOI:** 10.1038/s41375-025-02843-9

**Published:** 2026-02-02

**Authors:** David Kealy, Ruth Ellerington, Suraj Bansal, Jessie J. F. Medeiros, Catherine A. Hawley, Andy G. X. Zeng, Jakub Lukaszonek, Katie A. West, Aparna D. Sinha, Gillian Caalim, Richard T. Gawne, Jacob Pope, Bianca Lima Ferreira, Nicole-Mae Blacknell, Bryce Drylie, Jenny Chatzigerou, Hwei Minn Khoo, Adam C. Wilkinson, Adele K. Fielding, Guanlin Wang, Bethan Psaila, David G. Kent, Ian S. Hitchcock, Andrew N. Holding, Andrew S. Mason, Vikas Gupta, John E. Dick, Katherine S. Bridge

**Affiliations:** 1 Centre for Blood Research, University of York, York, UK; 2 York Biomedical Research Institute, University of York, York, UK; 3 Princess Margaret Cancer Centre, University Health Network, Toronto, ON Canada; 4 University of Toronto, Toronto, ON Canada; 5 Jack Birch Unit for Molecular Carcinogenesis, University of York, York, UK; 6 Barts Cancer Institute, Queen Mary University of London, London, UK; 7 Medical Research Council Weatherall Institute of Molecular Medicine (MRC WIMM) and NIHR Biomedical Research Centre Haematology Theme, University of Oxford, Oxford, UK; 8 University of Cambridge, Cambridge, UK; 9 MRC WIMM Centre for Computational Biology, University of Oxford, Oxford, UK; 10 Shanghai Key Laboratory of Metabolic Remodeling and Health, Institute of Metabolism and Integrative Biology, Fudan University, Shanghai, China; 11 Qizhi Institute, Shanghai, China; 12 Oxford University Hospitals NHS Trust, Oxford, UK

**Keywords:** Myeloproliferative disease, Cell signalling

## Abstract

Hypoxia-inducible factors (HIFs) are master transcriptional regulators, central to cellular survival in hypoxia and frequently activated within malignancy. Whilst malignant context directs the role of HIFs within oncogenesis, these mechanisms are not well characterised. Applying the JAK2V617F myeloproliferative neoplasms (MPNs) oncogene-driver model, in which HIF-1α is stabilised in normoxia (20% O_2_), we sought to determine whether the modality of HIF-1 activation directs its function. Through direct analysis of hypoxia-activated vs. JAK2V617F-activated HIF-1 at the chromatin, we define a JAK2V617F-HIF-1 regulon that diverges from canonical HIF/hypoxia targets. In a cohort of 172 JAK2V617F-MPN patients, we observe significant association of the JAK2V617F-HIF-1 regulon, but not canonical HIF-1 gene signatures, with disease severity, progression, and patient survival. We further define a subset gene signature (HIF1-MPN-BP) significantly associated with spontaneous transformation to blast phase MPNs. Finally, we identify that JAK2V617F-induced HIF-1α stabilisation is mediated via PIM1 kinase. Our findings demonstrate that HIF-1 activation by the JAK2V617F-PIM1 axis significantly alters HIF-1 transcription function, desensitising HIF-1 activity to cellular oxygen levels, and restricting the HIF-1 regulon to a set of disease-associated target genes within JAK2V617F-MPNs. These findings restore the potential for specific therapeutic targeting of HIF-1 by delineating malignant activation from the physiological hypoxic response.

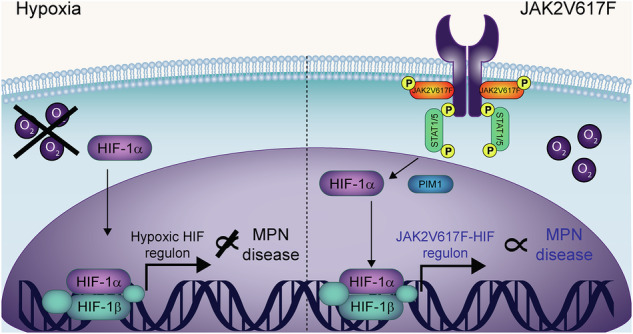

## Introduction

Hypoxia-inducible factors (HIFs) are the major transcriptional regulators of cellular oxygen homoeostasis. The transcription activity of HIFs is regulated by oxygen-dependent degradation of the HIF-1α subunit, via the prolyl hydroxylase domain (PHD) enzymes and von Hippel Lindau (VHL) protein [1]. This mechanism is inhibited by hypoxia, when stabilised HIF-1α protein translocates to the nucleus, heterodimerises with HIF-1β/ARNT [2] and recruits additional transcription coactivators to form an active transcription complex. This binds to hypoxia-response elements (HREs) within target gene promoters [3], initiating the activation of a wide array of genes involved in the response to hypoxia.

Whilst critical to the physiological response to hypoxic exposure of normal cells, the portfolio of genes regulated by HIF provide a competitive advantage to cancer cells. In solid and haematological malignancies, HIFs are overexpressed as a result of two main factors: by hypoxic conditions - such as tumour (TME) or bone marrow (BME) microenvironments- and by genetic mutations, including *VHL*, *p53*, *PTEN*, *PI3K/AKT*, *Ras* and *JAK* [1, 4–8]. In contrast to HIF-2, which is primarily oncogenic in haematological malignancies, HIF-1 confers either oncogenic or tumour suppressive functions in myeloid neoplasia [2, 4, 9, 10]. The underpinning mechanisms that direct the activity of HIF-1 within these malignancies remain poorly understood. HIF-1α was previously shown to be stabilised in cells harbouring the JAK2V617F mutation [4], a highly prevalent mutation within Philadelphia chromosome negative myeloproliferative neoplasms (MPNs) [11–13]. To reconcile the evident context-specificity of HIF-1 activity within malignant haematopoiesis, we sought to determine whether the mechanism of HIF-1 activation can alter its function, using the JAK2V617F model of oncogene-activated HIF-1.

Performing chromatin-immunoprecipitation in matched isogenic cell-lines, we characterised the regulon of HIF-1 activated by hypoxia or by the JAK2V617F oncogene. We identify that the HIF-1 regulon is impaired in the JAK2V617F context compared to hypoxic activation: there is differential intensity of binding at HIF-1 target genes, the repertoire of target-genes is reduced, and HIF-1 gDNA-binding is desensitised to hypoxia. We determine that the JAK2V617F-HIF-1 regulon is significantly associated with disease severity, spontaneous to Blast Phase MPN disease, and overall survival. We also show that HIF-1 is stabilised by PIM1 and undergoes two novel phosphorylation events in the context of the JAK2V617F mutation. Our findings identify that the mechanism of HIF-1 activation can substantially alter its function, revealing how experimental determination of the HIF-1 regulon within a specific malignant context can reveal hitherto unidentified roles within disease pathogenesis.

## Methods

### Chromatin-immunoprecipitation

A total of 5 × 10^7^ BaF3/hMPL hJAK2 WT or hJAK2V617F cells per condition were incubated 6 h in normoxia (20% O_2_) or hypoxia (1% O_2_) (as previously demonstrated for hypoxic treatment of HPSCs [14]). Cells were fixed by the addition of 1% formaldehyde (Cell Signalling Technology) for 10 min at room temperature (RT), then neutralised by the addition of 10x Glycine for 5 min at RT. Samples were then processed using SimpleChIP® Enzymatic Chromatin IP Kit, Magnetic Beads (Cell Signalling Technology) according to the manufacturer’s protocol. ChIP was performed with HIF-1α (D1S7W) XP® Rabbit mAb (Cell Signalling Technology). Samples were split and processed through either next generation sequencing or mass spectrometry to identify HIF-1α binding loci or transcription cofactors respectively. DNA was isolated using a ChIP DNA Clean & Concentrator (Zymo Research). Next generation sequencing was then performed (see Supplementary Methods).

### Patient data

Bulk RNA-sequencing data and clinical annotations from 230 patients with a positive JAK2V617F mutation from the Princess Margaret Cancer Centre MPN cohort [15]. Individual gene expression counts were subjected to VST normalisation using the ‘DESeq2’ R package whereas gene sets were scored using gene set variation analysis (GSVA) using the ‘GSVA’ R Package [16, 17]. Survival analysis was performed using the ‘survival’ and ‘survminer’ packages in R and visualised with Kaplan–Meier plots to depict differences in survival outcomes between patients with high or low gene expression or gene set enrichment. MIPSS70 analysis was performed within 172 patients with available genomic annotations [18]. Haemoglobin levels (g/L) were available for 230 patients. Patients were classified as: normal (Hb ≥120), mild anaemia (Hb 100–119), moderate anaemia (Hb 80–99), or severe anaemia (Hb <80). Additional analysis of clinical AML patient cohorts was also performed (see Supplementary Methods).

### Multiplex fluorescent barcoding and phospho-flow cytometry

UT7/TPO JAK2 WT/V617F cells were cultured as indicated and then starved overnight in 2% FBS RPMI (Roswell Park Memorial Institute (RPMI) 1640 Medium (Gibco)) media with no TPO prior to treatment. Cells were treated as indicated with 2.5 μm SMI-4a (Cayman Chemical) for 6 h, 2 μm Ruxolitinib (Cell Guidance Systems) for 6 h, DMSO (Sigma) vehicle control for 6 h and/or 50 ng/ml TPO (Peprotech) 30 min in either 6 h of hypoxic (1% O_2_) conditions or in normoxia (20% O_2_). Barcoding and flow cytometry was performed as previously described (including antibodies) [19]; see Fig. S7E for gating strategy.

See Supplementary Information for additional Methods.

## Results

### HIF-1α stabilised by JAK2V617F signalling demonstrates aberrant genomic DNA binding and differential co-regulatory protein partners

**Fig. 1:**
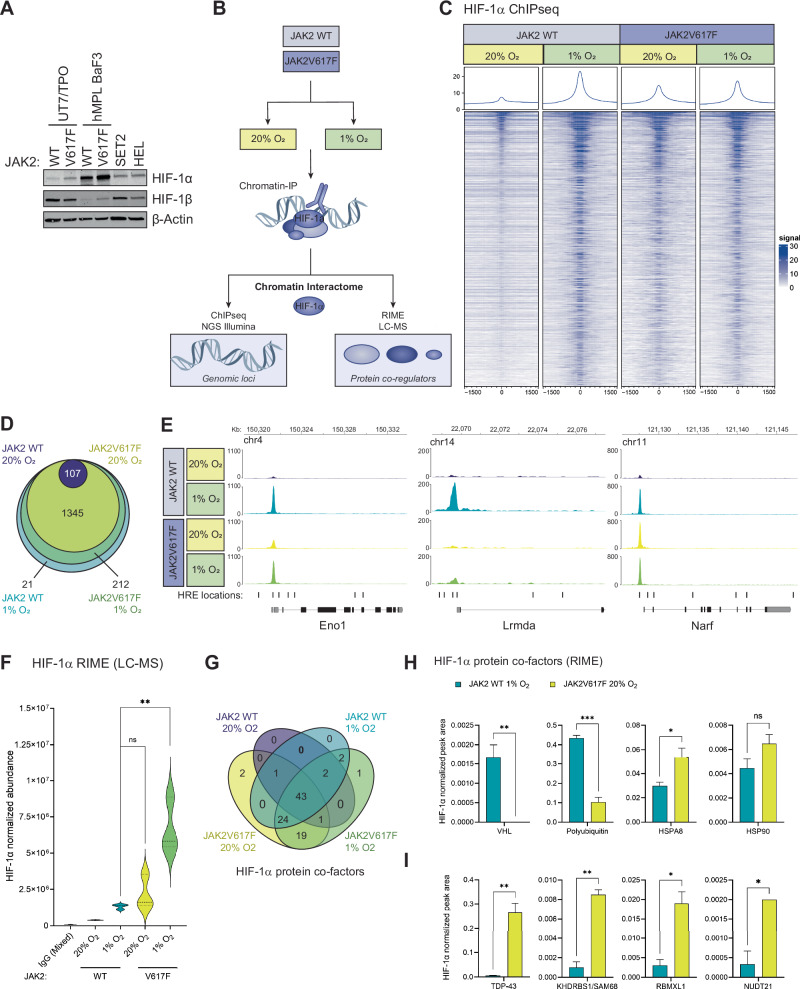
HIF-1α ChIP-seq/RIME in hypoxia- and JAK2V617F-activated conditions. **A** Western blot analysis of the indicated proteins in JAK2 WT and JAK2V617F monogenic BaF3 hMPL cells and UT7/TPO cells, JAK2V617F heterozygous SET2 cells and JAK2V617F homozygous HEL cells cultured in normoxia (20% O_2_). β-actin loading control (data shown representative of *n* = 3). **B** Schematic of paired-sample ChIP-seq and RIME (rapid immunoprecipitation mass spectrometry of endogenous protein) method (*n* = 3 for each method). **C** Heatmap of normalised ChIP-seq HIF-1α occupancy signal for the entire genome. Each row of the heatmap is a genomic region, centred to peaks of occupancy. Occupancy is summarised with a gradient colour code key with 0 representative of no binding (white) to maximum binding 30 (blue). Plots at the top of the heatmaps show the median signal at genomic regions centred at peaks of occupancy. **D** Venn diagram of HIF-1α loci bound in the indicated conditions (WT_Nx, WT_Hx, VF_Nx and VF_Hx respectively), as determined by ChIP-seq, with number of genes unique to each condition labelled. **E** Gene tracks of indicated genomic loci (*x*-axis) denoting strength of HIF-1α binding (*y*-axis) for each of the 4 conditions. **F** Normalised mean total HIF-1α abundance at the chromatin in hMPL BaF3 WT and JAK2V617F cells in normoxia and hypoxia as determined by RIME-mass spec. **G** Venn diagram displaying number of cofactors unique to each condition shown, as determined by RIME (ChIP LC-MS). **H** Normalised abundance of canonical HIF-1α co-factors and **I** RNA-binding and spliceosomal proteins relative to HIF-1α in each condition, as determined by RIME.

To determine whether the transcription activity of HIF-1 is regulated by the mode of HIF-1α stabilisation, we first confirmed the previous observation in 32D cells that the JAK2V617F stabilises HIF-1α in the absence of hypoxia [4] across a range of JAK2V617F^+^ cells (Fig. 1A). Of the cells tested, the BaF3/hMPL isogenic paired cell lines had the highest levels of HIF-1α protein and were therefore selected for further evaluation of the HIF-1α genomic profile. JAK2 WT/JAK2V617F cells were exposed to 20% (normoxia) or 1% O_2_ (hypoxia) for 6 h prior to HIF-1α chromatin immunoprecipitation (ChIP) and analysis by next generation sequencing (ChIP-seq) and mass spectrometry (RIME ChIP-LC-MS) (Fig. 1B). In JAK2 WT cells we observed classical induction of HIF-1α binding to genomic DNA (gDNA) in response to hypoxia (1% O_2_) (Fig. 1C). HIF-1α binding was evident in normoxic JAK2V617F cells, however we observed a significant global reduction of binding across the genome (Figs. 1C and  S1A). Surprisingly, the intensity of HIF-1α gDNA binding was not enhanced by exposure of JAK2V617F cells to hypoxia, even at canonical loci (e.g. *Slc2a1, Slc2a3, Pdk1, Vegfa*) (Fig. S1B). JAK2V617F cells did not contain any unique HIF-1α binding loci: rather, these were a subset of sites bound by HIF-1α in JAK2 WT hypoxia conditions (Fig. 1D).

Whilst absolute HIF-1α peak intensity at canonical sites largely reflected the global intensities of gDNA binding between the conditions (Fig. S1C), for example, at the *Aldoa* promoter (Fig. S1D), we observed groups of genes—both canonical and non-canonical HIF-1 targets—where the pattern of HIF-1α binding intensity across conditions was not proportional to global binding. These included sites where HIF-1α bound at comparable intensity in hypoxia in both WT and JAK2V617F cells (e.g. *Eno1*), sites where no significant binding was detected in JAK2V617F cells in any condition (e.g. *Lrmda*), and sites where HIF-1α bound with equivalent intensity across conditions (e.g. *Narf*) (Fig. 1E). These data indicated that within the global reduction of HIF-1α binding and insensitivity to hypoxia in JAK2V617F cells, redistribution of HIF-1α across genomic loci was also occurring. This was made further evident by ranking the intensity of HIF-1α binding at genomic loci within each condition, which revealed condition-specific preferentiality of sites bound by HIF-1α (Fig. S1E). We also noted differences in DNA feature analysis: HIF-1α bound to more intergenic and intronic regions in JAK2V617F cells (Fig. S1F), and to 14% fewer regions containing HREs (Fig. S1G). To determine whether the changes in binding were due to chromatin accessibility, we cross-analysed our ChIP-seq dataset with a JAK2V617F/WT single cell ATAC-Seq dataset [20]; however no significant differences in accessibility at HIF-1α binding loci were detected (Fig. S1H).

We therefore analysed the paired RIME dataset (ChIP-LC-MS) (Fig. 1B) to determine whether the changes in HIF-1α binding in JAK2V617F cells were mediated by differences in the protein cofactors present in the HIF-1 complex (Table S1). Unexpectedly, despite reduced gDNA-binding (Figs. 1C and  S1A), levels of HIF-1α protein immunoprecipitated from the chromatin were comparable in normoxic JAK2V617F and JAK2 WT hypoxic cells, and were significantly higher in hypoxic JAK2V617F cells (Fig. 1F). In total, 94 protein partners were pulled down with HIF-1α, of which 43 were detected in all four conditions including canonical co-factors HIF-1β/ARNT and HSP90b (Fig. 1G and Table S1). When normalised to HIF-1α abundance, significant reductions of HIF-1α interaction with the ubiquitin-mediated degradation (UMD) markers VHL and polyubiquitin were detected in normoxic JAK2V617F cells, as well as increased interaction with HSP90 and HSPA8/HSP70, which protect HIF-1α from UMD [21] (Fig. 1H). Notably, string analysis of JAK2V617F-HIF-1α co-factors identified enrichment of RNA-binding and spliceosomal factors in both normoxia and hypoxia (Fig. S1I), including significant enrichment of TDP-43, KHDRBS1/SAM68, RBMXL1 and NUDT21 (Fig. 1I).

Taken together, these analyses reveal that activation of HIF-1α via oncogenic JAK2V617F signalling significantly alters the formation and function of the HIF-1 transcription complex compared to physiological activation by hypoxia.

### JAK2V617F-HIF-1 gene-signatures diverge from canonical hypoxia HIF-1 signatures

**Fig. 2:**
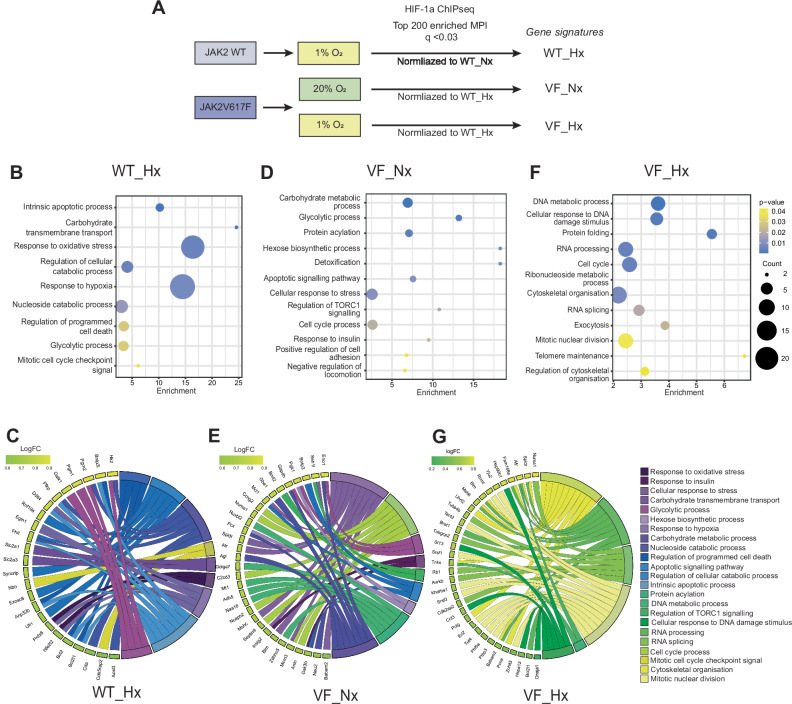
Gene ontology analysis of hypoxia- and JAK2V617F-activated HIF-1α target gene signatures. **A** Top 200-enriched genomic loci from HIF-1α ChIP-seq analysis with *q* < 0.03 for each condition were selected for further analysis, from which the gene signatures were derived. Enrichment of WT_Hx relative to WT_Nx; VF_Nx/VF_Hx relative to WT_Hx. Gene ontology (GO) analysis of WT_Hx (**B**, **C**), VF_Hx (**D**, **E**) and VF_Nx (**F**, **G**) gene signatures displayed as enrichment bubble plot (**B**, **D**, **F**) or chord plot (**C**, **E**, **G**) with designated GO term against enrichment relative to reference gene set (derived in **A**). For bubble plots, GO terms plotted in descending order of statistical significance as designated by *p* value (from 0.01 (blue) to 0.04 (yellow)) with ‘bubble’ size indicating number of genes associated with the indicated function. For chord plots, plotted anticlockwise by gene within signature in descending order of fold change (LogFC) relative to reference geneset. GO function colour-coded by similarity to hypoxia/oxidative stress GO functions: from purple (high) to yellow (low).

HIF-1 was previously reported as a potential therapeutic target in JAK2V617F-MPNs [4]. To determine whether canonical HIF-1/hypoxia genes or JAK2V617F-HIF-1 genes are associated with MPN disease, we identified the top 200 significantly enriched gene loci from our ChIP-seq dataset to create signatures representative of HIF-1 target genes in JAK2 WT hypoxic (WT_Hx), JAK2V617F normoxic (VF_Nx) and JAK2V617F hypoxic (VF_H**x**) cells (Fig. 2A and Table S2). Gene ontology (GO) pathway analysis of the WT_Hx gene signature was recapitulative of the HALLMARK_HYPOXIA signature (Figs. 2B, C and  S2A), a previously published gene signature of 200 HIF-1α hypoxia target genes identified from 87 founder gene sets [22, 23]. These gene signatures were associated with canonical HIF-1 gene pathways, including glycolysis and response to oxidative stress and hypoxia. In contrast, the VF_Nx gene signature included non-canonical pathways such as DNA metabolism, DNA damage response (DDR) and RNA processing and a reduction in classical signature pathways, including glycolysis (Fig. 2D, E). Strikingly, the HIF-1 gene-signature in hypoxic JAK2V617F cells (VF_Hx) separated even further in ontogeny from WT_Hx, with cell cycle progression and checkpoint pathway genes demonstrating the greatest contribution to the signature (Fig. 2F, G). Recently, a HIF target-gene metascore containing a core set of 48 genes targeted by HIF across all cell/cancer types exposed to hypoxia analysed was described [3]. Strikingly, the VF_Nx and VF_Hx HIF-1 signatures comprised only 4.5% (9/200) and 1% (2/200) of the metascore respectively (Fig. S2B), underscoring the divergence of the JAK2V617F HIF-1 regulon from other cancer types.

### Expression of the JAK2V617F-HIF-1 VF_Hx gene signature separates JAK2V617F MPN patients on disease severity and is associated with worsened survival

**Fig. 3:**
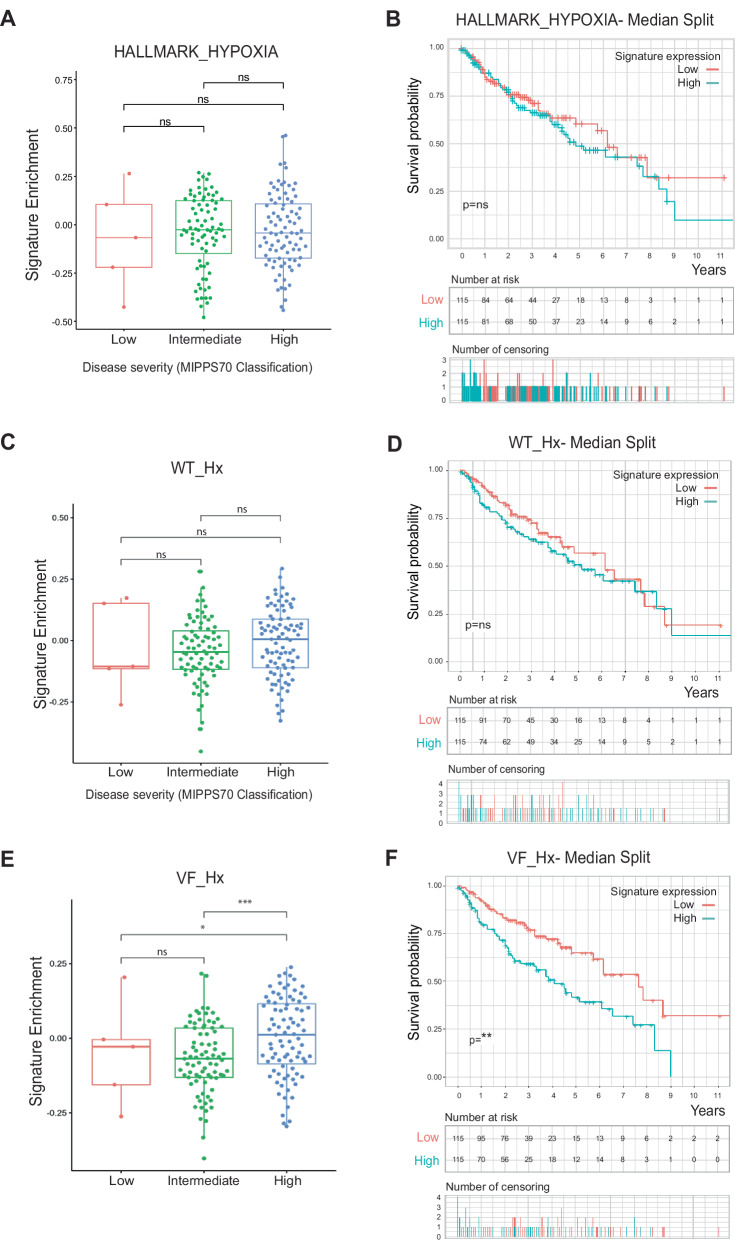
Expression of hypoxia- and JAK2V617F-activated HIF-1α signatures in JAK2V617F MPN patients and overall survival. **A** Gene expression enrichment of canonical HIF-1α (Hallmark_Hypoxia) gene signature in a 172-MPN patient cohort, plotted by MIPSS70 (Mutation-Enhanced International Prognostic Score System for transplantation eligible-aged patients with overt PMF) classification and **B** Kaplan–Meier plot survival curve subdivided into populations with high (above cohort median) (blue) or low (below cohort median) (red) expression of canonical HIF-1α (Hallmark_Hypoxic) gene signature. Overall significance of survival difference is shown. Remaining patients in each population shown in table below with number of censoring (i.e. number of patients leaving the study whose fate is unknown). **C**, **D** As above, but for gene expression enrichment of the WT JAK2 hypoxia HIF-1α gene signature (WT_Hx). **E**, **F** As above, but for gene expression enrichment of the JAK2V617F hypoxia HIF-1α signature (VF_Hx).

We next investigated expression of our HIF-1α gene signatures in a cohort of 172 JAK2V617F MPN patients for which RNAseq, disease severity (MIPSS70 scores) and survival data existed [15]. First, we saw no link between expression of either the HALLMARK-HYPOXIA signature (Fig. 3A, B) or our experimentally determined WT_Hx signature with disease severity or survival of patients (Fig. 3C, D). Of the canonical hypoxia genes, only *VEGFA* and *ENO1* expression in singularity were associated with patient survival (Fig. S3A, B). However, patients with a high disease severity (high MIPSS70 score) had significantly increased expression of the VF_Nx gene signature (Fig. S3C), and this was even more pronounced with the VF_Hx signature (Fig. 3E). Whilst expression (high or low) of the VF_Nx gene signatures was not predictive of patient survival (Fig. S3D), high expression of the VF_Hx gene signature was associated with significantly worse overall survival (Fig. 3F). Furthermore, no correlation existed between anaemia score and VF_Hx signature expression, providing further evidence that JAK2V617F-HIF-1 genes diverge from canonical hypoxia genes (Fig. S3E). These findings reveal that the expression of HIF-1α target-genes in hypoxic JAK2V617F cells correlate with MPN disease severity and a significantly worse survival outcome for JAK2V617F positive patients.

### JAK2V617F-HIF-1 VF_Hx target genes upregulated in megakaryocyte progenitors correlate with MPN disease

**Fig. 4:**
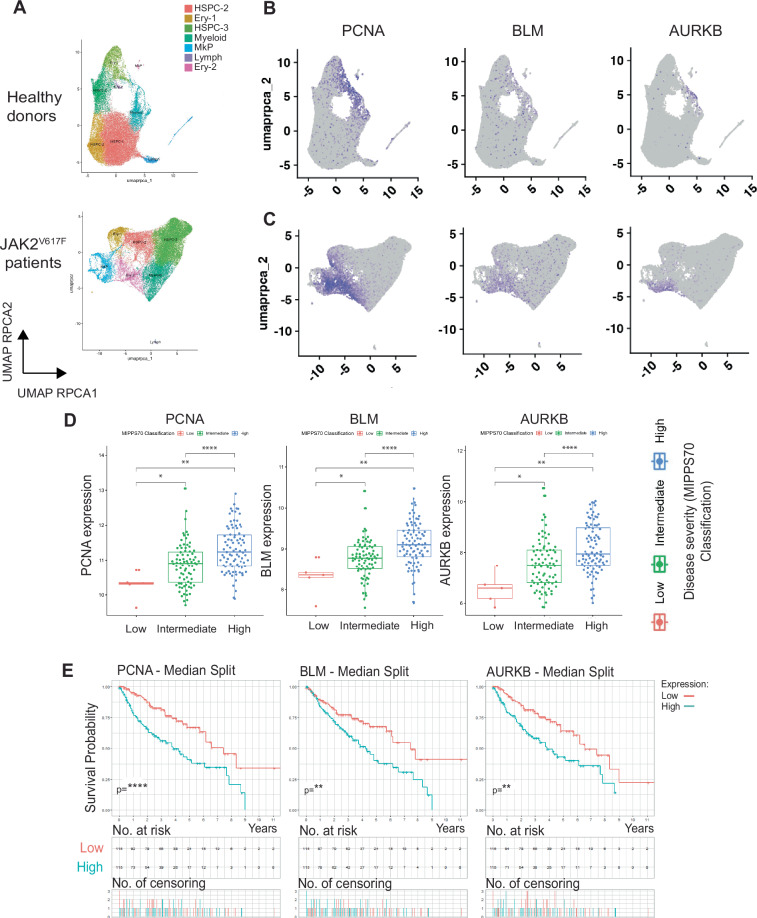
JAK2V617F-HIF-1α target gene expression in HSPC populations and association with MPN disease progression and survival. **A** Dimensionality reduction using Uniform Manifold Approximation and Projection (UMAP) of control healthy donor (*n* = 6) (top) and JAK2V617F-positive MPN patient (*n* = 11) (bottom) cells identified 8 distinct clusters. Cells were partitioned using the Louvain community-detection clustering method and annotated according to expression of lineage signature genes for hematopoietic cell types [26]. Ery (erythroid), MkP (megakaryocyte progenitor), HSPC (hematopoietic stem/progenitor cells), Lymph (lymphoid). **B** Cells on healthy donor and **C** JAK2V617F-positive MPN patient UMAP plots coloured purple according to high expression levels of indicated signature genes. **D** 172-MPN patient cohort gene expression of indicated signature genes plotted by MIPSS70 and **E** cohort Kaplan–Meier plot survival curve subdivided into populations with high (above cohort median) (blue) or low (below cohort median) (red) expression of indicated signature genes.

To identify putative JAK2V617F-HIF-1 induced therapeutic targets, we next investigated the expression of individual genes contained within the VF_Hx signature in patient samples. To do this, we performed an integrated analysis of a bulk RNAseq dataset from JAK2V617F^+^ mouse hematopoietic stem and progenitor cells (HSPC) [24], JAK2V617F myelofibrosis (MF) patient single cell ATACseq [20], JAK2V617F MF patient scRNAseq [25] (Table S4) and the 172 JAK2V617F^+^ patient bulk RNAseq dataset (Fig. S4A; Supplementary Methods). This enabled robust identification of differentially expressed JAK2V617F-HIF-1 genes across biologically motivated datasets (Fig. S4B, C and Table S3), and analysis of their expression across HSPC populations of JAK2V617F MPN patients (Fig. 4A). This analysis revealed that only the VF_Hx signature genes that were differentially expressed in the disease-driving expanded megakaryocyte progenitor populations (MkP/MEP) in JAK2V617F datasets were individually associated with worsened disease severity and survival; for example, *PCNA, BLM, AURKB* (Fig. 4B–E) and *BCL2L1* (Fig. S4D). VF_Hx signature genes that were globally upregulated throughout HSPC clusters, for example *TNKS, HECTD1, WIPF1, PPID* were not correlated with disease severity/survival (Fig. S4E–H). Additionally, we noted a small cohort of genes that were significantly upregulated in more primitive progenitor populations in patients (HSPC3), that were inversely correlated with disease severity and survival, including *IER3*, which regulates cell cycle/apoptosis decisions (Fig. S4I) [26]. These data identify that individual JAK2V617F HIF-1α target genes are associated with MPN disease severity, and that this association is only present when these gene expression changes occur uniquely in disease-driving MkP/MEPs.

### A subset of JAK2V617F-HIF-1 genes correlate with transformation to blast phase MPN

**Fig. 5:**
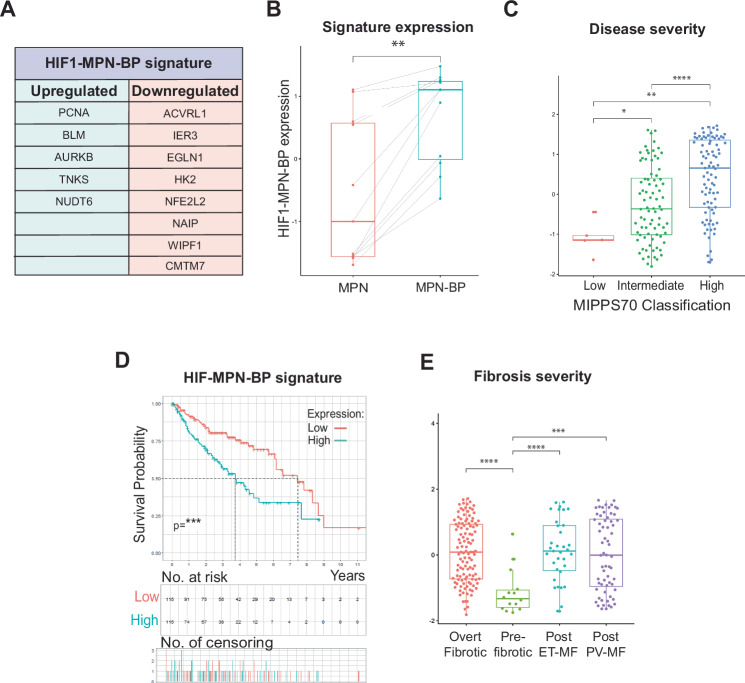
Associated expression of a sub-signature of JAK2V617F-HIF-1 target genes with MPN transformation to blast-phase (MPN-BP). **A** HIF1-MPN-BP gene signature, significantly up- or downregulated in JAK2V617F MPN patients after spontaneous transformation to AML. **B** HIF1-MPN-BP signature enrichment in the 11-patient subset of the 172-MPN patient cohort who spontaneously transformed to AML, plotted by matched expression pre- (MPN) (red) and post-spontaneous AML transformation (MPN-BP) (blue). **C** 172-MPN patient cohort gene expression enrichment of HIF1-MPN-BP signature plotted by MIPSS70 classification and **D** Kaplan–Meier plot survival curve subdivided into populations with high (above cohort median) (blue) or low (below cohort median) (red) expression of HIF1-MPN-BP gene signature. **E** 172-MPN patient cohort gene expression enrichment of HIF MPN-BP signature plotted by patient fibrosis severity grade.

JAK2V617F positive MPN patients who develop myelofibrosis are at risk of spontaneous conversion to blast phase leukaemia (MPN-BP); however, the molecular mechanisms underpinning this pathobiological event are not well characterised [12]. We therefore applied the integrated analysis (Fig. S4A) to the 11 patients from the 172 JAK2V617F MPN patient cohort that transformed to MPN-BP, analysing the expression of the VF_Hx signature genes in matched pre- and post-transformation samples. We identified a HIF1-MPN-BP gene expression signature comprising 13 VF_HX signature genes that were significantly associated with transformation (Figs. 5A, B and  S5A). Within the HIF1-MPN-BP signature, DNA damage response genes remained upregulated, whereas downregulated pathways included negative feedback of the hypoxic response (*EGLN1*) and regulators of glucose metabolism (*IER3, HK2*) (Fig. 5A). There was a strong and significant association of disease severity with enrichment of the HIF1-MPN-BP gene signature (Fig. 5C), along with worsened survival (Fig. 5D) and fibrosis severity (Fig. 5E). Canonical hypoxia genes were not associated with transformation to MPN-BP (Fig. S5B, C). Within healthy bone marrow, expression of the HIF1-MPN-BP signature is enriched on early MEP/MkP and early erythroid populations (Fig. S5D), reflecting MPN cell-of-origin culminating in the expanded megakaryocyte/erythroid populations phenotypical of MPNs. Analysis of the HIF1-MPN-BP signature in the BEAT-AML cohort, showed no significant difference between de novo AML and secondary AML (Fig. S5E), indicating that enrichment of the HIF1-MPN-BP signature is specific to JAK2V617F MPN transformation to BP but not evident in AML secondary to a range of myeloid neoplasia and driver mutations [27]. Interestingly, though, the HIF1-MPN-BP signature was enriched in secondary AML patients with intermediate or high clinical risk (Fig. S6F), however in contrast to MPN-BP, it was not significantly associated with patient survival (data not shown). Together, these data identify that dysregulated expression of 13 JAK2V617F-HIF-1 genes (HIF1-MPN-BP) is significantly enriched through transformation of JAK2V617F MPN to blast phase MPN and is significantly associated with patient survival.

### Novel phosphorylation events occur on HIF-1α stabilised by JAK2V617F signalling

Having identified a non-canonical HIF-1α regulon in JAK2V617F cells which correlates with MPN disease severity and survival, we next sought to determine the mechanisms by which HIF-1α is stabilised and regulated, and whether this alternative regulon activity could be selectively targeted in the context of the JAK2V617F mutation. Given the significance of gene expression changes in patient MkP/MEPs, we selected the human megakaryoblastic cell line UT-7/TPO harbouring either WT JAK2 or the JAK2V617F mutation for these analyses. We treated the UT-7 TPO cell-line pair with the JAK inhibitor Ruxolitinib, an inhibitor of HIF-1α (GN44028) and a HIF activator/PHD inhibitor (Roxadustat) over a time-course at 1% O_2_. We found that all three inhibitors acted indiscriminately upon HIF-1α protein levels between WT and JAK2V617F cells (Fig. S6A) and, did not affect HIF-1α cellular location (Fig. S6B).

**Fig. 6:**
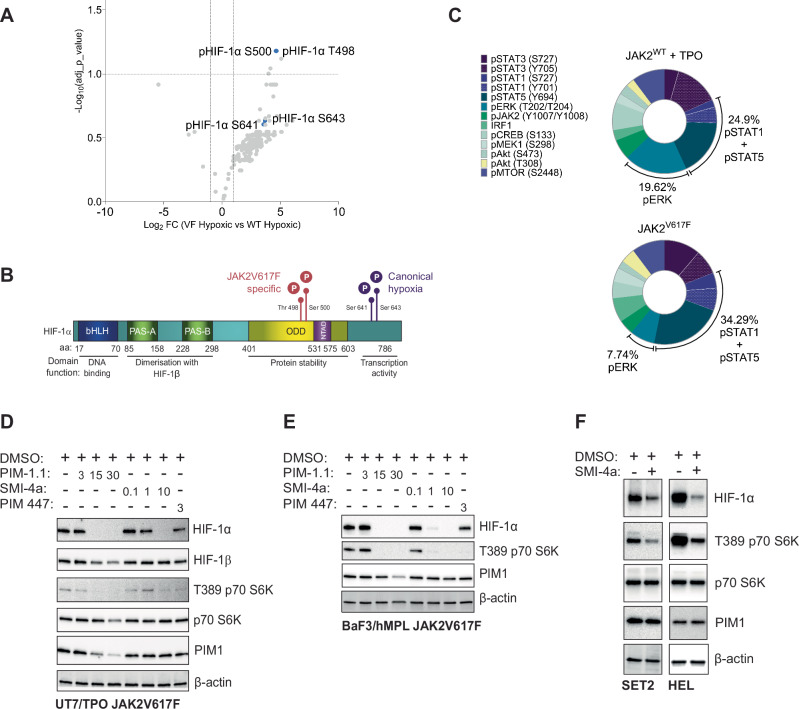
Phosphorylation on T498/S500 in JAK2V617F cells stabilises HIF-1α via PIM1 kinase activity. **A** Volcano plot of HIF-1α phospho-site signal intensity as determined by phospho-mass spectrometry, performed on hypoxic (1% O_2_) treated JAK2V617F and WT JAK2 UT7/TPO cell lysate. Lysate enriched for phosphoproteins by TiO2 and enriched for HIF-1α by immunoprecipitation (*n* = 3). Signal intensity plotted vs. statistical significance (−log10 adj *p* value). **B** HIF-1α protein domain schematic with canonical and JAK2V617F-specific phosphorylated amino acids indicated. **C** Proportional representation analysis of each signalling proteins phosphorylation, relative to total kinase cascade phosphorylation downstream of JAK2, measured by multiplex fluorescent phospho-flow cytometry in JAK2V617F cells and TPO/MPL stimulated JAK2 WT cells. Phosphorylated signalling proteins colour-coded on plot and relative percentage of total signalling made up by combined pSTAT1 and pSTAT5 and pERK signalling labelled. **D** Immunoblot of the indicated proteins in JAK2V617F UT7/TPO or **E** BaF3/hMPL cells starved overnight and treated for 6 h at the indicated dose (μM) with either TCS PIM1.1, SMI-4a, PIM447 or vehicle control. **F** Immunoblot of the indicated proteins in SET2 and HEL cells treated for 6 h with 1 μM SMI-4a or vehicle control. β-actin loading controls; all immunoblot data shown representative of *n* = 3.

As HIF-1α phosphorylation is essential to its transcription activation [28–35], we reasoned that kinase activity downstream of JAK2V617F could be responsible for HIF-1α stabilisation. Phospho-proteomic mass spectrometry analysis of HIF-1α immunoprecipitated from hypoxic UT7/TPO JAK2 WT/V617F cells detected phosphorylation of canonical hypoxia/ERK sites (Ser641/643) in JAK2 WT cells; in JAK2V617F cells, two previously undescribed phosphorylation sites were additionally detected at Thr498/Ser500 of HIF-1α (Figs. 6A, B and  S6C). To identify the kinase(s) responsible, we took an unbiased, high-throughput phospho-flow cytometry approach, characterising the signalling cascades downstream of the JAK2V617F mutation compared to TPO/MPL-mediated wildtype JAK2 signalling (Fig. S6D) [19]. These analyses demonstrated not only a global amplification of JAK2 signalling in JAK2V617F mutant cells but also activation of specific downstream cascades (Fig. S6E). Most notably, STAT1 and STAT5 showed proportionally increased activation (Fig. 6C). We next interrogated STAT1/5 target genes for known HIF-1α kinases [36–39], and identified the CAMK serine/threonine kinase PIM1, which is known to be upregulated in HSPCs harbouring the JAK2V167F mutation [40, 41]. We therefore treated UT7/TPO WT/JAK2V617F and BAF3 WT/JAK2V617F with a panel of PIM inhibitors with the greatest selectivity for PIM1 [42, 43] and observed reduction in HIF-1α protein levels in normoxic JAK2V617F cells by immunoblot with all PIM inhibitors tested (Figs. 6D, E and S6F, G). Of these, SMI-4a demonstrated dose-dependent effects on HIF-1α levels whilst not affecting PIM1 protein levels (thereby indicating specificity for PIM1 kinase activity inhibition [44, 45]) and was selected for further studies. Treatment of JAK2V617F patient-derived cell lines (HEL, SET2) with SMI-4a also reduced normoxic HIF-1α levels (Fig. 6F).

### PIM1 inhibition represses HIF1-MPN-BP gene expression in JAK2V617F cells

**Fig. 7:**
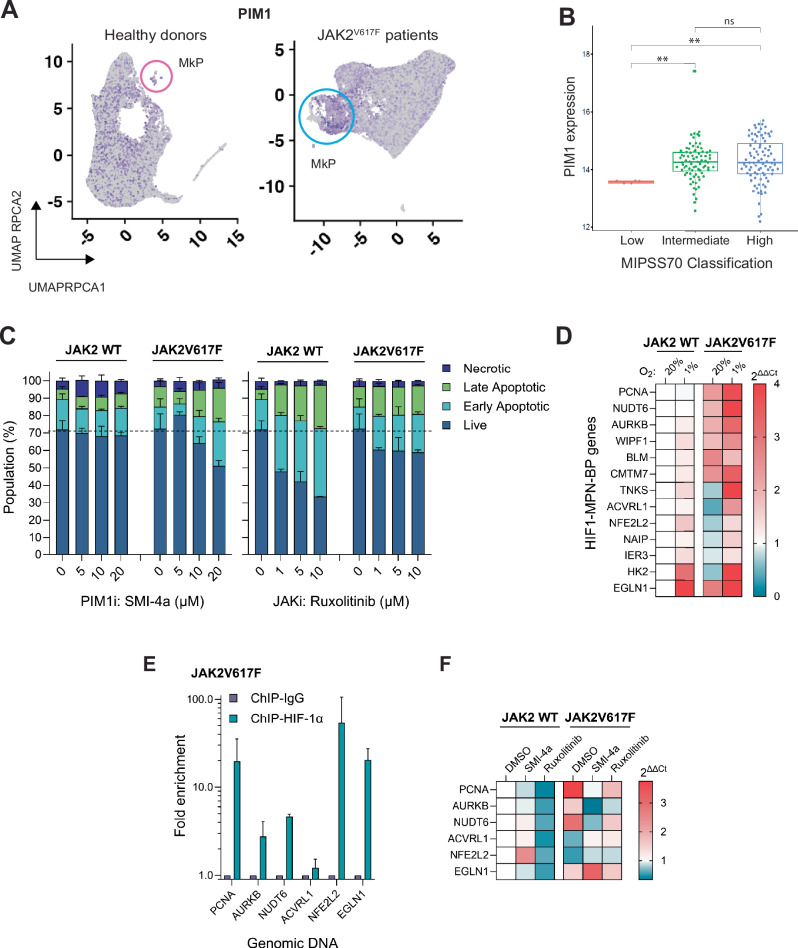
PIM1 inhibition suppresses JAK2V617F-HIF-1 target gene expression without affecting hypoxia-induced HIF-1 activity. **A** HSPCs of healthy donor (left) and JAK2VF-positive MPN patient (right) UMAP plots coloured purple according to high expression levels of PIM1 with megakaryocyte progenitor (MkP) population circled in pink and blue respectively. **B** Gene expression enrichment of PIM1 in the 172-MPN patient cohort plotted by MIPSS70 classification. **C** Percentage of UT7/TPO WT or JAK2V617F cells undergoing the indicated stage of cell death treated with increasing doses of SMI-4a, Ruxolitinib or DMSO control for 72 h in either normoxic (20% O_2_) or hypoxic (1% O_2_) conditions (*n* = 3). **D** HIF1-MPN-BP signature gene expression in UT7/TPO WT and JAK2V617F cells in cultured in normoxic (20% O_2_) or hypoxic (1% O_2_) conditions for 24 h. **E** ChIP-qPCR demonstrating HIF-1α binding levels at HIF1-MPN-BP signature genes. HIF-1α ChIP gDNA isolated from UT7/TPO JAK2V617F cells cultured for 24 h in hypoxic (1% O2) conditions (*n* = 3). **F** Gene expression of HIF1-MPN-BP signature genes in UT7/TPO WT and JAK2V617F cells starved overnight and then treated as indicated with SMI-4a, Ruxolitinib or DMSO control for 24 h in hypoxic (1% O2) conditions (*n* = 3).

PIM1 has been described as a novel therapeutic target in MPNs, due to its upregulation in myelofibrosis patient PBMCs and LT-HSCs of JAK2V617F mice, and its contribution to JAK-inhibitor resistance [40, 41, 46]. We found that PIM1, like the HIF1-MPN-BP signature, was enriched in the MkP/MEP population in compared to healthy donors (Fig. 7A) and PIM1 levels correlated with disease severity (Fig. 7B). To explore whether PIMi afforded greater selectivity to JAK2V617F cells compared to Ruxolitinib, and whether this selectivity extended to inhibiting JAK2V617F-HIF-1 activity, we first characterised the effect of Ruxolitinib or SMI-4a on WT and JAK2V617F cells. PIM1 inhibition was notably less disruptive to global signalling downstream of JAK2 compared to Ruxolitinib (Fig. S7A, B) and resulted in dose-dependent cell death via the apoptosis pathway in JAK2V617F hypoxic cells, whilst it had no effect on JAK2 WT cells, in contrast to Ruxolitinib (Fig. 7C). PHDi did not induce cell death in either genotype, whereas HIF-1i did confer a degree of selectivity toward JAK2V617 F cells (Fig. S7C). We next identified that the HIF1-MPN-BP genes were differentially expressed in UT7/TPO JAK2V617F cells compared to WT, mirroring the relative expression levels in MPN-BP (Figs. 7D and 5). We then experimentally validated by ChIP-qPCR that HIF-1α was significantly enriched at the loci of six HIF1-MPN-BP signature genes in UT7/TPO JAK2V617F hypoxic cells (Fig. 7E). HIF-1α binding to these genes was reduced when cells were treated with either Ruxolitinib or GN44028 (Fig. S7D). Treatment of WT and JAK2V617F cells with either Ruxolitinib or SMI-4a revealed that whilst Ruxolitinib induced changes in HIF1-MPN-BP gene expression in both JAK2 WT and V718F cells, PIM1 inhibition with SMI-4a had a greater rescue effect on HIF1-MPN-BP gene expression changes in JAK2V617F cells than Ruxolitinib, and had little effect on these genes in WT hypoxic cells (Fig. 7F). HIF-1i and PHDi also induced genotype-indiscriminate effects on expression of these genes (Fig. S7E). Together, these data demonstrate that PIM1 stabilises HIF-1α in the context of JAK2V617F-mutated MPNs, and PIM1 inhibition can selectively target expression of JAK2V617F-HIF-1 genes in hypoxic JAK2V617F cells, whilst conferring minimal effects on WT hypoxic cells.

## Discussion

Despite years of promising preclinical work, HIF-1 inhibitors have consistently failed as anti-cancer agents in clinical trials, primarily due to lack of specificity and activation of compensatory signalling mechanisms [47, 48]. With a view to identifying orthogonal approaches to selectively target HIF-1 in the malignant context, our study investigated whether oncogene-activated HIF-1 conferred functions distinct from its physiological hypoxic counterpart, and whether these activation mechanisms could be targeted. Using the JAK2V617F MPN model, our study revealed that the HIF-1 regulon is substantially altered in this context compared to activation by hypoxia.

JAK2V617F-stabilised HIF-1α displayed reduced binding at the chromatin which could not be rescued by hypoxia, suggesting it may be carrying out alternative roles to canonically activated HIF-1α. This hypothesis is supported by our RIME data (ChIP-LC-M/S), which identified differential HIF-1 protein co-factors in JAK2V617F cells, with a notable enrichment of RNA-binding/processing factors. In triple negative breast cancer cell lines, HIF-1α binds TRIM28/DNAPK to release paused RNA Pol II, thereby regulating elongation rate and, it is reasonable to infer, splicing (although this was not investigated) [49]. Taken together with the reduced intensity of HIF-1α binding to gDNA, the identification that HIF-1 binds to intron/exon regions of target genes, and that JAK2V617F-HIF-1 target genes are both up- and down-regulated at the transcript level, our chromatin interactome studies point to a putative peri-transcriptional regulatory role for HIF-1, beyond its canonical transcription initiation, which warrants further investigation.

Proteomic analysis of HIF-1 in JAK2V617F cells revealed two previously undescribed phosphorylation events on HIF-1α within the oxygen dependent degradation (ODD) domain (Thr498/Ser500). The ODD domain of HIF-1α is inherently unstructured [50]; this is a typical feature of transcription factors to enable pliable function in response to co-regulator protein interactors [51]. It is possible that phosphorylation of T498/S500 might sterically block the interaction of the PHD enzymes with their target proline residues (P402/P564) in the ODD, thereby inhibiting oxygen-mediated degradation, as proposed with the Thr455 PIM1-mediated phosphorylation in prostate cancer [52]. Given their relatively distal proximity, it is more probable that phosphorylation at the T498/S500 sites induces an intermediate conformational alteration or co-factor interaction [53–55].

We identified that a JAK2V617F-HIF-1 signature and sub-signature (HIF1-MPN-BP) derived from HIF-1α ChIPseq in hypoxic cells (VF_Hx)—but notably, not normoxic cells (20% O2; VF_Nx)—were significantly associated with JAK2V617F MPN disease progression, patient outcome and transformation to blast phase disease. Physiologically, a JAK2V617F mutant HSPC residing within the bone marrow would be exposed to a hypoxic microenvironment, whereas a circulating cell would be well oxygenated. It is reasonable to deduce, therefore, that bone marrow-residing JAK2V617F HSPCs (including the expanded MkP population) would activate the VF_Hx HIF-1 gene signature. This is in keeping with our observation that only JAK2V617F-HIF-1 genes dysregulated in the MkP population are also associated with patient outcome. We note that the JAK2V617F-HIF-1 regulon and HIF1-MPN-BP signatures do not have any genes in common with the prognostic MPN24/MPN13 signatures derived from the same dataset [15]. Therefore, whilst genes induced by HIF-1 in JAK2V617F cells correlate with MPN disease progression and survival, these genes do not represent those with the greatest prognostic value for patients.

DNA damage response (DDR) genes were prominent within the JAK2V617F-HIF-1 regulon. DDR genes have been described as upregulated in de novo AML, where inhibitors of PCNA, BLM and AURKB have shown therapeutic benefit [56–58]. To our knowledge, DDR genes have not previously been associated with transformation to MPN-BP and are yet to be explored as therapeutic targets. Inhibition of other DDR genes (PARP, Polθ and Wee1) suppress the TET2/CALR mutant MPN phenotype in vitro [24, 59, 60]; DDR-targeting drugs may therefore confer therapeutic benefit to MPN patients with high DDR-gene expression at risk of transformation to MPN-BP.

Our data identify the serine-threonine kinase PIM1 as the key factor responsible for the oxygen-independent stabilisation of HIF-1α in JAK2V617F MPNs. We observed that PIM1 is overexpressed within the disease-driving Megakaryocyte/Erythroid Progenitor (MkP) population of MPN patients, and PIM1 transcript levels show a positive correlation with disease severity. Critically, inhibiting PIM1 resulted in the removal of the JAK2V617F-stabilised HIF-1α whilst sparing physiological HIF-1 activity in response to hypoxia; this mechanism may contribute toward the broader anti-proliferative and pro-apoptotic effects of PIM inhibitors (such as TP-3654, currently in clinical trials) [46]. Given the documented feed-forward crosstalk between PIM1 and HIF-1 in solid tumours [61, 62], future work should assess whether the hypoxic BME contributes to regulating this axis in myeloid neoplasia, and also investigate whether PIM1 overexpression in MPNs dysregulates other transcriptional networks, such as the MYC oncogene.

Our study identifies a distinction between the regulon of HIF-1 induced in response to hypoxia and that induced by HIF-1 in response to oncogenic signalling pathways, in the context of JAK2V617F MPNs. These findings restore the potential for therapeutics that can target HIF-1 through exploiting malignant mechanisms of stabilisation from the physiological hypoxic response and essential oxygen homoeostatic mechanisms. Overall, our study rationalises future investigations into both the malignant co-option of other transcription factors in JAK2V617F MPNs and the hypoxia-independent activation of HIF-1 and arising regulons in the pathogenesis of other haematological and solid cancers.

## Supplementary information

Supplementary Table S1

Supplementary Table S2

Supplementary Table S3

Supplementary Table S4

Supplementary Information

## Data Availability

Data for this study are available at NCBI SRA PRJNA1144472.
