# Supplementary material for: JAK2V617F reprograms Hypoxia Inducible Factor-1 to induce a non-canonical hypoxia regulon in myeloproliferative neoplasms
Source: Leukemia. 2026 Feb 2;40(3):609–21. doi: 10.1038/s41375-025-02843-9 (PMC12960217; doi:10.1038/s41375-025-02843-9)
Supplement: Supplementary file 1 — Supplementary Table S1 [file 41375_2025_2843_MOESM1_ESM.pdf]

Table S1.BaF3/hMPL HIF-1a RIME LC-MS

D799

ID Filter = Perc to <1% FDR and min 2 pepitdes

Accession	Description	Mass	Peptide count	Unique peptides	Anova (p)	Rank	H&B q-value	Max fold change	Log 2 max Fold Change	Power
Q80TA9	Ectopic P granules protein 5 homolog OS=Mus musculus GN=Epg5 PE=1 SV=2	290615	8	7	4.05E-10	1	4.46E-08	30.6	4.9	1.00
P0CG49	Polyubiquitin-B OS=Mus musculus GN=Ubb PE=2 SV=1	34348	4	4	1.65E-08	2	9.07E-07	87.7	6.5	1.00
Q61221;Q0VBL6	Hypoxia-inducible factor 1-alpha OS=Mus musculus GN=Hif1a PE=1 SV=3	93457	21	21	2.13E-08	3	7.83E-07	78.1	6.3	1.00
P62862	40S ribosomal protein S30 OS=Mus musculus GN=Fau PE=1 SV=1	6644	4	4	2.68E-08	4	7.37E-07	18.2	4.2	1.00
Q921F2	TAR DNA-binding protein 43 OS=Mus musculus GN=Tardbp PE=1 SV=1	44519	6	6	2.82E-07	5	6.20E-06	242.6	7.9	1.00
Q3UPP8	Centrosomal protein of 63 kDa OS=Mus musculus GN=Cep63 PE=1 SV=2	80399	5	5	4.00E-07	6	7.33E-06	62.6	6.0	1.00
Q9D4H8	Cullin-2 OS=Mus musculus GN=Cul2 PE=1 SV=2	86821	7	7	6.70E-07	7	1.05E-05	24.7	4.6	1.00
Q91W39	Nuclear receptor coactivator 5 OS=Mus musculus GN=Ncoa5 PE=1 SV=1	65280	12	12	6.94E-07	8	9.54E-06	53.3	5.7	1.00
P47911	60S ribosomal protein L6 OS=Mus musculus GN=Rpl6 PE=1 SV=3	33489	7	7	1.27E-06	9	1.55E-05	5.2	2.4	1.00
P63017;P17156;P16627	Heat shock cognate 71 kDa protein OS=Mus musculus GN=Hspa8 PE=1 SV=1	70827	9	8	2.12E-06	10	2.33E-05	14.6	3.9	1.00
Q9D1R9	60S ribosomal protein L34 OS=Mus musculus GN=Rpl34 PE=1 SV=2	13284	2	2	4.59E-06	11	4.59E-05	6.6	2.7	1.00
P23591	GDP-L-fucose synthase OS=Mus musculus GN=Tsta3 PE=1 SV=3	35855	3	3	6.15E-06	12	5.63E-05	6.8	2.8	1.00
P62911	60S ribosomal protein L32 OS=Mus musculus GN=Rpl32 PE=1 SV=2	15850	4	4	6.45E-06	13	5.46E-05	3.4	1.7	1.00
P62827;Q61820	GTP-binding nuclear protein Ran OS=Mus musculus GN=Ran PE=1 SV=3	24408	3	3	1.90E-05	15	1.40E-04	39.2	5.3	1.00
P53762;Q80TR8	Aryl hydrocarbon receptor nuclear translocator OS=Mus musculus GN=Arnt PE=1 SV=3	86908	19	18	2.02E-05	16	1.39E-04	4.1	2.0	1.00
P11499;Q9CQN1;Q9D5S7	Heat shock protein HSP 90-beta OS=Mus musculus GN=Hsp90ab1 PE=1 SV=3	83229	9	5	4.60E-05	17	2.98E-04	15.2	3.9	1.00
P70362	Ubiquitin recognition factor in ER-associated degradation protein 1 OS=Mus musculus GN=Ufd1	34459	2	2	4.79E-05	18	2.93E-04	29.6	4.9	1.00
Q61464	Zinc finger protein 638 OS=Mus musculus GN=Znf638 PE=1 SV=2	217999	2	2	7.29E-05	19	4.22E-04	17.9	4.2	1.00
P55937	Golgin subfamily A member 3 OS=Mus musculus GN=Golga3 PE=1 SV=3	167118	61	60	7.80E-05	20	4.29E-04	22.7	4.5	1.00
P16858	Glyceraldehyde-3-phosphate dehydrogenase OS=Mus musculus GN=Gapdh PE=1 SV=2	35787	8	8	8.58E-05	21	4.50E-04	9.1	3.2	1.00
A2AUM9	Centrosomal protein of 152 kDa OS=Mus musculus GN=Cep152 PE=1 SV=1	196411	5	4	8.71E-05	22	4.36E-04	571.3	9.2	1.00
O09167	60S ribosomal protein L21 OS=Mus musculus GN=Rpl21 PE=1 SV=3	18550	2	2	9.39E-05	23	4.49E-04	4.9	2.3	1.00
Q5SNZ0	Girdin OS=Mus musculus GN=Ccdc88a PE=1 SV=2	215786	3	3	0.000118664	24	5.44E-04	24.1	4.6	1.00
P10126;P62631	Elongation factor 1-alpha 1 OS=Mus musculus GN=Eef1a1 PE=1 SV=3	50082	9	9	0.000118769	25	5.23E-04	6.8	2.8	1.00
Q91VM5;Q9WV02	RNA binding motif protein, X-linked-like-1 OS=Mus musculus GN=Rbmxl1 PE=1 SV=1	42136	2	2	0.000172412	26	7.29E-04	15.5	4.0	1.00
P48725	Pericentrin OS=Mus musculus GN=Pcnt PE=1 SV=2	329262	3	3	0.000220925	27	9.00E-04	163.5	7.4	1.00
Q9D945	Protein LLP homolog OS=Mus musculus GN=Llph PE=1 SV=1	15381	3	3	0.000227147	28	8.92E-04	3.4	1.8	1.00
P20152;P31001;P08551	Vimentin OS=Mus musculus GN=Vim PE=1 SV=3	53655	13	13	0.000268328	29	1.02E-03	11.8	3.6	1.00
Q60749	KH domain-containing, RNA-binding, signal transduction-associated protein 1 OS=Mus musculus	48340	3	3	0.000444502	30	1.63E-03	56.0	5.8	1.00
Q8VEK3	Heterogeneous nuclear ribonucleoprotein U OS=Mus musculus GN=Hnrnpu PE=1 SV=1	87863	5	5	0.000471722	31	1.67E-03	3.9	1.9	1.00
Q8BL97	Serine/arginine-rich splicing factor 7 OS=Mus musculus GN=Srsf7 PE=1 SV=1	30799	4	3	0.000505947	32	1.74E-03	5.6	2.5	1.00
P61358	60S ribosomal protein L27 OS=Mus musculus GN=Rpl27 PE=1 SV=2	15788	3	3	0.000568994	33	1.90E-03	3.7	1.9	1.00
P62908	40S ribosomal protein S3 OS=Mus musculus GN=Rps3 PE=1 SV=1	26657	3	3	0.000695329	34	2.25E-03	7.4	2.9	1.00
Q9D0E1	Heterogeneous nuclear ribonucleoprotein M OS=Mus musculus GN=Hnrnpm PE=1 SV=3	77597	3	3	0.000710666	35	2.23E-03	8.0	3.0	1.00
P47964	60S ribosomal protein L36 OS=Mus musculus GN=Rpl36 PE=3 SV=2	12208	2	2	0.000798401	36	2.44E-03	3.0	1.6	1.00
P99024;P68372;Q7TMM9	Tubulin beta-5 chain OS=Mus musculus GN=Tubb5 PE=1 SV=1	49639	6	6	0.001043492	37	3.10E-03	14.1	3.8	0.99
Q9D8E6	60S ribosomal protein L4 OS=Mus musculus GN=Rpl4 PE=1 SV=3	47124	4	4	0.001208399	38	3.50E-03	4.9	2.3	0.99
P05213;P05214	Tubulin alpha-1B chain OS=Mus musculus GN=Tuba1b PE=1 SV=2	50120	4	4	0.001239806	39	3.50E-03	9.1	3.2	0.99
P62245	40S ribosomal protein S15a OS=Mus musculus GN=Rps15a PE=1 SV=2	14830	3	3	0.001462802	40	4.02E-03	9.2	3.2	0.99
P62830	60S ribosomal protein L23 OS=Mus musculus GN=Rpl23 PE=1 SV=1	14856	2	2	0.001505489	41	4.04E-03	7.0	2.8	0.99
P62702	40S ribosomal protein S4, X isoform OS=Mus musculus GN=Rps4x PE=1 SV=2	29579	2	2	0.001596521	42	4.18E-03	4.2	2.1	0.99
P62751	60S ribosomal protein L23a OS=Mus musculus GN=Rpl23a PE=1 SV=1	17684	6	6	0.001642893	43	4.20E-03	2.3	1.2	0.99
P62849	40S ribosomal protein S24 OS=Mus musculus GN=Rps24 PE=1 SV=1	15413	2	2	0.001952115	44	4.88E-03	10.5	3.4	0.98
P14115	60S ribosomal protein L27a OS=Mus musculus GN=Rpl27a PE=1 SV=5	16595	4	4	0.001960423	45	4.79E-03	3.2	1.7	0.98
Q01853;Q3UMC0	Transitional endoplasmic reticulum ATPase OS=Mus musculus GN=Vcp PE=1 SV=4	89266	4	4	0.002141545	46	5.12E-03	280.0	8.1	0.98
Q6ZWN5	40S ribosomal protein S9 OS=Mus musculus GN=Rps9 PE=1 SV=3	22578	4	4	0.00226841	47	5.31E-03	4.7	2.2	0.98
Q9CXW4	60S ribosomal protein L11 OS=Mus musculus GN=Rpl11 PE=1 SV=4	20240	2	2	0.002379529	48	5.45E-03	18.3	4.2	0.98
P24547	Inosine-5'-monophosphate dehydrogenase 2 OS=Mus musculus GN=Impdh2 PE=1 SV=2	55780	3	3	0.002433499	49	5.46E-03	88.3	6.5	0.98
P27659	60S ribosomal protein L3 OS=Mus musculus GN=Rpl3 PE=1 SV=3	46081	3	3	0.002506463	50	5.51E-03	3.8	1.9	0.98
P68134;P68033;P62737	Actin, alpha skeletal muscle OS=Mus musculus GN=Acta1 PE=1 SV=1	42024	11	2	0.003675238	51	7.93E-03	3.1	1.7	0.96
P14131	40S ribosomal protein S16 OS=Mus musculus GN=Rps16 PE=1 SV=4	16435	2	2	0.003702919	52	7.83E-03	6.9	2.8	0.96
P62892	60S ribosomal protein L39 OS=Mus musculus GN=Rpl39 PE=1 SV=2	6403	2	2	0.004308232	53	8.94E-03	2.5	1.3	0.95
P14148	60S ribosomal protein L7 OS=Mus musculus GN=Rpl7 PE=1 SV=2	31400	5	5	0.004474136	54	9.11E-03	4.4	2.1	0.95
Q8BG05;Q9CX86	Heterogeneous nuclear ribonucleoprotein A3 OS=Mus musculus GN=Hnrnpa3 PE=1 SV=1	39628	6	5	0.004526394	55	9.05E-03	4.0	2.0	0.95
P62918	60S ribosomal protein L8 OS=Mus musculus GN=Rpl8 PE=1 SV=2	28007	2	2	0.004556178	56	8.95E-03	3.5	1.8	0.95
P12970	60S ribosomal protein L7a OS=Mus musculus GN=Rpl7a PE=1 SV=2	29958	4	4	0.004598989	57	8.88E-03	3.9	2.0	0.95
Q9CZM2	60S ribosomal protein L15 OS=Mus musculus GN=Rpl15 PE=2 SV=4	24131	2	2	0.004813676	58	9.13E-03	4.4	2.1	0.95
P62281	40S ribosomal protein S11 OS=Mus musculus GN=Rps11 PE=1 SV=3	18419	4	4	0.006511118	59	1.21E-02	9.9	3.3	0.93
P35700	Peroxiredoxin-1 OS=Mus musculus GN=Prdx1 PE=1 SV=1	22162	2	2	0.006629766	60	1.22E-02	8.3	3.1	0.93
P25444	40S ribosomal protein S2 OS=Mus musculus GN=Rps2 PE=1 SV=3	31212	2	2	0.006642496	61	1.20E-02	5.3	2.4	0.93
P60710	Actin, cytoplasmic 1 OS=Mus musculus GN=Actb PE=1 SV=1	41710	14	3	0.006710923	62	1.19E-02	4.0	2.0	0.92
P58252	Elongation factor 2 OS=Mus musculus GN=Eef2 PE=1 SV=2	95253	4	3	0.008126676	63	1.42E-02	9.0	3.2	0.91
P47963	60S ribosomal protein L13 OS=Mus musculus GN=Rpl13 PE=1 SV=3	24290	3	3	0.012888121	64	2.22E-02	2.9	1.5	0.86
P14733	Lamin-B1 OS=Mus musculus GN=Lmnb1 PE=1 SV=3	66745	2	2	0.01374845	65	2.33E-02	7.4	2.9	0.85
O88569	Heterogeneous nuclear ribonucleoproteins A2/B1 OS=Mus musculus GN=Hnrnpa2b1 PE=1 SV=2	37380	4	3	0.014922482	66	2.49E-02	11.9	3.6	0.84
Q9CR57	60S ribosomal protein L14 OS=Mus musculus GN=Rpl14 PE=1 SV=3	23549	2	2	0.016557732	67	2.72E-02	3.2	1.7	0.83
Q9CQF3	Cleavage and polyadenylation specificity factor subunit 5 OS=Mus musculus GN=Nudt21 PE=1 SV	26224	3	3	0.017115566	68	2.77E-02	13.0	3.7	0.83
Q8BP67	60S ribosomal protein L24 OS=Mus musculus GN=Rpl24 PE=1 SV=2	17768	4	4	0.025813031	69	4.12E-02	2.7	1.4	0.77
P40338	von Hippel-Lindau disease tumor suppressor OS=Mus musculus GN=Vhl PE=1 SV=1	20757	2	2	0.034531361	70	5.43E-02	25.7	4.7	0.72
P56959	RNA-binding protein FUS OS=Mus musculus GN=Fus PE=1 SV=1	52642	2	2	0.039820301	71	6.17E-02	9.8	3.3	0.69
P61979	Heterogeneous nuclear ribonucleoprotein K OS=Mus musculus GN=Hnrnpk PE=1 SV=1	50944	2	2	0.049951815	73	7.53E-02	7.5	2.9	0.65
P02301	Histone H3.3C OS=Mus musculus GN=H3f3c PE=3 SV=3	15306	2	2	0.055372431	74	8.23E-02	1.9	0.9	0.63
P14206	40S ribosomal protein SA OS=Mus musculus GN=Rpsa PE=1 SV=4	32817	2	2	0.061804399	75	9.06E-02	4.3	2.1	0.61
Q99020	Heterogeneous nuclear ribonucleoprotein A/B OS=Mus musculus GN=Hnrnpab PE=1 SV=1	30812	2	2	0.066439563	76	9.62E-02	22.9	4.5	0.60
P35550;Q80WS3	rRNA 2'-O-methyltransferase fibrillarin OS=Mus musculus GN=Fbl PE=1 SV=2	34286	5	5	0.067677591	77	9.67E-02	10.1	3.3	0.59
P97351	40S ribosomal protein S3a OS=Mus musculus GN=Rps3a PE=1 SV=3	29866	3	3	0.069352326	78	9.78E-02	1.6	0.7	0.59
P43275	Histone H1.1 OS=Mus musculus GN=Hist1h1a PE=1 SV=2	21772	4	2	0.080327365	79	1.12E-01	8.5	3.1	0.56
P62242	40S ribosomal protein S8 OS=Mus musculus GN=Rps8 PE=1 SV=2	24190	7	7	0.090990399	80	1.25E-01	2.3	1.2	0.54
P54728	UV excision repair protein RAD23 homolog B OS=Mus musculus GN=Rad23b PE=1 SV=2	43486	2	2	0.098720895	81	1.34E-01	117.7	6.9	0.52
P49312	Heterogeneous nuclear ribonucleoprotein A1 OS=Mus musculus GN=Hnrnpa1 PE=1 SV=2	34175	5	4	0.112552605	82	1.51E-01	5.3	2.4	0.49
P43276	Histone H1.5 OS=Mus musculus GN=Hist1h1b PE=1 SV=2	22562	2	2	0.112717364	83	1.49E-01	2.9	1.5	0.49
P35980	60S ribosomal protein L18 OS=Mus musculus GN=Rpl18 PE=1 SV=3	21631	7	7	0.11363439	84	1.49E-01	2.4	1.3	0.49
P47915	60S ribosomal protein L29 OS=Mus musculus GN=Rpl29 PE=1 SV=2	17576	2	2	0.141957656	85	1.84E-01	1.9	0.9	0.44
Q6ZWV7	60S ribosomal protein L35 OS=Mus musculus GN=Rpl35 PE=1 SV=1	14544	2	2	0.152166192	86	1.95E-01	2.5	1.3	0.43
Q61937	Nucleophosmin OS=Mus musculus GN=Npm1 PE=1 SV=1	32540	4	4	0.154260964	87	1.95E-01	3.9	1.9	0.43
P84099	60S ribosomal protein L19 OS=Mus musculus GN=Rpl19 PE=1 SV=1	23451	2	2	0.155409293	88	1.94E-01	2.6	1.4	0.43
P09405	Nucleolin OS=Mus musculus GN=Ncl PE=1 SV=2	76677	3	3	0.179709121	89	2.22E-01	4.2	2.1	0.40
Q6ZWY9;P70696;Q64524	Histone H2B type 1-C/E/G OS=Mus musculus GN=Hist1h2bc PE=1 SV=3	13898	7	7	0.192510677	91	2.33E-01	2.1	1.1	0.38
P62806	Histone H4 OS=Mus musculus GN=Hist1h4a PE=1 SV=2	11360	9	9	0.204066889	92	2.44E-01	2.2	1.1	0.37
Q7TPV4	Myb-binding protein 1A OS=Mus musculus GN=Mybbp1a PE=1 SV=2	151942	2	2	0.272829911	93	3.23E-01	6.8	2.8	0.31
Q9Z2K1;Q8CCX5	Keratin, type I cytoskeletal 16 OS=Mus musculus GN=Krt16 PE=1 SV=3	51574	11	4	0.278777149	94	3.26E-01	7.0	2.8	0.31
P07356	Annexin A2 OS=Mus musculus GN=Anxa2 PE=1 SV=2	38652	2	2	0.391876103	95	4.54E-01	3.2	1.7	0.24
Q8VIJ6;Q99K48	Splicing factor, proline- and glutamine-rich OS=Mus musculus GN=Sfpq PE=1 SV=1	75394	3	3	0.45084349	96	5.17E-01	5.6	2.5	0.21
C0HKE1	Histone H2A type 1-B OS=Mus musculus GN=Hist1h2ab PE=1 SV=1	14127	6	2	0.600262634	98	6.74E-01	3.7	1.9	0.16
Q9Z315	U4/U6.U5 tri-snRNP-associated protein 1 OS=Mus musculus GN=Sart1 PE=1 SV=1	90830	2	2	0.639853545	101	6.97E-01	1.7	0.8	0.15
Q02257	Junction plakoglobin OS=Mus musculus GN=Jup PE=1 SV=3	81749	5	5	0.655363473	102	7.07E-01	3.4	1.8	0.15
P17225	Polypyrimidine tract-binding protein 1 OS=Mus musculus GN=Ptbp1 PE=1 SV=2	56443	4	2	0.774898348	103	8.28E-01	7.9	3.0	0.12
E9Q557	Desmoplakin OS=Mus musculus GN=Dsp PE=1 SV=1	332706	4	3	0.951056492	110	9.51E-01	1.7	0.7	0.07

		Normalized abundance																	
		Isotype			WT Normoxic			WT Hypoxic			VF Normoxic			VF Hypoxic			Sum	Isotype	WT_Nx
Highest mean condition	Lowest mean condition	D799_1	D799_2	D799_3	D799_4	D799_5	D799_6	D799_7	D799_8	D799_9	D799_10	D799_11	D799_12	D799_13	D799_14	D799_15	Sum	Column0	Column1
VF Hypoxic	Isotype	9.79E+03	9.95E+03	8.14E+03	8.48E+04	8.42E+04	6.58E+04	1.05E+05	9.23E+04	7.05E+04	2.59E+05	3.04E+05	2.24E+05	2.84E+05	3.13E+05	2.58E+05	2.17E+06	9.30E+03	7.82E+04
VF Hypoxic	Isotype	1.86E+04	1.22E+04	3.18E+04	5.10E+04	9.46E+04	7.56E+04	4.51E+05	6.19E+05	6.40E+05	1.83E+05	2.02E+05	1.93E+05	1.85E+06	1.72E+06	1.93E+06	8.07E+06	2.09E+04	7.37E+04
VF Hypoxic	Isotype	8.89E+04	7.88E+04	9.00E+04	3.74E+05	4.09E+05	3.83E+05	1.11E+06	1.39E+06	1.42E+06	1.41E+06	1.60E+06	3.54E+06	5.44E+06	5.82E+06	8.86E+06	3.20E+07	8.59E+04	3.89E+05
VF Normoxic	WT Normoxic	7.67E+06	7.82E+06	1.28E+07	8.52E+05	7.91E+05	1.22E+06	1.01E+06	1.42E+06	7.84E+05	1.46E+07	1.93E+07	1.83E+07	1.32E+07	1.36E+07	1.76E+07	1.31E+08	9.42E+06	9.53E+05
VF Normoxic	Isotype	1.13E+03	8.39E+02	2.34E+03	5.30E+03	5.04E+03	9.05E+03	5.13E+03	1.18E+04	6.67E+03	3.24E+05	4.86E+05	2.36E+05	3.56E+05	4.80E+05	1.10E+05	2.04E+06	1.44E+03	6.46E+03
WT Normoxic	Isotype	3.17E+02	5.09E+02	1.36E+02	2.35E+04	2.45E+04	1.21E+04	1.94E+04	1.44E+04	1.58E+04	9.59E+03	9.49E+03	6.93E+03	1.51E+04	1.19E+04	9.68E+03	1.73E+05	3.21E+02	2.01E+04
VF Hypoxic	Isotype	3.36E+03	3.94E+03	1.75E+03	4.13E+03	2.77E+03	3.66E+03	5.99E+03	1.41E+04	7.26E+03	6.33E+03	6.47E+03	6.63E+03	7.46E+04	7.96E+04	6.95E+04	2.90E+05	3.01E+03	3.52E+03
VF Normoxic	Isotype	1.31E+04	1.53E+04	2.08E+04	2.16E+05	1.76E+05	9.02E+04	9.62E+04	2.61E+05	1.12E+05	8.63E+05	1.08E+06	6.77E+05	6.90E+05	6.89E+05	5.08E+05	5.51E+06	1.64E+04	1.61E+05
WT Hypoxic	Isotype	2.41E+04	2.35E+04	2.01E+04	1.27E+05	1.06E+05	1.01E+05	1.02E+05	1.37E+05	1.11E+05	3.88E+04	5.63E+04	3.64E+04	8.38E+04	5.98E+04	8.38E+04	1.11E+06	2.26E+04	1.12E+05
VF Hypoxic	Isotype	1.98E+04	1.86E+04	2.84E+04	2.23E+04	2.52E+04	3.63E+04	3.15E+04	6.94E+04	4.54E+04	8.35E+04	1.46E+05	1.72E+05	3.17E+05	3.34E+05	3.25E+05	1.67E+06	2.23E+04	2.79E+04
VF Hypoxic	Isotype	5.33E+03	7.16E+03	9.98E+03	1.47E+04	1.33E+04	1.19E+04	1.67E+04	2.29E+04	1.54E+04	2.33E+04	2.64E+04	2.13E+04	5.39E+04	4.03E+04	5.35E+04	3.36E+05	7.49E+03	1.33E+04
VF Hypoxic	Isotype	1.45E+04	1.18E+04	1.58E+04	5.44E+04	5.12E+04	4.18E+04	4.28E+04	4.06E+04	5.83E+04	6.51E+04	7.76E+04	5.27E+04	1.09E+05	1.12E+05	6.55E+04	8.13E+05	1.40E+04	4.91E+04
VF Hypoxic	WT Normoxic	1.99E+04	2.15E+04	1.88E+04	2.23E+04	1.69E+04	1.63E+04	1.70E+04	2.58E+04	1.80E+04	4.21E+04	5.45E+04	3.91E+04	6.39E+04	5.41E+04	6.78E+04	4.98E+05	2.01E+04	1.85E+04
VF Hypoxic	Isotype	4.82E+02	4.12E+02	6.01E+02	1.74E+03	1.49E+03	3.49E+03	1.73E+03	6.96E+03	5.05E+03	4.03E+03	9.81E+03	6.79E+03	2.12E+04	1.90E+04	1.84E+04	1.01E+05	4.98E+02	2.24E+03
VF Hypoxic	Isotype	1.06E+06	6.14E+05	1.03E+06	1.35E+06	1.07E+06	1.28E+06	1.75E+06	1.81E+06	1.68E+06	1.95E+06	1.42E+06	1.87E+06	3.50E+06	3.39E+06	4.06E+06	2.78E+07	9.01E+05	1.24E+06
VF Hypoxic	Isotype	1.34E+03	2.10E+03	3.23E+03	4.41E+03	4.11E+03	6.35E+03	3.33E+03	9.45E+03	5.38E+03	7.01E+03	1.12E+04	6.14E+03	3.20E+04	2.82E+04	4.10E+04	1.65E+05	2.23E+03	4.96E+03
VF Hypoxic	Isotype	8.28E+02	2.13E+02	5.77E+02	1.46E+03	1.27E+03	8.02E+02	3.41E+03	2.08E+03	8.98E+02	2.70E+03	2.81E+03	2.23E+03	1.69E+04	1.80E+04	1.30E+04	6.72E+04	5.39E+02	1.18E+03
VF Normoxic	WT Normoxic	1.67E+02	7.27E+02	2.95E+02	1.46E+02	3.72E+02	4.94E+02	3.14E+02	4.11E+02	8.44E+02	8.29E+03	4.36E+03	5.42E+03	5.57E+03	6.48E+03	2.74E+03	3.66E+04	3.96E+02	3.37E+02
VF Hypoxic	Isotype	2.51E+05	2.23E+05	2.07E+05	1.78E+06	1.88E+06	5.82E+05	9.76E+05	3.04E+06	6.58E+05	3.86E+06	4.16E+06	4.80E+06	5.47E+06	5.84E+06	4.15E+06	3.79E+07	2.27E+05	1.41E+06
VF Hypoxic	Isotype	1.86E+04	3.04E+04	3.97E+04	4.78E+04	4.93E+04	5.17E+04	6.90E+04	1.83E+05	9.15E+04	1.26E+05	2.41E+05	1.24E+05	2.94E+05	3.03E+05	2.09E+05	1.88E+06	2.96E+04	4.96E+04
VF Hypoxic	Isotype	0.00E+00	4.68E+01	6.92E+01	2.57E+04	2.36E+04	1.07E+04	2.23E+04	1.41E+04	1.70E+04	2.31E+04	2.06E+04	1.03E+04	2.61E+04	2.35E+04	1.66E+04	2.34E+05	3.86E+01	2.00E+04
VF Hypoxic	Isotype	8.82E+03	1.09E+04	1.85E+04	1.64E+04	1.46E+04	2.11E+04	2.05E+04	3.28E+04	2.10E+04	3.37E+04	5.28E+04	3.70E+04	6.24E+04	5.50E+04	7.01E+04	4.76E+05	1.28E+04	1.73E+04
WT Normoxic	Isotype	1.50E+02	3.18E+02	1.52E+03	1.17E+04	1.82E+04	1.80E+04	6.60E+03	7.39E+03	8.69E+03	6.13E+03	8.39E+03	1.19E+04	7.33E+03	1.26E+04	1.17E+04	1.31E+05	6.62E+02	1.59E+04
VF Hypoxic	Isotype	2.29E+04	3.88E+04	3.95E+04	6.05E+04	5.26E+04	6.59E+04	5.56E+04	1.57E+05	5.21E+04	1.79E+05	2.37E+05	1.75E+05	2.48E+05	2.31E+05	2.05E+05	1.82E+06	3.37E+04	5.97E+04
VF Normoxic	Isotype	1.00E+03	1.71E+03	2.12E+03	2.03E+03	2.93E+03	4.30E+03	2.61E+03	8.41E+03	1.46E+03	2.24E+04	3.58E+04	1.69E+04	3.21E+04	2.44E+04	1.45E+04	1.73E+05	1.61E+03	3.09E+03
WT Normoxic	Isotype	5.36E+00	1.68E+02	0.00E+00	1.23E+04	8.39E+03	7.70E+03	1.12E+04	7.77E+03	8.91E+03	8.35E+03	8.02E+03	3.97E+03	1.13E+04	6.92E+03	6.32E+03	1.01E+05	5.79E+01	9.47E+03
VF Normoxic	WT Hypoxic	6.40E+04	7.71E+04	8.74E+04	4.83E+04	4.13E+04	7.86E+04	4.61E+04	5.15E+04	5.15E+04	1.45E+05	1.54E+05	2.07E+05	1.12E+05	1.09E+05	1.85E+05	1.46E+06	7.62E+04	5.61E+04
VF Hypoxic	Isotype	1.22E+04	2.72E+04	3.00E+04	4.33E+04	3.31E+04	4.40E+04	2.55E+04	1.10E+05	3.31E+04	1.33E+05	2.97E+05	1.31E+05	2.78E+05	2.78E+05	2.61E+05	1.74E+06	2.31E+04	4.02E+04
VF Hypoxic	Isotype	3.29E+01	1.67E+02	4.42E+02	2.77E+02	6.31E+02	1.01E+03	9.52E+02	3.12E+03	1.89E+02	1.06E+04	1.40E+04	1.02E+04	1.50E+04	1.16E+04	9.38E+03	7.75E+04	2.14E+02	6.41E+02
VF Hypoxic	Isotype	1.24E+04	1.42E+04	2.24E+04	1.37E+04	2.00E+04	2.46E+04	1.98E+04	2.70E+04	1.75E+04	3.32E+04	5.62E+04	3.47E+04	7.90E+04	5.80E+04	5.21E+04	4.85E+05	1.63E+04	1.94E+04
VF Normoxic	Isotype	3.96E+03	6.72E+03	5.44E+03	1.16E+04	1.11E+04	1.48E+04	1.03E+04	2.35E+04	1.10E+04	2.12E+04	4.48E+04	2.36E+04	3.65E+04	2.59E+04	2.19E+04	2.72E+05	5.37E+03	1.25E+04
VF Hypoxic	Isotype	8.49E+03	9.18E+03	9.74E+03	1.52E+04	9.71E+03	1.18E+04	1.24E+04	2.34E+04	1.09E+04	1.84E+04	2.60E+04	1.68E+04	3.53E+04	2.90E+04	3.82E+04	2.75E+05	9.13E+03	1.22E+04
VF Hypoxic	Isotype	2.75E+03	3.36E+03	6.23E+03	6.12E+03	3.84E+03	9.12E+03	5.29E+03	1.17E+04	4.44E+03	9.94E+03	1.85E+04	1.13E+04	3.80E+04	2.64E+04	2.74E+04	1.84E+05	4.11E+03	6.36E+03
VF Hypoxic	Isotype	1.70E+03	1.56E+03	2.78E+03	3.67E+03	1.85E+03	4.67E+03	2.09E+03	5.48E+03	1.37E+03	8.28E+03	1.85E+04	9.39E+03	1.78E+04	1.89E+04	1.17E+04	1.10E+05	2.01E+03	3.40E+03
VF Hypoxic	Isotype	1.70E+04	1.81E+04	1.37E+04	2.49E+04	2.06E+04	2.13E+04	2.34E+04	3.54E+04	1.94E+04	4.06E+04	4.55E+04	2.70E+04	4.34E+04	4.35E+04	5.74E+04	4.51E+05	1.63E+04	2.23E+04
VF Hypoxic	Isotype	7.02E+02	3.95E+03	9.98E+02	5.55E+03	2.49E+03	2.79E+03	2.96E+03	7.85E+03	2.35E+03	1.65E+04	4.95E+04	1.04E+04	3.05E+04	2.82E+04	2.07E+04	1.85E+05	1.88E+03	3.61E+03
VF Hypoxic	Isotype	4.93E+03	6.36E+03	5.03E+03	9.29E+03	7.81E+03	1.31E+04	1.47E+04	1.92E+04	1.11E+04	1.18E+04	3.24E+04	1.33E+04	2.30E+04	2.47E+04	3.31E+04	2.30E+05	5.44E+03	1.01E+04
VF Hypoxic	Isotype	5.24E+03	8.23E+03	8.33E+03	1.68E+04	1.13E+04	1.35E+04	1.24E+04	4.35E+04	7.06E+03	4.51E+04	6.50E+04	3.14E+04	7.18E+04	7.56E+04	5.01E+04	4.66E+05	7.27E+03	1.39E+04
VF Hypoxic	Isotype	8.24E+02	2.97E+03	3.90E+03	4.97E+03	3.67E+03	6.16E+03	4.67E+03	1.26E+04	4.64E+03	1.00E+04	1.87E+04	9.83E+03	2.59E+04	2.31E+04	2.18E+04	1.54E+05	2.56E+03	4.93E+03
VF Hypoxic	Isotype	6.48E+03	3.12E+03	9.61E+03	8.81E+03	8.04E+03	6.09E+03	8.97E+03	3.15E+04	9.53E+03	1.66E+04	3.13E+04	2.36E+04	5.21E+04	3.49E+04	4.83E+04	2.99E+05	6.40E+03	7.65E+03
VF Hypoxic	Isotype	1.57E+03	2.87E+03	2.39E+03	2.06E+03	1.93E+03	3.99E+03	2.77E+03	6.40E+03	3.18E+03	3.93E+03	6.24E+03	6.05E+03	9.50E+03	9.39E+03	9.67E+03	7.20E+04	2.28E+03	2.66E+03
VF Hypoxic	Isotype	5.53E+04	6.13E+04	5.24E+04	7.50E+04	6.05E+04	6.86E+04	6.90E+04	9.40E+04	6.53E+04	8.10E+04	9.87E+04	7.86E+04	1.20E+05	1.01E+05	1.71E+05	1.25E+06	5.63E+04	6.80E+04
VF Hypoxic	Isotype	2.91E+02	1.07E+03	1.75E+03	1.66E+03	1.21E+03	2.48E+03	2.14E+03	4.90E+03	1.63E+03	4.77E+03	1.00E+04	3.59E+03	1.16E+04	1.06E+04	1.05E+04	6.82E+04	1.04E+03	1.79E+03
WT Hypoxic	Isotype	2.54E+05	2.92E+05	2.94E+05	8.12E+05	4.24E+05	9.86E+05	8.10E+05	1.09E+06	7.89E+05	4.31E+05	4.54E+05	4.39E+05	4.97E+05	4.02E+05	6.95E+05	8.67E+06	2.80E+05	7.41E+05
VF Hypoxic	Isotype	2.44E+02	0.00E+00	8.22E+01	3.45E+02	4.40E+02	4.51E+02	1.18E+03	9.44E+03	1.82E+03	4.16E+03	1.04E+04	7.23E+03	3.88E+04	3.50E+04	1.75E+04	1.27E+05	1.09E+02	4.12E+02
VF Hypoxic	Isotype	3.37E+03	7.69E+03	6.29E+03	7.40E+03	5.34E+03	8.64E+03	6.42E+03	1.79E+04	7.68E+03	1.28E+04	2.70E+04	1.46E+04	2.42E+04	2.90E+04	2.90E+04	2.07E+05	5.78E+03	7.13E+03
VF Hypoxic	Isotype	4.39E+02	4.55E+02	1.81E+03	2.09E+03	1.41E+03	5.49E+03	1.70E+03	7.87E+03	3.38E+03	3.86E+03	1.69E+04	6.92E+03	1.56E+04	1.38E+04	2.00E+04	1.02E+05	9.03E+02	3.00E+03
VF Normoxic	Isotype	1.86E+02	1.35E+02	0.00E+00	7.52E+03	7.10E+03	1.05E+04	6.57E+03	7.26E+03	1.04E+04	1.13E+04	1.21E+04	4.89E+03	1.04E+04	7.97E+03	5.73E+03	1.02E+05	1.07E+02	8.38E+03
VF Hypoxic	WT Normoxic	1.51E+04	2.37E+04	2.00E+04	1.45E+04	8.75E+03	2.34E+04	1.43E+04	3.04E+04	1.35E+04	4.77E+04	5.33E+04	2.92E+04	6.58E+04	4.98E+04	6.28E+04	4.72E+05	1.96E+04	1.56E+04
VF Hypoxic	WT Normoxic	2.46E+04	3.14E+04	4.28E+04	3.31E+04	2.32E+04	3.18E+04	2.04E+04	5.41E+04	3.56E+04	4.62E+04	7.90E+04	5.14E+04	1.03E+05	9.05E+04	8.35E+04	7.50E+05	3.29E+04	2.94E+04
VF Hypoxic	Isotype	1.43E+03	3.79E+03	3.91E+03	4.75E+03	4.97E+03	6.76E+03	4.95E+03	1.59E+04	4.46E+03	9.99E+03	2.26E+04	8.75E+03	1.92E+04	2.30E+04	2.08E+04	1.55E+05	3.04E+03	5.49E+03
VF Hypoxic	WT Normoxic	2.23E+06	1.26E+06	8.38E+05	9.19E+05	5.55E+05	6.57E+05	6.88E+05	9.20E+05	6.94E+05	1.87E+06	2.10E+06	1.33E+06	1.93E+06	1.79E+06	1.66E+06	1.94E+07	1.44E+06	7.10E+05
VF Hypoxic	Isotype	3.72E+03	7.29E+03	9.38E+03	1.10E+04	8.05E+03	1.19E+04	7.38E+03	1.80E+04	1.05E+04	1.40E+04	2.43E+04	1.42E+04	4.03E+04	2.28E+04	2.73E+04	2.30E+05	6.80E+03	1.03E+04
VF Hypoxic	Isotype	1.49E+04	2.32E+04	2.10E+04	1.55E+04	2.45E+04	2.74E+04	1.78E+04	3.96E+04	9.61E+03	4.04E+04	4.58E+04	3.53E+04	1.04E+05	6.19E+04	7.14E+04	5.52E+05	1.97E+04	2.25E+04
VF Hypoxic	Isotype	6.80E+03	1.41E+04	1.16E+04	1.04E+04	1.14E+04	1.26E+04	9.23E+03	2.69E+04	9.85E+03	2.19E+04	3.18E+04	2.32E+04	2.92E+04	3.67E+04	4.61E+04	3.02E+05	1.08E+04	1.15E+04
VF Hypoxic	Isotype	1.16E+04	1.50E+04	2.20E+04	1.73E+04	1.91E+04	2.88E+04	2.33E+04	4.53E+04	2.06E+04	3.02E+04	3.78E+04	2.56E+04	8.52E+04	4.29E+04	6.16E+04	4.86E+05	1.62E+04	2.18E+04
VF Hypoxic	Isotype	4.34E+03	9.79E+03	1.89E+04	1.34E+04	1.03E+04	1.26E+04	1.17E+04	3.35E+04	1.26E+04	2.85E+04	4.17E+04	3.18E+04	4.43E+04	4.45E+04	5.76E+04	3.76E+05	1.10E+04	1.21E+04
VF Hypoxic	Isotype	2.48E+02	1.10E+03	1.83E+03	1.34E+03	5.36E+02	1.96E+03	1.75E+03	3.30E+03	1.48E+03	1.39E+03	4.00E+03	3.80E+03	1.35E+04	7.45E+03	1.04E+04	5.41E+04	1.06E+03	1.28E+03
VF Hypoxic	Isotype	2.57E+02	6.44E+02	1.88E+03	2.35E+03	2.18E+03	3.61E+03	1.60E+03	7.45E+03	2.40E+03	3.51E+03	8.70E+03	5.19E+03	7.98E+03	8.26E+03	6.87E+03	6.29E+04	9.27E+02	2.71E+03
VF Hypoxic	Isotype	1.26E+03	2.44E+03	6.26E+03	4.72E+03	3.04E+03	4.35E+03	4.47E+03	9.56E+03	3.38E+03	5.62E+03	1.21E+04	7.02E+03	1.84E+04	1.67E+04	1.80E+04	1.17E+05	3.32E+03	4.03E+03
VF Hypoxic	Isotype	5.72E+04	6.41E+04	9.46E+04	8.58E+04	4.36E+04	1.17E+05	6.23E+04	1.96E+05	9.32E+04	1.41E+05	2.65E+05	1.46E+05	3.26E+05	2.74E+05	2.72E+05	2.24E+06	7.20E+04	8.22E+04
VF Normoxic	Isotype	1.68E+03	1.32E+03	3.40E+03	2.35E+03	3.02E+03	4.60E+03	2.93E+03	1.27E+04	1.94E+03	1.50E+04	2.70E+04	1.58E+04	2.03E+04	1.94E+04	4.86E+03	1.36E+05	2.13E+03	3.32E+03
VF Hypoxic	WT Normoxic	7.91E+03	1.01E+04	1.40E+04	8.87E+03	1.03E+04	1.27E+04	9.50E+03	2.79E+04	1.10E+04	1.79E+04	2.92E+04	1.67E+04	3.34E+04	2.52E+04	3.32E+04	2.68E+05	1.07E+04	1.06E+04
VF Hypoxic	Isotype	1.11E+02	2.25E+02	9.81E+02	8.05E+02	9.07E+02	1.86E+03	7.59E+02	3.78E+03	3.08E+03	1.94E+03	2.10E+03	1.16E+03	2.94E+03	3.85E+03	2.98E+03	2.75E+04	4.39E+02	1.19E+03
VF Hypoxic	Isotype	2.45E+03	3.35E+03	5.98E+03	3.16E+03	3.35E+03	7.62E+03	5.94E+03	2.89E+04	1.18E+03	1.45E+04	3.93E+04	1.71E+04	6.57E+04	3.97E+04	3.43E+04	2.73E+05	3.93E+03	4.71E+03
VF Hypoxic	Isotype	9.70E+03	1.17E+04	1.29E+04	1.28E+04	1.43E+04	1.93E+04	1.16E+04	2.52E+04	1.38E+04	1.73E+04	3.90E+04	1.72E+04	3.74E+04	2.51E+04	4.73E+04	3.15E+05	1.14E+04	1.55E+04
VF Normoxic	Isotype	1.81E+02	2.09E+02	1.38E+02	5.01E+02	2.51E+02	3.40E+02	2.14E+02	1.22E+03	2.00E+02	2.56E+03	3.77E+03	5.12E+02	1.61E+03	2.47E+03	6.44E+02	1.48E+04	1.76E+02	3.64E+02
VF Hypoxic	WT Normoxic	2.24E+04	2.45E+04	5.15E+04	2.38E+04	2.34E+04	4.09E+04	2.58E+04	6.95E+04	2.14E+04	5.94E+04	9.03E+04	6.45E+04	7.20E+04	6.66E+04	1.02E+05	7.58E+05	3.28E+04	2.94E+04
VF Hypoxic	Isotype	0.00E+00	0.00E+00	5.75E+02	6.29E+02	6.55E+02	6.91E+02	2.32E+03	8.50E+02	3.65E+02	2.01E+02	3.93E+02	1.42E+02	1.18E+04	3.39E+02	2.63E+03	2.16E+04	1.92E+02	6.58E+02
VF Hypoxic	VF Normoxic	1.31E+03	9.63E+02	2.66E+03	8.72E+02	5.98E+03	2.95E+03	2.01E+03	1.65E+04	5.74E+02	2.34E+03	0.00E+00	0.00E+00	1.22E+04	3.96E+03	6.83E+03	5.92E+04	1.64E+03	3.27E+03
VF Normoxic	Isotype	8.62E+02	2.00E+03	5.78E+03	2.25E+03	2.44E+03	1.37E+04	4.09E+03	2.03E+04	2.17E+03	1.22E+04	4.23E+04	1.03E+04	2.42E+04	2.31E+04	1.49E+04	1.81E+05	2.88E+03	6.12E+03
VF Normoxic	WT Normoxic	4.25E+05	6.07E+05	7.46E+05	5.18E+05	4.17E+05	7.11E+05	4.79E+05	1.04E+06	9.94E+05	1.08E+06	1.09E+06	9.14E+05	8.95E+05	8.42E+05	1.24E+06	1.20E+07	5.93E+05	5.49E+05
VF Hypoxic	Isotype	5.88E+02	8.57E+02	3.61E+03	1.62E+03	1.22E+03	4.53E+03	1.55E+03	4.77E+03	9.65E+02	3.40E+03	1.13E+04	3.42E+03	6.56E+03	7.93E+03	7.31E+03	5.96E+04	1.69E+03	2.45E+03
VF Hypoxic	Isotype	0.00E+00	6.08E+01	5.54E+02	1.78E+02	2.09E+02	2.66E+03	3.22E+02	1.13E+03	3.01E+02	8.99E+02	6.28E+03	7.34E+02	8.31E+03	3.56E+03	2.21E+03	2.74E+04	2.05E+02	1.02E+03
VF Hypoxic	WT Normoxic	1.77E+03	1.11E+04	2.43E+04	1.43E+03	2.06E+03	6.18E+03	2.51E+03	1.74E+04	8.94E+03	8.47E+03	1.97E+04	1.08E+04	3.65E+04	1.71E+04	4.37E+04	2.12E+05	1.24E+04	3.23E+03
WT Hypoxic	Isotype	7.79E+03	8.79E+03	4.92E+03	7.62E+03	7.91E+03	7.14E+03	1.02E+04	1.37E+04	1.13E+04	7.12E+03	1.47E+04	8.40E+03	1.32E+04	1.10E+04	1.06E+04	1.44E+05	7.17E+03	7.56E+03
VF Hypoxic	WT Normoxic	1.50E+03	3.81E+03	1.71E+04	1.28E+03	2.86E+03	2.51E+03	1.33E+03	1.72E+04	8.13E+03	6.17E+03	1.70E+04	2.07E+04	2.37E+04	1.66E+04	1.59E+04	1.56E+05	7.47E+03	2.21E+03
VF Hypoxic	Isotype	1.23E+05	1.09E+05	2.34E+04	9.85E+04	9.39E+04	8.61E+04	9.88E+04	1.34E+05	1.07E+05	1.66E+05	1.93E+05	1.30E+05	2.27E+05	1.70E+05	1.79E+05	1.94E+06	8.52E+04	9.29E+04
VF Hypoxic	WT Normoxic	0.00E+00	7.65E+02	5.06E+03	1.49E+02	0.00E+00	1.62E+02	1.79E+03	5.54E+03	2.44E+03	3.39E+02	3.23E+02	6.05E+02	1.25E+04	1.49E+04	9.19E+03	5.38E+04	1.94E+03	1.04E+02
VF Hypoxic	Isotype	4.16E+03	2.71E+04	8.38E+03	9.12E+03	4.86E+04	3.64E+04	3.02E+04	9.82E+04	1.00E+04	4.04E+04	3.99E+04	4.13E+04	9.61E+04	5.37E+04	6.08E+04	6.04E+05	1.32E+04	3.14E+04
VF Hypoxic	Isotype	1.78E+04	4.68E+04	7.51E+04	3.35E+04	4.13E+04	9.54E+04	3.83E+04	1.18E+05	1.17E+05	6.82E+04	1.59E+05	7.95E+04	1.46E+05	1.30E+05	1.32E+05	1.30E+06	4.66E+04	5.67E+04
VF Normoxic	WT Hypoxic	6.58E+05	1.28E+06	4.19E+05	3.50E+05	6.30E+05	1.23E+06	6.52E+05	5.97E+05	3.96E+05	9.20E+05	1.56E+06	1.50E+06	1.12E+06	1.14E+06	1.13E+06	1.36E+07	7.86E+05	7.36E+05
VF Normoxic	Isotype	1.18E+05	8.28E+04	2.50E+04	9.36E+04	5.53E+04	1.04E+05	1.18E+05	1.13E+05	9.62E+04	1.65E+05	1.55E+05	1.12E+05	1.33E+05	1.31E+05	1.45E+05	1.65E+06	7.52E+04	8.41E+04
WT Hypoxic	Isotype	4.83E+04	5.47E+04	3.44E+04	9.51E+04	2.07E+04	8.24E+04	8.66E+04	1.33E+05	1.21E+05	1.03E+05	9.39E+04	4.93E+04	9.47E+04	8.05E+04	1.07E+05	1.20E+06	4.58E+04	6.61E+04
VF Hypoxic	Isotype	7.60E+03	2.73E+04	3.67E+04	3.76E+04	1.98E+04	7.32E+04	2.43E+04	1.36E+05	1.10E+05	4.46E+04	1.59E+05	3.56E+04	9.43E+04	9.71E+04	8.49E+04	9.88E+05	2.38E+04	4.35E+04
VF Hypoxic	Isotype	2.29E+03	8.17E+03	1.01E+04	1.17E+04	5.20E+03	5.32E+03	6.78E+03	1.69E+04	6.07E+03	1.15E+04	1.27E+04	1.79E+04	2.33E+04	9.55E+03	2.06E+04	1.68E+05	6.84E+03	7.39E+03
VF Hypoxic	Isotype	2.90E+03	9.60E+03	1.27E+04	9.65E+03	5.20E+03	2.09E+04	5.80E+03	5.89E+04	3.31E+04	1.52E+04	5.22E+04	1.28E+04	4.02E+04	4.02E+04	2.55E+04	3.45E+05	8.41E+03	1.19E+04
WT Hypoxic	Isotype	1.14E+06	1.53E+06	2.43E+06	1.71E+06	1.93E+06	3.60E+06	2.06E+06	4.84E+06	3.80E+06	2.15E+06	4.79E+06	2.72E+06	2.48E+06	3.02E+06	3.64E+06	4.18E+07	1.70E+06	2.42E+06
WT Hypoxic	Isotype	1.85E+06	2.46E+06	4.61E+06	3.23E+06	3.54E+06	6.14E+06	3.40E+06	8.47E+06	7.90E+06	3.60E+06	6.71E+06	3.95E+06	4.56E+06	4.85E+06	5.89E+06	7.12E+07	2.97E+06	4.30E+06
WT Hypoxic	Isotype	3.30E+02	8.75E+02	0.00E+00	6.29E+02	7.45E+02	1.43E+03	6.01E+02	5.56E+03	2.06E+03	1.33E+03	3.73E+03	6.67E+02	1.51E+02	2.05E+03	1.41E+03	2.16E+04	4.02E+02	9.35E+02
VF Hypoxic	WT Normoxic	2.41E+04	4.38E+04	7.20E+04	8.10E+03	1.81E+04	1.24E+04	6.56E+03	6.36E+04	3.96E+04	1.53E+04	2.29E+04	4.18E+04	2.07E+05	4.25E+04	2.10E+04	6.38E+05	4.66E+04	1.29E+04
VF Hypoxic	WT Normoxic	1.75E+03	8.99E+02	1.58E+03	3.08E+02	1.89E+03	3.62E+02	2.61E+02	6.39E+03	6.93E+02	1.04E+03	2.78E+03	2.54E+03	3.73E+03	2.43E+03	1.91E+03	2.86E+04	1.41E+03	8.52E+02
VF Hypoxic	Isotype	0.00E+00	1.92E+03	1.66E+03	7.27E+02	0.00E+00	2.99E+03	1.85E+02	3.98E+03	0.00E+00	2.64E+03	3.77E+03	1.63E+03	9.01E+03	4.77E+03	6.24E+03	3.95E+04	1.20E+03	1.24E+03
WT Normoxic	Isotype	4.94E+03	3.44E+03	1.93E+04	1.03E+04	1.12E+04	7.96E+04	1.22E+04	1.40E+04	3.16E+04	8.45E+03	6.16E+04	5.93E+03	1.03E+04	1.44E+04	7.18E+03	2.94E+05	9.23E+03	3.37E+04
VF Normoxic	WT Hypoxic	1.19E+04	2.84E+04	3.44E+04	1.72E+04	1.69E+04	3.66E+04	1.96E+04	2.76E+04	2.11E+04	2.23E+04	3.01E+04	6.36E+04	2.06E+04	2.57E+04	2.86E+04	4.05E+05	2.49E+04	2.36E+04
Isotype	WT Normoxic	4.67E+04	1.22E+04	6.06E+04	6.60E+03	2.16E+04	6.82E+03	3.95E+03	8.54E+04	1.67E+04	8.12E+03	3.59E+04	4.42E+04	2.51E+04	1.22E+04	2.35E+04	4.09E+05	3.98E+04	1.17E+04
VF Normoxic	Isotype	4.66E+03	2.95E+03	7.43E+02	4.25E+03	1.85E+03	4.79E+03	6.68E+03	2.07E+04	5.30E+03	8.78E+02	4.55E+04	1.98E+04	5.86E+03	3.86E+04	7.21E+01	1.63E+05	2.78E+03	3.63E+03
VF Normoxic	WT Normoxic	3.14E+04	2.05E+04	6.43E+04	1.36E+04	4.77E+04	2.43E+04	1.17E+04	8.37E+04	3.33E+04	1.68E+04	4.71E+04	7.98E+04	4.67E+04	1.91E+04	4.20E+04	5.82E+05	3.88E+04	2.86E+04

Mean			Mean/Iso					t-test vs iso					Relative percent Normalised Abunadance									
WT_Hx	VF_Nx	VF_Hx	WT_Nx	WT_Hx	VF_Nx	VF_Hx	Max	WT_Nx	WT_Hx	VF_Nx	VF_Hx	Min	Isotype			WT Normoxic			WT Hypoxic			V
Column2	Column3	Column4	Column1 3	Column2 4	Column3 5	Column46	Column46	Column46	Column46	Column46	Column47	Column5	D799_1 - Rel%	D799_2 - Rel%	D799_3 - Rel%	D799_4 - Rel%	D799_5 - Rel%	D799_6 - Rel%	D799_7 - Rel%	D799_8 - Rel%	D799_9 - Rel%	D799_10 - Rel%
8.91E+04	2.62E+05	2.85E+05	8.42	9.59	28.20	30.65	30.65	0.008	0.015	0.008	0.003	3.24E-03	0%	0%	0%	4%	4%	3%	5%	4%	3%	12%
5.70E+05	1.93E+05	1.83E+06	3.53	27.28	9.23	87.68	87.68	0.036	0.011	0.000	0.001	2.81E-05	0%	0%	0%	1%	1%	1%	6%	8%	8%	2%
1.31E+06	2.18E+06	6.71E+06	4.53	15.22	25.44	78.10	78.10	0.000	0.006	0.091	0.026	4.08E-04	0%	0%	0%	1%	1%	1%	3%	4%	4%	4%
1.07E+06	1.74E+07	1.48E+07	0.10	0.11	1.84	1.57	1.84	0.036	0.037	0.023	0.072	2.34E-02	6%	6%	10%	1%	1%	1%	1%	1%	1%	11%
7.87E+03	3.48E+05	3.15E+05	4.50	5.48	242.56	219.41	242.56	0.048	0.079	0.042	0.102	4.16E-02	0%	0%	0%	0%	0%	0%	0%	1%	0%	16%
1.65E+04	8.67E+03	1.22E+04	62.60	51.52	27.05	38.15	62.60	0.038	0.008	0.010	0.017	8.29E-03	0%	0%	0%	14%	14%	7%	11%	8%	9%	6%
9.11E+03	6.48E+03	7.45E+04	1.17	3.02	2.15	24.73	24.73	0.556	0.128	0.032	0.001	1.06E-03	1%	1%	1%	1%	1%	1%	2%	5%	3%	2%
1.56E+05	8.74E+05	6.29E+05	9.78	9.53	53.27	38.34	53.27	0.059	0.117	0.018	0.010	9.54E-03	0%	0%	0%	4%	3%	2%	2%	5%	2%	16%
1.17E+05	4.38E+04	7.58E+04	4.94	5.17	1.94	3.36	5.17	0.007	0.011	0.072	0.020	6.96E-03	2%	2%	2%	11%	10%	9%	9%	12%	10%	3%
4.88E+04	1.34E+05	3.25E+05	1.25	2.19	6.00	14.59	14.59	0.350	0.131	0.049	0.000	4.65E-06	1%	1%	2%	1%	2%	2%	2%	4%	3%	5%
1.84E+04	2.37E+04	4.92E+04	1.78	2.45	3.16	6.57	6.57	0.029	0.023	0.001	0.007	1.33E-03	2%	2%	3%	4%	4%	4%	5%	7%	5%	7%
4.72E+04	6.51E+04	9.55E+04	3.50	3.36	4.64	6.80	6.80	0.007	0.023	0.017	0.032	7.04E-03	2%	1%	2%	7%	6%	5%	5%	5%	7%	8%
2.03E+04	4.52E+04	6.19E+04	0.92	1.01	2.25	3.09	3.09	0.505	0.950	0.031	0.008	7.71E-03	4%	4%	4%	4%	3%	3%	3%	5%	4%	8%
4.58E+03	6.88E+03	1.95E+04	4.49	9.18	13.80	39.20	39.20	0.109	0.116	0.062	0.002	1.86E-03	0%	0%	1%	2%	1%	3%	2%	7%	5%	4%
1.75E+06	1.75E+06	3.65E+06	1.37	1.94	1.94	4.05	4.05	0.132	0.023	0.019	0.001	7.34E-04	4%	2%	4%	5%	4%	5%	6%	6%	6%	7%
6.05E+03	8.12E+03	3.37E+04	2.23	2.72	3.65	15.16	15.16	0.040	0.159	0.051	0.013	1.27E-02	1%	1%	2%	3%	2%	4%	2%	6%	3%	4%
2.13E+03	2.58E+03	1.60E+04	2.19	3.95	4.78	29.59	29.59	0.074	0.153	0.001	0.009	1.24E-03	1%	0%	1%	2%	2%	1%	5%	3%	1%	4%
5.23E+02	6.02E+03	4.93E+03	0.85	1.32	15.19	12.43	15.19	0.783	0.620	0.038	0.054	3.85E-02	0%	2%	1%	0%	1%	1%	1%	1%	2%	23%
1.56E+06	4.27E+06	5.15E+06	6.23	6.86	18.82	22.70	22.70	0.104	0.217	0.005	0.011	4.57E-03	1%	1%	1%	5%	5%	2%	3%	8%	2%	10%
1.15E+05	1.64E+05	2.69E+05	1.68	3.88	5.55	9.09	9.09	0.077	0.131	0.070	0.013	1.26E-02	1%	2%	2%	3%	3%	3%	4%	10%	5%	7%
1.78E+04	1.80E+04	2.21E+04	517.87	460.43	466.33	571.27	571.27	0.051	0.018	0.045	0.016	1.60E-02	0%	0%	0%	11%	10%	5%	10%	6%	7%	10%
2.48E+04	4.12E+04	6.25E+04	1.36	1.94	3.23	4.90	4.90	0.276	0.079	0.024	0.001	1.30E-03	2%	2%	4%	3%	3%	4%	4%	7%	4%	7%
7.56E+03	8.80E+03	1.05E+04	24.08	11.42	13.30	15.91	24.08	0.016	0.001	0.033	0.020	1.23E-03	0%	0%	1%	9%	14%	14%	5%	6%	7%	5%
8.83E+04	1.97E+05	2.28E+05	1.77	2.62	5.84	6.76	6.76	0.022	0.253	0.010	0.001	1.27E-03	1%	2%	2%	3%	3%	4%	3%	9%	3%	10%
4.16E+03	2.50E+04	2.37E+04	1.92	2.58	15.53	14.70	15.53	0.141	0.357	0.053	0.049	4.92E-02	1%	1%	1%	1%	2%	2%	2%	5%	1%	13%
9.29E+03	6.78E+03	8.19E+03	163.46	160.36	117.07	141.46	163.46	0.022	0.011	0.041	0.036	1.14E-02	0%	0%	0%	12%	8%	8%	11%	8%	9%	8%
4.97E+04	1.69E+05	1.35E+05	0.74	0.65	2.22	1.78	2.22	0.221	0.051	0.030	0.133	2.96E-02	4%	5%	6%	3%	3%	5%	3%	4%	4%	10%
5.62E+04	1.87E+05	2.72E+05	1.74	2.43	8.10	11.77	11.77	0.071	0.345	0.095	0.000	6.51E-06	1%	2%	2%	2%	2%	3%	1%	6%	2%	8%
1.42E+03	1.16E+04	1.20E+04	2.99	6.64	54.16	55.97	55.97	0.175	0.302	0.011	0.018	1.06E-02	0%	0%	1%	0%	1%	1%	1%	4%	0%	14%
2.14E+04	4.14E+04	6.30E+04	1.19	1.31	2.53	3.86	3.86	0.521	0.292	0.061	0.019	1.89E-02	3%	3%	5%	3%	4%	5%	4%	6%	4%	7%
1.49E+04	2.99E+04	2.81E+04	2.32	2.78	5.56	5.22	5.56	0.009	0.151	0.081	0.031	9.33E-03	1%	2%	2%	4%	4%	5%	4%	9%	4%	8%
1.56E+04	2.04E+04	3.42E+04	1.34	1.71	2.24	3.74	3.74	0.186	0.243	0.056	0.010	1.04E-02	3%	3%	4%	6%	4%	4%	5%	9%	4%	7%
7.13E+03	1.32E+04	3.06E+04	1.55	1.73	3.21	7.43	7.43	0.303	0.321	0.059	0.014	1.36E-02	1%	2%	3%	3%	2%	5%	3%	6%	2%	5%
2.98E+03	1.20E+04	1.62E+04	1.69	1.48	5.98	8.03	8.03	0.232	0.531	0.088	0.022	2.16E-02	2%	1%	3%	3%	2%	4%	2%	5%	1%	8%
2.61E+04	3.77E+04	4.81E+04	1.37	1.60	2.32	2.96	2.96	0.033	0.172	0.054	0.015	1.52E-02	4%	4%	3%	6%	5%	5%	5%	8%	4%	9%
4.39E+03	2.55E+04	2.65E+04	1.92	2.33	13.52	14.06	14.06	0.293	0.298	0.191	0.008	8.22E-03	0%	2%	1%	3%	1%	2%	2%	4%	1%	9%
1.50E+04	1.92E+04	2.69E+04	1.85	2.76	3.52	4.95	4.95	0.089	0.050	0.174	0.019	1.89E-02	2%	3%	2%	4%	3%	6%	6%	8%	5%	5%
2.10E+04	4.72E+04	6.59E+04	1.91	2.89	6.49	9.06	9.06	0.033	0.350	0.053	0.017	1.66E-02	1%	2%	2%	4%	2%	3%	3%	9%	2%	10%
7.30E+03	1.29E+04	2.36E+04	1.92	2.85	5.02	9.20	9.20	0.114	0.208	0.061	0.000	2.45E-04	1%	2%	3%	3%	2%	4%	3%	8%	3%	7%
1.67E+04	2.38E+04	4.51E+04	1.19	2.60	3.72	7.04	7.04	0.590	0.299	0.038	0.011	1.06E-02	2%	1%	3%	3%	3%	2%	3%	11%	3%	6%
4.12E+03	5.41E+03	9.52E+03	1.17	1.81	2.38	4.18	4.18	0.647	0.245	0.033	0.002	1.88E-03	2%	4%	3%	3%	3%	6%	4%	9%	4%	5%
7.61E+04	8.61E+04	1.31E+05	1.21	1.35	1.53	2.32	2.32	0.090	0.152	0.029	0.068	2.88E-02	4%	5%	4%	6%	5%	5%	6%	8%	5%	6%
2.89E+03	6.13E+03	1.09E+04	1.72	2.79	5.91	10.49	10.49	0.257	0.202	0.118	0.000	7.03E-05	0%	2%	3%	2%	2%	4%	3%	7%	2%	7%
8.97E+05	4.41E+05	5.31E+05	2.65	3.21	1.58	1.90	3.21	0.108	0.023	0.002	0.097	1.68E-03	3%	3%	3%	9%	5%	11%	9%	13%	9%	5%
4.15E+03	7.26E+03	3.05E+04	3.79	38.15	66.81	280.04	280.04	0.035	0.267	0.058	0.044	3.45E-02	0%	0%	0%	0%	0%	0%	1%	7%	1%	3%
1.07E+04	1.82E+04	2.74E+04	1.23	1.84	3.14	4.73	4.73	0.450	0.310	0.100	0.001	5.90E-04	2%	4%	3%	4%	3%	4%	3%	9%	4%	6%
4.31E+03	9.23E+03	1.65E+04	3.32	4.78	10.22	18.27	18.27	0.234	0.200	0.167	0.010	1.02E-02	0%	0%	2%	2%	1%	5%	2%	8%	3%	4%
8.07E+03	9.44E+03	8.03E+03	78.37	75.51	88.30	75.13	88.30	0.016	0.021	0.055	0.028	1.62E-02	0%	0%	0%	7%	7%	10%	6%	7%	10%	11%
1.94E+04	4.34E+04	5.95E+04	0.79	0.99	2.21	3.03	3.03	0.468	0.979	0.069	0.006	5.66E-03	3%	5%	4%	3%	2%	5%	3%	6%	3%	10%
3.67E+04	5.89E+04	9.23E+04	0.89	1.11	1.79	2.80	2.80	0.600	0.756	0.109	0.002	1.58E-03	3%	4%	6%	4%	3%	4%	3%	7%	5%	6%
8.45E+03	1.38E+04	2.10E+04	1.81	2.78	4.53	6.91	6.91	0.079	0.284	0.132	0.000	3.55E-04	1%	2%	3%	3%	3%	4%	3%	10%	3%	6%
7.68E+05	1.77E+06	1.79E+06	0.49	0.53	1.22	1.24	1.24	0.212	0.240	0.541	0.491	2.12E-01	11%	6%	4%	5%	3%	3%	4%	5%	4%	10%
1.20E+04	1.75E+04	3.01E+04	1.52	1.76	2.58	4.43	4.43	0.164	0.242	0.069	0.037	3.67E-02	2%	3%	4%	5%	3%	5%	3%	8%	5%	6%
2.23E+04	4.05E+04	7.90E+04	1.14	1.13	2.06	4.01	4.01	0.560	0.800	0.007	0.038	6.62E-03	3%	4%	4%	3%	4%	5%	3%	7%	2%	7%
1.53E+04	2.56E+04	3.73E+04	1.06	1.42	2.37	3.45	3.45	0.795	0.526	0.021	0.019	1.94E-02	2%	5%	4%	3%	4%	4%	3%	9%	3%	7%
2.97E+04	3.12E+04	6.32E+04	1.35	1.84	1.93	3.91	3.91	0.301	0.219	0.034	0.054	3.41E-02	2%	3%	5%	4%	4%	6%	5%	9%	4%	6%
1.93E+04	3.40E+04	4.88E+04	1.10	1.75	3.09	4.43	4.43	0.820	0.388	0.017	0.004	3.51E-03	1%	3%	5%	4%	3%	3%	3%	9%	3%	8%
2.18E+03	3.06E+03	1.05E+04	1.21	2.05	2.89	9.87	9.87	0.741	0.203	0.124	0.027	2.69E-02	0%	2%	3%	2%	1%	4%	3%	6%	3%	3%
3.82E+03	5.80E+03	7.70E+03	2.93	4.12	6.26	8.31	8.31	0.055	0.251	0.074	0.001	5.21E-04	0%	1%	3%	4%	3%	6%	3%	12%	4%	6%
5.80E+03	8.26E+03	1.77E+04	1.22	1.75	2.49	5.34	5.34	0.689	0.367	0.123	0.006	6.19E-03	1%	2%	5%	4%	3%	4%	4%	8%	3%	5%
1.17E+05	1.84E+05	2.91E+05	1.14	1.63	2.55	4.04	4.04	0.701	0.381	0.100	0.001	1.04E-03	3%	3%	4%	4%	2%	5%	3%	9%	4%	6%
5.85E+03	1.93E+04	1.49E+04	1.56	2.74	9.03	6.96	9.03	0.269	0.392	0.045	0.124	4.47E-02	1%	1%	2%	2%	2%	3%	2%	9%	1%	11%
1.61E+04	2.13E+04	3.06E+04	0.99	1.51	1.99	2.87	2.87	0.974	0.458	0.101	0.005	5.47E-03	3%	4%	5%	3%	4%	5%	4%	10%	4%	7%
2.54E+03	1.73E+03	3.26E+03	2.71	5.78	3.94	7.41	7.41	0.160	0.139	0.032	0.002	2.27E-03	0%	1%	4%	3%	3%	7%	3%	14%	11%	7%
1.20E+04	2.37E+04	4.66E+04	1.20	3.06	6.03	11.86	11.86	0.688	0.445	0.127	0.046	4.63E-02	1%	1%	2%	1%	1%	3%	2%	11%	0%	5%
1.69E+04	2.45E+04	3.66E+04	1.35	1.48	2.14	3.20	3.20	0.162	0.325	0.213	0.056	5.64E-02	3%	4%	4%	4%	5%	6%	4%	8%	4%	5%
5.44E+02	2.28E+03	1.57E+03	2.07	3.09	12.96	8.94	12.96	0.115	0.389	0.157	0.118	1.15E-01	1%	1%	1%	3%	2%	2%	1%	8%	1%	17%
3.89E+04	7.14E+04	8.01E+04	0.90	1.19	2.18	2.44	2.44	0.774	0.755	0.045	0.031	3.15E-02	3%	3%	7%	3%	3%	5%	3%	9%	3%	8%
1.18E+03	2.45E+02	4.92E+03	3.44	6.16	1.28	25.65	25.65	0.134	0.230	0.813	0.309	1.34E-01	0%	0%	3%	3%	3%	3%	11%	4%	2%	1%
6.35E+03	7.80E+02	7.68E+03	1.99	3.86	0.47	4.67	4.67	0.391	0.452	0.416	0.125	1.25E-01	2%	2%	4%	1%	10%	5%	3%	28%	1%	4%
8.84E+03	2.16E+04	2.07E+04	2.13	3.07	7.50	7.21	7.50	0.491	0.410	0.211	0.013	1.27E-02	0%	1%	3%	1%	1%	8%	2%	11%	1%	7%
8.39E+05	1.03E+06	9.94E+05	0.93	1.42	1.74	1.68	1.74	0.746	0.312	0.023	0.067	2.28E-02	4%	5%	6%	4%	3%	6%	4%	9%	8%	9%
2.43E+03	6.03E+03	7.27E+03	1.46	1.44	3.58	4.31	4.31	0.618	0.653	0.234	0.017	1.74E-02	1%	1%	6%	3%	2%	8%	3%	8%	2%	6%
5.84E+02	2.64E+03	4.69E+03	4.96	2.85	12.88	22.90	22.90	0.429	0.317	0.313	0.135	1.35E-01	0%	0%	2%	1%	1%	10%	1%	4%	1%	3%
9.63E+03	1.30E+04	3.24E+04	0.26	0.78	1.05	2.62	2.62	0.294	0.745	0.940	0.126	1.26E-01	1%	5%	11%	1%	1%	3%	1%	8%	4%	4%
1.17E+04	1.01E+04	1.16E+04	1.05	1.64	1.40	1.62	1.64	0.771	0.044	0.348	0.041	4.09E-02	5%	6%	3%	5%	5%	5%	7%	10%	8%	5%
8.87E+03	1.46E+04	1.87E+04	0.30	1.19	1.96	2.51	2.51	0.393	0.844	0.334	0.131	1.31E-01	1%	2%	11%	1%	2%	2%	1%	11%	5%	4%
1.13E+05	1.63E+05	1.92E+05	1.09	1.33	1.92	2.25	2.25	0.829	0.466	0.113	0.055	5.51E-02	6%	6%	1%	5%	5%	4%	5%	7%	6%	9%
3.26E+03	4.23E+02	1.22E+04	0.05	1.68	0.22	6.28	6.28	0.364	0.541	0.437	0.011	1.11E-02	0%	1%	9%	0%	0%	0%	3%	10%	5%	1%
4.62E+04	4.05E+04	7.02E+04	2.37	3.49	3.07	5.31	5.31	0.268	0.342	0.060	0.030	3.01E-02	1%	4%	1%	2%	8%	6%	5%	16%	2%	7%
9.11E+04	1.02E+05	1.36E+05	1.22	1.96	2.20	2.92	2.92	0.712	0.239	0.185	0.025	2.47E-02	1%	4%	6%	3%	3%	7%	3%	9%	9%	5%
5.49E+05	1.33E+06	1.13E+06	0.94	0.70	1.69	1.44	1.69	0.898	0.457	0.178	0.310	1.78E-01	5%	9%	3%	3%	5%	9%	5%	4%	3%	7%
1.09E+05	1.44E+05	1.36E+05	1.12	1.45	1.91	1.81	1.91	0.791	0.337	0.110	0.149	1.10E-01	7%	5%	2%	6%	3%	6%	7%	7%	6%	10%
1.14E+05	8.20E+04	9.39E+04	1.44	2.48	1.79	2.05	2.48	0.473	0.026	0.150	0.009	8.53E-03	4%	5%	3%	8%	2%	7%	7%	11%	10%	9%
8.99E+04	7.98E+04	9.21E+04	1.83	3.77	3.35	3.86	3.86	0.349	0.183	0.293	0.007	7.24E-03	1%	3%	4%	4%	2%	7%	2%	14%	11%	5%
9.92E+03	1.40E+04	1.78E+04	1.08	1.45	2.05	2.61	2.61	0.870	0.511	0.080	0.104	8.00E-02	1%	5%	6%	7%	3%	3%	4%	10%	4%	7%
3.26E+04	2.67E+04	3.53E+04	1.42	3.88	3.18	4.20	4.20	0.565	0.253	0.286	0.015	1.50E-02	1%	3%	4%	3%	2%	6%	2%	17%	10%	4%
3.56E+06	3.22E+06	3.04E+06	1.42	2.10	1.89	1.79	2.10	0.381	0.134	0.189	0.059	5.86E-02	3%	4%	6%	4%	5%	9%	5%	12%	9%	5%
6.59E+06	4.75E+06	5.10E+06	1.45	2.22	1.60	1.72	2.22	0.346	0.139	0.242	0.110	1.10E-01	3%	3%	6%	5%	5%	9%	5%	12%	11%	5%
2.74E+03	1.91E+03	1.20E+03	2.33	6.83	4.75	2.99	6.83	0.210	0.251	0.243	0.288	2.10E-01	2%	4%	0%	3%	3%	7%	3%	26%	10%	6%
3.66E+04	2.66E+04	9.01E+04	0.28	0.78	0.57	1.93	1.93	0.130	0.667	0.294	0.539	1.30E-01	4%	7%	11%	1%	3%	2%	1%	10%	6%	2%
2.45E+03	2.12E+03	2.69E+03	0.60	1.74	1.50	1.91	1.91	0.408	0.652	0.328	0.127	1.27E-01	6%	3%	6%	1%	7%	1%	1%	22%	2%	4%
1.39E+03	2.68E+03	6.67E+03	1.04	1.16	2.24	5.58	5.58	0.969	0.902	0.161	0.031	3.07E-02	0%	5%	4%	2%	0%	8%	0%	10%	0%	7%
1.93E+04	2.53E+04	1.06E+04	3.65	2.09	2.75	1.15	3.65	0.399	0.281	0.472	0.819	2.81E-01	2%	1%	7%	4%	4%	27%	4%	5%	11%	3%
2.28E+04	3.87E+04	2.50E+04	0.95	0.92	1.56	1.00	1.56	0.896	0.792	0.405	0.991	4.05E-01	3%	7%	8%	4%	4%	9%	5%	7%	5%	6%
3.53E+04	2.94E+04	2.02E+04	0.29	0.89	0.74	0.51	0.89	0.181	0.887	0.596	0.305	1.81E-01	11%	3%	15%	2%	5%	2%	1%	21%	4%	2%
1.09E+04	2.21E+04	1.48E+04	1.30	3.91	7.92	5.32	7.92	0.592	0.238	0.274	0.421	2.38E-01	3%	2%	0%	3%	1%	3%	4%	13%	3%	1%
4.29E+04	4.79E+04	3.59E+04	0.74	1.11	1.24	0.93	1.24	0.574	0.879	0.706	0.867	5.74E-01	5%	4%	11%	2%	8%	4%	2%	14%	6%	3%

					HiF Normalised Peak Area										
F Normoxic		VF Hypoxic			WT Normoxic			WT Hypoxic			VF Normoxic			VF Hypoxic	
D799_11 - Rel%	D799_12 - Rel%	D799_13 - Rel%	D799_14 - Rel%	D799_15 - Rel%	D799_4	D799_5	D799_6	D799_7	D799_8	D799_9	D799_10	D799_11	D799_12	D799_13	D799_14
14%	10%	13%	14%	12%	0.227	0.206	0.172	0.094	0.066	0.050	0.184	0.189	0.063	0.052	0.054
3%	2%	23%	21%	24%	0.136	0.231	0.197	0.405	0.445	0.452	0.130	0.126	0.054	0.340	0.295
5%	11%	17%	18%	28%	1.000	1.000	1.000	1.000	1.000	1.000	1.000	1.000	1.000	1.000	1.000
15%	14%	10%	10%	13%	2.277	1.933	3.176	0.911	1.023	0.553	10.332	12.009	5.166	2.428	2.331
24%	12%	17%	24%	5%	0.014	0.012	0.024	0.005	0.008	0.005	0.229	0.303	0.067	0.065	0.082
5%	4%	9%	7%	6%	0.063	0.060	0.032	0.017	0.010	0.011	0.007	0.006	0.002	0.003	0.002
2%	2%	26%	27%	24%	0.011	0.007	0.010	0.005	0.010	0.005	0.004	0.004	0.002	0.014	0.014
20%	12%	13%	13%	9%	0.576	0.430	0.236	0.086	0.188	0.079	0.611	0.675	0.191	0.127	0.118
5%	3%	8%	5%	8%	0.341	0.259	0.265	0.091	0.098	0.079	0.027	0.035	0.010	0.015	0.010
9%	10%	19%	20%	19%	0.060	0.062	0.095	0.028	0.050	0.032	0.059	0.091	0.049	0.058	0.057
8%	6%	16%	12%	16%	0.039	0.033	0.031	0.015	0.016	0.011	0.017	0.016	0.006	0.010	0.007
10%	6%	13%	14%	8%	0.145	0.125	0.109	0.038	0.029	0.041	0.046	0.048	0.015	0.020	0.019
11%	8%	13%	11%	14%	0.060	0.041	0.043	0.015	0.019	0.013	0.030	0.034	0.011	0.012	0.009
10%	7%	21%	19%	18%	0.005	0.004	0.009	0.002	0.005	0.004	0.003	0.006	0.002	0.004	0.003
5%	7%	13%	12%	15%	3.609	2.618	3.355	1.573	1.297	1.187	1.379	0.886	0.528	0.643	0.583
7%	4%	19%	17%	25%	0.012	0.010	0.017	0.003	0.007	0.004	0.005	0.007	0.002	0.006	0.005
4%	3%	25%	27%	19%	0.004	0.003	0.002	0.003	0.001	0.001	0.002	0.002	0.001	0.003	0.003
12%	15%	15%	18%	7%	0.000	0.001	0.001	0.000	0.000	0.001	0.006	0.003	0.002	0.001	0.001
11%	13%	14%	15%	11%	4.767	4.585	1.521	0.878	2.185	0.464	2.735	2.592	1.356	1.007	1.002
13%	7%	16%	16%	11%	0.128	0.121	0.135	0.062	0.132	0.065	0.089	0.150	0.035	0.054	0.052
9%	4%	11%	10%	7%	0.069	0.058	0.028	0.020	0.010	0.012	0.016	0.013	0.003	0.005	0.004
11%	8%	13%	12%	15%	0.044	0.036	0.055	0.018	0.024	0.015	0.024	0.033	0.010	0.011	0.009
6%	9%	6%	10%	9%	0.031	0.045	0.047	0.006	0.005	0.006	0.004	0.005	0.003	0.001	0.002
13%	10%	14%	13%	11%	0.162	0.129	0.172	0.050	0.113	0.037	0.126	0.148	0.050	0.046	0.040
21%	10%	19%	14%	8%	0.005	0.007	0.011	0.002	0.006	0.001	0.016	0.022	0.005	0.006	0.004
8%	4%	11%	7%	6%	0.033	0.021	0.020	0.010	0.006	0.006	0.006	0.005	0.001	0.002	0.001
11%	14%	8%	7%	13%	0.129	0.101	0.205	0.041	0.037	0.036	0.103	0.096	0.058	0.021	0.019
17%	8%	16%	16%	15%	0.116	0.081	0.115	0.023	0.079	0.023	0.094	0.185	0.037	0.051	0.048
18%	13%	19%	15%	12%	0.001	0.002	0.003	0.001	0.002	0.000	0.008	0.009	0.003	0.003	0.002
12%	7%	16%	12%	11%	0.037	0.049	0.064	0.018	0.019	0.012	0.024	0.035	0.010	0.015	0.010
16%	9%	13%	10%	8%	0.031	0.027	0.039	0.009	0.017	0.008	0.015	0.028	0.007	0.007	0.004
9%	6%	13%	11%	14%	0.041	0.024	0.031	0.011	0.017	0.008	0.013	0.016	0.005	0.006	0.005
10%	6%	21%	14%	15%	0.016	0.009	0.024	0.005	0.008	0.003	0.007	0.012	0.003	0.007	0.005
17%	9%	16%	17%	11%	0.010	0.005	0.012	0.002	0.004	0.001	0.006	0.012	0.003	0.003	0.003
10%	6%	10%	10%	13%	0.067	0.050	0.056	0.021	0.025	0.014	0.029	0.028	0.008	0.008	0.007
27%	6%	16%	15%	11%	0.015	0.006	0.007	0.003	0.006	0.002	0.012	0.031	0.003	0.006	0.005
14%	6%	10%	11%	14%	0.025	0.019	0.034	0.013	0.014	0.008	0.008	0.020	0.004	0.004	0.004
14%	7%	15%	16%	11%	0.045	0.028	0.035	0.011	0.031	0.005	0.032	0.041	0.009	0.013	0.013
12%	6%	17%	15%	14%	0.013	0.009	0.016	0.004	0.009	0.003	0.007	0.012	0.003	0.005	0.004
10%	8%	17%	12%	16%	0.024	0.020	0.016	0.008	0.023	0.007	0.012	0.019	0.007	0.010	0.006
9%	8%	13%	13%	13%	0.006	0.005	0.010	0.002	0.005	0.002	0.003	0.004	0.002	0.002	0.002
8%	6%	10%	8%	14%	0.200	0.148	0.179	0.062	0.068	0.046	0.057	0.062	0.022	0.022	0.017
15%	5%	17%	15%	15%	0.004	0.003	0.006	0.002	0.004	0.001	0.003	0.006	0.001	0.002	0.002
5%	5%	6%	5%	8%	2.172	1.038	2.576	0.728	0.785	0.557	0.305	0.283	0.124	0.091	0.069
8%	6%	31%	28%	14%	0.001	0.001	0.001	0.001	0.007	0.001	0.003	0.006	0.002	0.007	0.006
13%	7%	12%	14%	14%	0.020	0.013	0.023	0.006	0.013	0.005	0.009	0.017	0.004	0.004	0.005
17%	7%	15%	14%	20%	0.006	0.003	0.014	0.002	0.006	0.002	0.003	0.011	0.002	0.003	0.002
12%	5%	10%	8%	6%	0.020	0.017	0.027	0.006	0.005	0.007	0.008	0.008	0.001	0.002	0.001
11%	6%	14%	11%	13%	0.039	0.021	0.061	0.013	0.022	0.010	0.034	0.033	0.008	0.012	0.009
11%	7%	14%	12%	11%	0.088	0.057	0.083	0.018	0.039	0.025	0.033	0.049	0.015	0.019	0.016
15%	6%	12%	15%	13%	0.013	0.012	0.018	0.004	0.011	0.003	0.007	0.014	0.002	0.004	0.004
11%	7%	10%	9%	9%	2.457	1.356	1.716	0.619	0.661	0.490	1.323	1.310	0.377	0.354	0.307
11%	6%	17%	10%	12%	0.029	0.020	0.031	0.007	0.013	0.007	0.010	0.015	0.004	0.007	0.004
8%	6%	19%	11%	13%	0.041	0.060	0.072	0.016	0.028	0.007	0.029	0.029	0.010	0.019	0.011
11%	8%	10%	12%	15%	0.028	0.028	0.033	0.008	0.019	0.007	0.016	0.020	0.007	0.005	0.006
8%	5%	18%	9%	13%	0.046	0.047	0.075	0.021	0.033	0.015	0.021	0.024	0.007	0.016	0.007
11%	8%	12%	12%	15%	0.036	0.025	0.033	0.010	0.024	0.009	0.020	0.026	0.009	0.008	0.008
7%	7%	25%	14%	19%	0.004	0.001	0.005	0.002	0.002	0.001	0.001	0.002	0.001	0.002	0.001
14%	8%	13%	13%	11%	0.006	0.005	0.009	0.001	0.005	0.002	0.002	0.005	0.001	0.001	0.001
10%	6%	16%	14%	15%	0.013	0.007	0.011	0.004	0.007	0.002	0.004	0.008	0.002	0.003	0.003
12%	7%	15%	12%	12%	0.229	0.107	0.306	0.056	0.141	0.066	0.100	0.165	0.041	0.060	0.047
20%	12%	15%	14%	4%	0.006	0.007	0.012	0.003	0.009	0.001	0.011	0.017	0.004	0.004	0.003
11%	6%	12%	9%	12%	0.024	0.025	0.033	0.009	0.020	0.008	0.013	0.018	0.005	0.006	0.004
8%	4%	11%	14%	11%	0.002	0.002	0.005	0.001	0.003	0.002	0.001	0.001	0.000	0.001	0.001
14%	6%	24%	15%	13%	0.008	0.008	0.020	0.005	0.021	0.001	0.010	0.025	0.005	0.012	0.007
12%	5%	12%	8%	15%	0.034	0.035	0.050	0.010	0.018	0.010	0.012	0.024	0.005	0.007	0.004
25%	3%	11%	17%	4%	0.001	0.001	0.001	0.000	0.001	0.000	0.002	0.002	0.000	0.000	0.000
12%	9%	10%	9%	13%	0.064	0.057	0.107	0.023	0.050	0.015	0.042	0.056	0.018	0.013	0.011
2%	1%	55%	2%	12%	0.002	0.002	0.002	0.002	0.001	0.000	0.000	0.000	0.000	0.002	0.000
0%	0%	21%	7%	12%	0.002	0.015	0.008	0.002	0.012	0.000	0.002	0.000	0.000	0.002	0.001
23%	6%	13%	13%	8%	0.006	0.006	0.036	0.004	0.015	0.002	0.009	0.026	0.003	0.004	0.004
9%	8%	7%	7%	10%	1.385	1.019	1.857	0.431	0.750	0.701	0.767	0.680	0.258	0.165	0.145
19%	6%	11%	13%	12%	0.004	0.003	0.012	0.001	0.003	0.001	0.002	0.007	0.001	0.001	0.001
23%	3%	30%	13%	8%	0.000	0.001	0.007	0.000	0.001	0.000	0.001	0.004	0.000	0.002	0.001
9%	5%	17%	8%	21%	0.004	0.005	0.016	0.002	0.013	0.006	0.006	0.012	0.003	0.007	0.003
10%	6%	9%	8%	7%	0.020	0.019	0.019	0.009	0.010	0.008	0.005	0.009	0.002	0.002	0.002
11%	13%	15%	11%	10%	0.003	0.007	0.007	0.001	0.012	0.006	0.004	0.011	0.006	0.004	0.003
10%	7%	12%	9%	9%	0.263	0.230	0.225	0.089	0.097	0.076	0.118	0.121	0.037	0.042	0.029
1%	1%	23%	28%	17%	0.000	0.000	0.000	0.002	0.004	0.002	0.000	0.000	0.000	0.002	0.003
7%	7%	16%	9%	10%	0.024	0.119	0.095	0.027	0.071	0.007	0.029	0.025	0.012	0.018	0.009
12%	6%	11%	10%	10%	0.089	0.101	0.249	0.034	0.085	0.082	0.048	0.099	0.022	0.027	0.022
11%	11%	8%	8%	8%	0.935	1.540	3.207	0.587	0.429	0.279	0.652	0.972	0.424	0.207	0.196
9%	7%	8%	8%	9%	0.250	0.135	0.271	0.106	0.081	0.068	0.117	0.097	0.032	0.024	0.023
8%	4%	8%	7%	9%	0.254	0.051	0.215	0.078	0.095	0.085	0.073	0.059	0.014	0.017	0.014
16%	4%	10%	10%	9%	0.100	0.048	0.191	0.022	0.098	0.077	0.032	0.099	0.010	0.017	0.017
8%	11%	14%	6%	12%	0.031	0.013	0.014	0.006	0.012	0.004	0.008	0.008	0.005	0.004	0.002
15%	4%	12%	12%	7%	0.026	0.013	0.055	0.005	0.042	0.023	0.011	0.033	0.004	0.007	0.007
11%	7%	6%	7%	9%	4.579	4.715	9.418	1.852	3.475	2.679	1.524	2.987	0.770	0.456	0.519
9%	6%	6%	7%	8%	8.641	8.653	16.042	3.055	6.086	5.573	2.547	4.185	1.118	0.839	0.833
17%	3%	1%	9%	7%	0.002	0.002	0.004	0.001	0.004	0.001	0.001	0.002	0.000	0.000	0.000
4%	7%	32%	7%	3%	0.022	0.044	0.032	0.006	0.046	0.028	0.011	0.014	0.012	0.038	0.007
10%	9%	13%	9%	7%	0.001	0.005	0.001	0.000	0.005	0.000	0.001	0.002	0.001	0.001	0.000
10%	4%	23%	12%	16%	0.002	0.000	0.008	0.000	0.003	0.000	0.002	0.002	0.000	0.002	0.001
21%	2%	3%	5%	2%	0.028	0.027	0.208	0.011	0.010	0.022	0.006	0.038	0.002	0.002	0.002
7%	16%	5%	6%	7%	0.046	0.041	0.096	0.018	0.020	0.015	0.016	0.019	0.018	0.004	0.004
9%	11%	6%	3%	6%	0.018	0.053	0.018	0.004	0.061	0.012	0.006	0.022	0.012	0.005	0.002
28%	12%	4%	24%	0%	0.011	0.005	0.013	0.006	0.015	0.004	0.001	0.028	0.006	0.001	0.007
8%	14%	8%	3%	7%	0.036	0.117	0.063	0.010	0.060	0.024	0.012	0.029	0.023	0.009	0.003

	Mean Hif Normalised Peak Area				GeoMean				Vs Isotype	
	WT_Nx	WT_Hx	VF_Nx	VF_Hx	WT_Nx	WT_Hx	VF_Nx	VF_Hx	Max F.C vs Isotype	Min t.test p-value vs isotype
D799_15	Column1	Column2	Column3	Column4	Column1	Column2	Column3	Column4	Max F.C vs Isotype	Min t.test p-value vs isotype
0.029	0.201	0.070	0.145	0.045	0.200	0.068	0.130	0.043	30.65	0.003
0.218	0.188	0.434	0.103	0.284	0.184	0.433	0.096	0.280	87.68	0.000
1.000	1.000	1.000	1.000	1.000	1.000	1.000	1.000	1.000	78.10	0.000
1.984	2.462	0.829	9.169	2.248	2.409	0.802	8.622	2.239	1.84	0.023
0.012	0.017	0.006	0.200	0.053	0.016	0.006	0.167	0.041	242.56	0.042
0.001	0.052	0.013	0.005	0.002	0.049	0.013	0.004	0.002	62.60	0.008
0.008	0.009	0.007	0.003	0.012	0.009	0.007	0.003	0.011	24.73	0.001
0.057	0.414	0.118	0.492	0.101	0.388	0.109	0.429	0.095	53.27	0.010
0.009	0.288	0.089	0.024	0.012	0.286	0.089	0.021	0.011	5.17	0.007
0.037	0.072	0.037	0.066	0.051	0.070	0.036	0.064	0.050	14.59	0.000
0.006	0.034	0.014	0.013	0.008	0.034	0.014	0.012	0.007	6.57	0.001
0.007	0.127	0.036	0.036	0.016	0.126	0.036	0.032	0.014	6.80	0.007
0.008	0.048	0.016	0.025	0.010	0.047	0.015	0.022	0.009	3.09	0.008
0.002	0.006	0.003	0.004	0.003	0.005	0.003	0.003	0.003	39.20	0.002
0.458	3.194	1.352	0.931	0.561	3.165	1.343	0.864	0.556	4.05	0.001
0.005	0.013	0.005	0.005	0.005	0.013	0.004	0.004	0.005	15.16	0.013
0.001	0.003	0.002	0.001	0.003	0.003	0.001	0.001	0.002	29.59	0.001
0.000	0.001	0.000	0.003	0.001	0.001	0.000	0.003	0.001	15.19	0.038
0.469	3.624	1.176	2.228	0.826	3.215	0.962	2.126	0.779	22.70	0.005
0.024	0.128	0.086	0.092	0.043	0.128	0.081	0.078	0.040	9.09	0.013
0.002	0.051	0.014	0.011	0.004	0.048	0.013	0.008	0.003	571.27	0.016
0.008	0.045	0.019	0.022	0.010	0.044	0.019	0.020	0.010	4.90	0.001
0.001	0.041	0.006	0.004	0.002	0.040	0.006	0.004	0.002	24.08	0.001
0.023	0.154	0.067	0.108	0.036	0.153	0.059	0.097	0.035	6.76	0.001
0.002	0.008	0.003	0.014	0.004	0.008	0.002	0.012	0.003	15.53	0.049
0.001	0.025	0.007	0.004	0.001	0.024	0.007	0.003	0.001	163.46	0.011
0.021	0.145	0.038	0.086	0.020	0.139	0.038	0.083	0.020	2.22	0.030
0.029	0.104	0.042	0.106	0.043	0.103	0.035	0.087	0.042	11.77	0.000
0.001	0.002	0.001	0.006	0.002	0.001	0.001	0.006	0.002	55.97	0.011
0.006	0.050	0.017	0.023	0.010	0.049	0.016	0.020	0.009	3.86	0.019
0.002	0.032	0.011	0.017	0.005	0.032	0.011	0.014	0.004	5.56	0.009
0.004	0.032	0.012	0.011	0.005	0.031	0.011	0.010	0.005	3.74	0.010
0.003	0.017	0.005	0.007	0.005	0.015	0.005	0.006	0.005	7.43	0.014
0.001	0.009	0.002	0.007	0.003	0.008	0.002	0.006	0.002	8.03	0.022
0.006	0.057	0.020	0.022	0.007	0.057	0.019	0.018	0.007	2.96	0.015
0.002	0.009	0.003	0.015	0.004	0.009	0.003	0.010	0.004	14.06	0.008
0.004	0.026	0.012	0.011	0.004	0.025	0.011	0.009	0.004	4.95	0.019
0.006	0.036	0.016	0.027	0.011	0.035	0.012	0.023	0.010	9.06	0.017
0.002	0.013	0.006	0.007	0.004	0.012	0.005	0.006	0.004	9.20	0.000
0.005	0.020	0.012	0.013	0.007	0.019	0.011	0.012	0.007	7.04	0.011
0.001	0.007	0.003	0.003	0.001	0.006	0.003	0.003	0.001	4.18	0.002
0.019	0.176	0.059	0.047	0.020	0.174	0.058	0.043	0.020	2.32	0.029
0.001	0.005	0.002	0.004	0.002	0.004	0.002	0.003	0.002	10.49	0.000
0.078	1.929	0.690	0.237	0.080	1.797	0.683	0.220	0.079	3.21	0.002
0.002	0.001	0.003	0.004	0.005	0.001	0.002	0.003	0.004	280.04	0.035
0.003	0.018	0.008	0.010	0.004	0.018	0.007	0.009	0.004	4.73	0.001
0.002	0.008	0.003	0.005	0.003	0.007	0.003	0.004	0.002	18.27	0.010
0.001	0.022	0.006	0.006	0.001	0.021	0.006	0.004	0.001	88.30	0.016
0.007	0.040	0.015	0.025	0.009	0.037	0.014	0.021	0.009	3.03	0.006
0.009	0.076	0.027	0.032	0.015	0.075	0.026	0.029	0.014	2.80	0.002
0.002	0.014	0.006	0.008	0.003	0.014	0.005	0.006	0.003	6.91	0.000
0.188	1.843	0.590	1.003	0.283	1.788	0.585	0.868	0.273	1.24	0.212
0.003	0.027	0.009	0.010	0.005	0.026	0.009	0.008	0.004	4.43	0.037
0.008	0.058	0.017	0.022	0.013	0.056	0.015	0.020	0.012	4.01	0.007
0.005	0.030	0.012	0.014	0.006	0.029	0.010	0.013	0.006	3.45	0.019
0.007	0.056	0.023	0.017	0.010	0.055	0.021	0.015	0.009	3.91	0.034
0.007	0.031	0.014	0.018	0.007	0.031	0.013	0.017	0.007	4.43	0.004
0.001	0.003	0.002	0.002	0.002	0.003	0.002	0.001	0.002	9.87	0.027
0.001	0.007	0.003	0.003	0.001	0.007	0.002	0.003	0.001	8.31	0.001
0.002	0.010	0.004	0.005	0.003	0.010	0.004	0.004	0.003	5.34	0.006
0.031	0.214	0.087	0.102	0.046	0.196	0.080	0.088	0.044	4.04	0.001
0.001	0.009	0.004	0.011	0.003	0.008	0.003	0.009	0.002	9.03	0.045
0.004	0.027	0.012	0.012	0.005	0.027	0.011	0.010	0.005	2.87	0.005
0.000	0.003	0.002	0.001	0.001	0.003	0.002	0.001	0.000	7.41	0.002
0.004	0.012	0.009	0.013	0.008	0.011	0.005	0.011	0.007	11.86	0.046
0.005	0.040	0.013	0.014	0.006	0.039	0.012	0.011	0.005	3.20	0.056
0.000	0.001	0.000	0.001	0.000	0.001	0.000	0.001	0.000	12.96	0.115
0.011	0.076	0.029	0.039	0.012	0.073	0.026	0.035	0.012	2.44	0.031
0.000	0.002	0.001	0.000	0.001	0.002	0.001	0.000	0.000	25.65	0.134
0.001	0.008	0.005	0.001	0.001	0.006	0.002	0.000	0.001	4.67	0.125
0.002	0.016	0.007	0.013	0.003	0.011	0.004	0.009	0.003	7.50	0.013
0.140	1.421	0.627	0.568	0.150	1.379	0.610	0.513	0.150	1.74	0.023
0.001	0.006	0.002	0.003	0.001	0.005	0.001	0.003	0.001	4.31	0.017
0.000	0.003	0.000	0.002	0.001	0.001	0.000	0.001	0.001	22.90	0.135
0.005	0.008	0.007	0.007	0.005	0.007	0.006	0.006	0.005	2.62	0.126
0.001	0.019	0.009	0.006	0.002	0.019	0.009	0.005	0.002	1.64	0.041
0.002	0.006	0.006	0.007	0.003	0.005	0.004	0.006	0.003	2.51	0.131
0.020	0.239	0.087	0.092	0.030	0.239	0.087	0.081	0.029	2.25	0.055
0.001	0.000	0.002	0.000	0.002	0.000	0.002	0.000	0.002	6.28	0.011
0.007	0.079	0.035	0.022	0.011	0.065	0.024	0.020	0.010	5.31	0.030
0.015	0.147	0.067	0.057	0.021	0.131	0.062	0.048	0.021	2.92	0.025
0.127	1.894	0.432	0.682	0.177	1.665	0.413	0.645	0.173	1.69	0.178
0.016	0.219	0.085	0.082	0.021	0.209	0.084	0.071	0.021	1.91	0.110
0.012	0.173	0.086	0.048	0.014	0.140	0.086	0.039	0.014	2.48	0.009
0.010	0.113	0.066	0.047	0.015	0.098	0.055	0.032	0.014	3.86	0.007
0.002	0.019	0.008	0.007	0.003	0.018	0.007	0.007	0.003	2.61	0.080
0.003	0.031	0.024	0.016	0.006	0.026	0.017	0.011	0.005	4.20	0.015
0.410	6.237	2.669	1.761	0.462	5.880	2.583	1.519	0.460	2.10	0.059
0.665	11.112	4.905	2.616	0.779	10.625	4.697	2.284	0.775	2.22	0.110
0.000	0.002	0.002	0.001	0.000	0.002	0.001	0.001	0.000	6.83	0.210
0.002	0.033	0.027	0.012	0.016	0.031	0.020	0.012	0.009	1.93	0.130
0.000	0.002	0.002	0.001	0.000	0.002	0.001	0.001	0.000	1.91	0.127
0.001	0.003	0.001	0.002	0.001	0.000	0.000	0.001	0.001	5.58	0.031
0.001	0.088	0.014	0.015	0.002	0.054	0.013	0.007	0.002	3.65	0.281
0.003	0.061	0.017	0.018	0.004	0.057	0.017	0.017	0.004	1.56	0.405
0.003	0.029	0.026	0.014	0.003	0.026	0.014	0.012	0.003	0.89	0.181
0.000	0.009	0.008	0.012	0.003	0.009	0.007	0.005	0.000	7.92	0.238
0.005	0.072	0.031	0.021	0.006	0.065	0.025	0.020	0.005	1.24	0.574
