# Supplementary material for: JAK2V617F reprograms Hypoxia Inducible Factor-1 to induce a non-canonical hypoxia regulon in myeloproliferative neoplasms
Source: Leukemia. 2026 Feb 2;40(3):609–21. doi: 10.1038/s41375-025-02843-9 (PMC12960217; doi:10.1038/s41375-025-02843-9)
Supplement: Supplementary file 2 — Supplementary Table S2 [file 41375_2025_2843_MOESM2_ESM.pdf]

Table S2. 200-gene HIF-1a signatures derived from BaF3/hMPL ChIPseq.

WT_Hx		VF_Nx		VF_Hx	
Enriched cf. WT_Nx q<0.03		Enriched cf. WT_Hx q<0.03		Enriched cf. WT_Hx q<0.03	
range	best_feat	range	best_feat	range	best_feat
9:114618095-114618495	Cmtm8	11.121127373-121127773	Narf	11.117133450-117133850	Septin9
16:15766019-15766419	Spidr	16:70111208-70111608	Gbe1	16:37908039-37908439	Gsk3b
2.26461881-26462281	Egfl7	5:144941254-144941654	Kpna7	9:114618095-114618495	Cmtm8
14:67245599-67245999	Bnip3l	11.97206298-97206698	Mrpl45	11.6365377-6365777	Ppia
1.59951686-59952086	Fam117b	3.103821378-103821778	Phtf1	6:124806268-124806668	Usp5
3.108118122-108118522	Atxn7l2	15:80103457-80103857	Mgat3	6:124806268-124806668	Cdca3
11.98821822-98822222	Rara	10:79540003-79540403	Bsg	4:149529785-149530185	Rbp7
4:138064149-138064549	Cda	19:36386736-36387136	Pcgf5	3.105802482-105802882	Tmigd3
14:22069358-22069758	Lrmda	8:75810477-75810877	Gm2059	8:71849350-71849750	Babam1
2.14060891-14061291	Hacd1	11.44411057-44411457	Rnf145	13:93441305-93441705	Homer1
3:95125051-95125451	Gabpb2	17:56632366-56632766	Kdm4b	3.107539069-107539469	Strip1
19:5579666-5580066	Gm31166	6:82798796-82799196	Gm32591	5:136654459-136654859	Cux1
2:71283556-71283956	Metap1d	X:102660571-102660971	Ftx	6:82975435-82975835	M1ap
17:36132121-36132521	Ier3	6:29272355-29272755	Hilpda	5:140690188-140690588	Brat1
17:56891015-56891415	Safb2	8:96560213-96560613	Gm31805	2:79528982-79529382	Ppp1r1c
10:59800325-59800725	Ddit4	10:86541566-86541966	Hsp90b1	7:119392886-119393286	Eri2
11.79145384-79145784	Wsb1	10:86541566-86541966	Ttc41	7:119392886-119393286	Rexo5
4:99786815-99787215	Pgm1	9:123357237-123357637	Sacm1l	10:117546534-117546934	Mdm2
10:3923533-3923933	Mthfd1l	9:65735278-65735678	Zfp609	9:21996184-21996584	Ecsit
1.170450454-170450854	Gm7299	11.116197513-116197913	Exoc7	7:113891715-113892115	Psma1
17:28527327-28527727	Fance	3:95789399-95789799	Ciart	4:138064159-138064559	Cda
4:45084727-45085127	Fbxo10	15:78831731-78832131	Triobp	15:99561929-99562329	Asic1
8:4727497-4727897	Gm7461	11.60427927-60428327	Alkbh5	4:99082091-99082491	Atg4c
15:6598688-6599088	Fyb	8:57941474-57941874	2500002B1	17:57338861-57339261	Khsrp
9:121686626-121687026	Higd1a	16:92349575-92349975	Gm49722	4:45084727-45085127	Fbxo10
13:37240627-37241027	F13a1	14:45555918-45556318	Ero1a	2.152631205-152631605	Bcl2l1
8:25591598-25591998	Plekha2	4:101277083-101277483	Ak4	14:71018394-71018794	Dok2
15:101043333-101043733	Acvrl1	5:53746189-53746589	Rbpj	1.177810536-177810936	Catspere2
6:82751456-82751856	Hk2	7:89590372-89590772	Hikeshi	10:79764072-79764472	Arid3a
8:72120751-72121151	Unc13a	7:138511125-138511525	Bnip3	17:28527327-28527727	Fance
3:35807844-35808244	Atp11b	6:125143364-125143764	Gapdh	14:25607129-25607529	Zmiz1
16:35891806-35892206	Ccdc58	16:15964492-15964892	Spidr	8:3681063-3681463	Stxbp2
11.55004563-55004963	Gm2a	3:95558477-95558877	Mcl1	1.59951686-59952086	Fam117b
11.115902980-115903380	Galk1	13:59919109-59919509	Tut7	13:93328880-93329280	Tent2
1.128344365-128344765	Dars	7:113891714-113892114	Psma1	11.84806886-84807286	Znhit3
1.171238552-171238952	Usf1	11.33113247-33113647	Npm1	9:57817840-57818240	Ubl7
4:46389325-46389725	Trmo	3:40801399-40801799	Mfsd8	9:65735284-65735684	Zfp609
15:99561932-99562332	Asic1	4:132203635-132204035	Med18	5:31855647-31856047	Babam2
3:89337590-89337990	Pygo2	8:106293332-106293732	Agrp	10:86541566-86541966	Hsp90b1
2.119727704-119728104	Mga	2:73300591-73300991	Wipf1	10:86541566-86541966	Ttc41
8:27750967-27751367	Eif4ebp1	15:78152026-78152426	Ncf4	11.55004563-55004963	Gm2a
8:3725964-3726364	Trappc5	7:100020790-100021190	C2cd3	17:26999776-27000176	Bnip1
9:96360488-96360888	Rnf7	18:35631474-35631874	Gm50149	6:86770553-86770953	Anxa4
X:36455606-36456006	Rnf113a1	18:35631474-35631874	Sil1	17:27720528-27720928	Grm4
5:135197086-135197486	Bcl7b	13.107231014-107231414	Gm31452	10:62176028-62176428	Hk1
2.25114884-25115284	Tubb4b	8:34221854-34222254	Gtf2e2	8:71917118-71917518	Mrpl34
2.167209253-167209653	B4galt5	3.138148362-138148762	Adh5	18:49888444-49888844	Dtwd2
11.116733525-116733925	Jmjd6	11.52251473-52251873	Vdac1	17:29171154-29171554	Kctd20
11.116733525-116733925	Mettl23	11.109363734-109364134	Slc16a6	1.44158007-44158407	Bivm
2.125514736-125515136	Shc4	3.138781152-138781552	Rap1gds1	4:40722520-40722920	Dnaja1

3.107539069-107539469	Strip1	4:101150243-101150643	Gm12796	7:79116314-79116714	Polg
3.107539069-107539469	Gm10961	5:23637855-23638255	Kmt2e	19:6096789-6097189	Syvn1
1.190930222-190930622	Atf3	9:114223941-114224341	Crtap	8:3725964-3726364	Trappc5
17:36147782-36148182	Tubb5	17:33903539-33903939	Hnrnpm	11.118181031-118181431	Usp36
14:56122088-56122488	Khnyn	6:117884504-117884904	Hnrnpf	3:79498365-79498765	Ppid
11.69691758-69692158	Fgf11	2.114485119-114485519	Dph6	6:122738013-122738413	Slc2a3
3:31148728-31149128	Skil	11.75423913-75424313	Slc43a2	2.25114884-25115284	Tubb4b
13:6697104-6697504	Pfkp	15:89240781-89241181	Ncaph2	10:128030894-128031294	Gls2
2.128809545-128809945	Zc3h6	5:137101083-137101483	Serpine1	3.108004011-108004411	Gnat2
1.30988594-30988994	Ptp4a1	9:108014719-108015119	4930447F2	19:23118299-23118699	Klf9
2.18069125-18069525	Mllt10	11.94101987-94102387	Tob1	7:30429239-30429639	Tmem147
1.128476322-128476722	Gm6170	4:134581152-134581552	Maco1	18:50111761-50112161	Tnfaip8
9:69360674-69361074	Anxa2	9:21996184-21996584	Ecsit	11.97206301-97206701	Mrpl45
2.112095915-112096315	Slc12a6	11.95304445-95304845	Spop	9:66031653-66032053	Snx1
2.25498225-25498625	Ccdc183	8:23747145-23747545	Golga7	11.68936563-68936963	Aurkb
9:21982238-21982638	Zfp653	1.21084683-21085083	Tram2	11.108234730-108235130	Apoh
2.126341536-126341936	Atp8b4	7:100355592-100355992	Fam168a	3.108118131-108118531	Atxn7l2
15:36283257-36283657	Rnf19a	4:46563951-46564351	Coro2a	6:34846399-34846799	Cyren
3:97065785-97066185	Acp6	9:48618788-48619188	Zbtb16	14:20132995-20133395	Saysd1
1.161078798-161079198	Prdx6	2:92276881-92277281	Slc35c1	10:3923534-3923934	Mthfd1l
3:54641317-54641717	Exosc8	19:3757443-3757843	Gm36608	10:80538161-80538561	Izumo4
1.64656024-64656424	Mettl21a	9:95739391-95739791	Atr	10:80538161-80538561	Mob3a
1.155848757-155849157	Cep350	9:115913883-115914283	Tgfbr2	14:56122088-56122488	Khnyn
4:8647054-8647454	Gm11809	16:21241735-21242135	Vps8	19:34856633-34857033	Pank1
3.146518897-146519297	Prkacb	2.22785189-22785589	Pdss1	6:120431703-120432103	Il17ra
2.103627690-103628090	Caprin1	1.20890314-20890714	Mcm3	14:12016309-12016709	Fhit
7:51511257-51511657	Gas2	18:67907790-67908190	Seh1l	5:77414893-77415293	Rest
11.85740081-85740481	Gm11444	3:32564923-32565323	Zfp639	18:84968964-84969364	Timm21
3.146205295-146205695	Gng5	9:22300487-22300887	Anln	2:73143526-73143926	Scrn3
19:7244503-7244903	Rcor2	3.154302563-154302963	Cryz	3:88997263-88997663	Rusc1
1.164076624-164077024	Slc19a2	8:71150089-71150489	Jund	19:42117518-42117918	Avpi1
4:118966130-118966530	Slc2a1	17:88281996-88282396	Msh6	16:31984249-31984649	Nrros
12.104985913-104986313	Gm47648	7:101582869-101583269	Numa1	19:53130488-53130888	Add3
4:59549161-59549561	Ptbp3	10:50469420-50469820	Ascc3	2.152581258-152581658	Id1
3:95726832-95727232	Rprd2	17:25068591-25068991	Hagh	4:99786816-99787216	Pgm1
1.180677934-180678334	Sde2	19:3818519-3818919	Kmt5b	11.20582333-20582733	Sertad2
2.120394130-120394530	Zfp106	6:148845815-148846215	Sinhcaf	14:54923506-54923906	Acin1
19:44272433-44272833	Gm35183	11.5738415-5738815	Dbnl	5:31045959-31046359	Agbl5
3:66867251-66867651	Gm43516	14:55797852-55798252	Dcaf11	5:123519984-123520384	Bcl7a
13:93441305-93441705	Homer1	8:75760514-75760914	Tom1	14:30850152-30850552	Smim4
5:31045959-31046359	Agbl5	7:45083777-45084177	Ruvbl2	8:72993791-72994191	Ap1m1
3:65865423-65865823	Ccnl1	10:81332362-81332762	Ncln	11.79145390-79145790	Wsb1
3:83947545-83947945	Tmem131l	6:13678137-13678537	Bmt2	14:54718940-54719340	Rem2
4:135582795-135583195	Srsf10	3:88204174-88204574	Cct3	17:29251254-29251654	Srsf3
11.88609584-88609984	Msi2	3.116601697-116602097	Agl	2.167922880-167923280	Pard6b
3:68951202-68951602	Trim59	10:79764070-79764470	Arid3a	5:137101078-137101478	Serpine1
3.133250017-133250417	Tet2	12:82294212-82294612	Sipa1l1	17:88281994-88282394	Msh6
1.22030623-22031023	Kcnq5	19:53301408-53301808	Mxi1	19:4147192-4147592	Cdk2ap2
3:89299131-89299531	Zbtb7b	7:28508198-28508598	Hnrnpl	4:106418104-106418504	Dhcr24
4:46450582-46450982	Anp32b	16:35759254-35759654	Parp9	3:95189176-95189576	Mindy1
9:77817116-77817516	Elovl5	4:124587398-124587798	Utp11	17:5239631-5240031	Arid1b
1.106642114-106642514	Bcl2	3.137674643-137675043	Dapp1	3:31148728-31149128	Skil
4:35225469-35225869	C9orf72	13:81858784-81859184	Polr3g	7:101582866-101583266	Numa1
1.176640754-176641154	Cep170	X:105230341-105230741	Pgk1	13:37240630-37241030	F13a1
3:89959764-89960164	Ubap2l	19:4559973-4560373	Pcx	9:52004531-52004931	Rdx

17:26999775-27000175	Bnip1	8:83109889-83110289	Il15	5:145137209-145137609	Zkscan14
8:25765296-25765696	Tacc1	17:25797931-25798331	Lmf1	17:80861298-80861698	Cdkl4
2:79528982-79529382	Ppp1r1c	2:71703412-71703812	Pdk1	16:75563720-75564120	Hspa13
14:31216292-31216692	Mettl6	1.33853047-33853447	Zfp451	4:108316869-108317269	Tut4
1.33796604-33797004	Bag2	1.93731206-93731606	Ing5	3:88204174-88204574	Cct3
2.130266464-130266864	Pced1a	13:51798841-51799241	Cks2	4:59549161-59549561	Ptbp3
3:69034381-69034781	Kpna4	11.58805846-58806246	Btnl10	7:100355594-100355994	Fam168a
10:59407129-59407529	Mcu	3.129326363-129326763	Elovl6	1.180677963-180678363	Sde2
17:23992893-23993293	Gm16275	18:24179679-24180079	Zfp24	15:63519815-63520215	Gm41335
3:60909957-60910357	P2ry1	8:13154872-13155272	Pcid2	11.77873128-77873528	Phf12
16:32883030-32883430	Gm17106	15:58952375-58952775	Mtss1	8:35431587-35431987	Tnks
16:32883030-32883430	Lmln	8:94905545-94905945	Mt1	9:56325774-56326174	Hmg20a
1.58691301-58691701	Flacc1	17:56440742-56441142	Sema6b	8:72973303-72973703	Fam32a
1.58691301-58691701	Gm20257	15:99870267-99870667	Larp4	14:73563212-73563612	Rb1
3.136542563-136542963	Ppp3ca	2:79465280-79465680	Itprid2	8:72085833-72086233	Unc13a
10:79889516-79889916	Gpx4	2.177598842-177599242	Gm14326	9:70849851-70850251	Lipc
10:42152631-42153031	Foxo3	17:47450682-47451082	Trerf1	11.4373814-4374214	Hormad2
6:122738008-122738408	Slc2a3	19:60779143-60779543	Eif3a	7:5018352-5018752	Fiz1
7:19449039-19449439	Tomm40	19:53261689-53262089	Gm36339	2.132094847-132095247	Pcna
2.164753980-164754380	Zfp335os	10:75695889-75696289	Mif	7:19006113-19006513	Vasp
7:126625382-126625782	Maz	8:85598609-85599009	Syce2	11.40585117-40585517	Mat2b
3.144086613-144087013	Gm5857	17:27720528-27720928	Grm4	12.4644937-4645337	Itsn2
17:80861298-80861698	Cdkl4	11.87938096-87938496	Srsf1	5:53666785-53667185	Rbpj
17:80861298-80861698	Gm33373	2.164720699-164721099	Pcif1	5:114461683-114462083	Myo1h
8:125675844-125676244	Egln1	5:93416136-93416536	Ccng2	11.116197497-116197897	Exoc7
4:59189224-59189624	Ugcg	16:4698041-4698441	Ubald1	17:56814582-56814982	Znrf4
3:88997263-88997663	Rusc1	6:34852107-34852507	Cyren	18:68433082-68433482	Rnmt
14:12016309-12016709	Fhit	2.131052113-131052513	Ap5s1	2:71283558-71283958	Metap1d
10:119948835-119949235	Helb	1.75168762-75169162	Atg9a	X:107876292-107876692	Brwd3
18:68433082-68433482	Fam210a	7:5018409-5018809	Fiz1	10:17898546-17898946	Abracl
18:68433082-68433482	Rnmt	5:31855647-31856047	Babam2	19:30007538-30007938	Uhrf2
1.178233578-178233978	Efcab2	15:78797979-78798379	Pdxp	15:80103457-80103857	Mgat3
2.22664138-22664538	Apbb1ip	7:27388741-27389141	Zfp607b	1.24717464-24717864	Lmbrd1
2:84670371-84670771	Slc43a1	1.121255650-121256050	Insig2	15:81756239-81756639	Aco2
13:73748180-73748580	Clptm1l	2:68691631-68692031	Cers6	15:81756239-81756639	Phf5a
1.171298299-171298699	F11r	2.181162084-181162484	Dnajc5	4:132495262-132495662	Rpa2
3:89986834-89987234	Tpm3	1.34478193-34478593	Ccdc115	19:8797176-8797576	Hnrnpul2
2.116950335-116950735	Spred1	1.34478193-34478593	Imp4	10:50469302-50469702	Ascc3
8:35431481-35431881	Tnks	2.25084741-25085141	Tor4a	2.126341536-126341936	Atp8b4
3.115801076-115801476	Extl2	3.103186783-103187183	Trim33	17:48005657-48006057	Prickle4
1.38036721-38037121	Txndc9	14:79628076-79628476	Naa16	17:48005657-48006057	Frs3
7:27185739-27186139	Sertad1	3.115682038-115682438	Dph5	11.87938096-87938496	Srsf1
4:133311702-133312102	Gpn2	14:78169638-78170038	Epsti1	10:128401791-128402191	Pa2g4
14:54718940-54719340	Rem2	14:56137505-56137905	Sdr39u1	17:73224327-73224727	Lbh
2:72306186-72306586	Cdca7	16:16120936-16121336	Yars2	8:110334998-110335398	Dhodh
4:40971074-40971474	Nfx1	7:29007046-29007446	Dpf1	7:139616244-139616644	Tubgcp2
2.53081776-53082176	Arl6ip6	19:5073907-5074307	Slc29a2	8:85751358-85751758	Get3
2.157121351-157121751	Rpn2	4:150321162-150321562	Eno1	7:80184686-80185086	Blm
4:133694476-133694876	Hmgn2	4:99082091-99082491	Atg4c	6:35154354-35154754	Nup205
2.167922880-167923280	Pard6b	7:120633476-120633876	Mettl9	7:27185744-27186144	Sertad1
2.180343334-180343734	Dido1	9:108525800-108526200	Arih2	5:139185949-139186349	Sun1
2.119424916-119425316	Oip5os1	7:46494990-46495390	Ldha	11.109363730-109364130	Slc16a6
1.44158007-44158407	Bivm	1.69726569-69726969	Ikzf2	9:107173680-107174080	Cish
6:86770553-86770953	Anxa4	3:88049859-88050259	Mef2d	19:5073907-5074307	Slc29a2
4:59438097-59438497	Susd1	3:79475057-79475457	Fnip2	3.138148362-138148762	Adh5

4:70328500-70328900	Cdk5rap2	4:44072501-44072901	Gne	15:78124048-78124448	Ncf4
2:71360108-71360508	Dlx1	8:111985537-111985937	Glg1	15:81614212-81614612	Rangap1
3.104772080-104772480	St7l	10:17598641-17599041	Cited2	14:52495386-52495786	Chd8
2.29951564-29951964	Set	16:37908039-37908439	Gsk3b	17:23085355-23085755	Zfp945
4:15957827-15958227	Nbn	5:77414893-77415293	Rest	10:89279496-89279896	Gas2l3
1.78635254-78635654	Acsl3	18:12299735-12300135	Rmc1	9:20518715-20519115	Fbxl12os
19:5971647-5972047	Cdc42ep2	19:53130498-53130898	Add3	2.164674910-164675310	Ctsa
2:75535015-75535415	Nfe2l2	9:94419259-94419659	Dipk2a	15:81283676-81284076	St13
2.34529076-34529476	Gm13414	3:52009635-52010035	Maml3	9:107166714-107167114	Mapkapk3
1.150040593-150040993	Ptgs2os2	1.167177677-167178077	Aldh9a1	19:6899471-6899871	Catsperz
16:32366054-32366454	Zdhhc19	2:84545403-84545803	Zdhhc5	17:56266095-56266495	Yju2
4:83191819-83192219	Ttc39b	7:64041745-64042145	Mphosph1	16:17066998-17067398	Hic2
2.26823297-26823697	Surf4	11.117133491-117133891	Septin9	5:100782787-100783187	Coq2
3:89870553-89870953	Atp8b2	11.98638242-98638642	Thra	15:97991062-97991462	Pfkm
7:63589329-63589729	Klf13	3.106389649-106390049	Dennd2d	10:59800316-59800716	Ddit4
9:88364503-88364903	Syncrip	7:80203994-80204394	Blm	9:95739391-95739791	Atr
1.93405898-93406298	Hdlbp	10:58282185-58282585	Ranbp2	11.58805848-58806248	Btnl10
2.32385174-32385574	Gm37169	8:71917141-71917541	Mrpl34	5:146769421-146769821	Rpl21
1.74429126-74429526	Ctdsp1	17:26421795-26422195	Rgs11	8:84625905-84626305	Adgrl1
3.108004011-108004411	Gnat2	15:68129805-68130205	Zfat	10:81332362-81332762	Ncln
14:60974534-60974934	Spata13	19:41900025-41900425	Pgam1	3:87813213-87813613	Hdgf
4:44012046-44012446	Clta	4:140787442-140787842	Crocc	8:65399854-65400254	Tmem192
2.152581258-152581658	Id1	17:27842973-27843373	Nudt3	6:145980936-145981336	Itpr2
8:110292481-110292881	Txnl4b	2.44817313-44817713	Gtdc1	3:35807841-35808241	Atp11b
2.25152388-25152788	Tprn	11.59054026-59054426	Iba57	7:105426862-105427262	Dchs1
5:139185949-139186349	Sun1	7:12711891-12712291	Zfp446	7:29007046-29007446	Dpf1
2.143852219-143852619	Rrbp1	3.108819046-108819446	Prpf38b	16:32366054-32366454	Zdhhc19
3:57643453-57643853	Rnf13	6:145980934-145981334	Itpr2	16:15964492-15964892	Spidr
2:73143526-73143926	Scrn3	19:5895797-5896197	Neat1	3:94839969-94840369	Selenbp1
18:46707317-46707717	Ticam2	11.102135938-102136338	Hrob	19:60779143-60779543	Eif3a
3:97674973-97675373	Pde4dip	12.51394721-51395121	G2e3	7:25454495-25454895	Hnrnpul1
4:43442119-43442519	Tesk1	4:108316867-108317267	Tut4	4:129636071-129636471	Khdrbs1
1.192855376-192855776	Traf3ip3	1.87477093-87477493	Neu2	19:6887195-6887595	Esrra
4:43562325-43562725	Creb3	9:57817840-57818240	Ubl7	2.14060882-14061282	Hacd1
4:25281645-25282045	Ufl1	2:90409837-90410237	Ptprj	17:29297578-29297978	Trp53cor1
2.152631205-152631605	Bcl2l1	12.32870027-32870427	Nampt	4:132237074-132237474	Sesn2
