# Supplementary material for: JAK2V617F reprograms Hypoxia Inducible Factor-1 to induce a non-canonical hypoxia regulon in myeloproliferative neoplasms
Source: Leukemia. 2026 Feb 2;40(3):609–21. doi: 10.1038/s41375-025-02843-9 (PMC12960217; doi:10.1038/s41375-025-02843-9)
Supplement: Supplementary file 3 — Supplementary Table S3 [file 41375_2025_2843_MOESM3_ESM.pdf]

Table S3. Identification of differentially expressed VF_Hx signature genes in ATACSeq and MsRNAseq datasets

	VF_Nx AND VF_Hx	VF_Nx ONLY	VF_Hx ONLY					WT_Hx ONLY				
Count	41	155	158					144				
				ATACSeq		Ms RNAseq (MEPs)			ATACSeq		Ms RNAseq (MEPs)	
				log2_FC	p_value	log2_FC	p_value		log2_FC	p_value	log2_FC	p_value
	Add3	2500002B13Rik	Abracl	0.0224	0.7211	1.5408	0.1942	Acp6	0.0978	0.2488	-1.4724	0.0098
	Adh5	4930447F24Rik	Acin1	-0.2981	0.5905	1.5789	0.0165	Acsl3	0.0776	0.2764	-2.2431	0.1373
	Arid3a	Agl	Aco2	-0.1244	0.1143	0.8678	0.0538	Acvrl1	ND		ND	
	Ascc3	Agrp	Adgrl1	ND	ND	-0.6298	0.0106	Anp32b	0.0137	0.2545	-0.4906	0.0161
	Atg4c	Ak4	Agbl5	-0.0648	0.7728	-2.3993	0.0210	Anxa2	0.0887	0.4860	3.3993	0.0051
	Atr	Aldh9a1	Anxa4	-0.1025	0.0247	1.0466	0.0045	Apbb1ip	-0.1211	0.0601	1.3113	0.0024
	Babam2	Alkbh5	Ap1m1	0.1625	0.0021	-1.6205	0.0239	Arl6ip6	-0.0197	0.6575	-0.7315	0.0679
	Blm	Anln	Apoh	ND	ND	ND	ND	Atf3	0.0355	0.4069	ND	
	Btnl10	Ap5s1	Arid1b	-0.0794	0.0133	-3.2067	0.0978	Atp8b2	0.0317	0.7803	-0.7483	0.6790
	Cct3	Arih2	Asic1	ND	ND	0.2173	0.8420	B4galt5	ND		1.1045	0.1602
	Cyren	Atg9a	Atp11b	-0.1243	0.0631	5.3482	0.0000	Bag2	-0.4047	0.0002	1.1428	0.2553
	Dpf1	Bmt2	Atp8b4	-0.1212	0.0398	3.9546	0.0046	Bcl2	ND		0.7168	0.0845
	Ecsit	Bnip3	Atxn7l2	-0.0954	0.4273	-2.3014	0.0847	Bcl7b	-0.1141	0.1539	-0.6532	0.1653
	Eif3a	Bsg	Aurkb	-0.1366	0.3360	-2.0335	0.0001	Bnip3l	-0.0271	0.8812	-0.2218	0.3977
	Exoc7	C2cd3	Avpi1	-0.0975	0.0769	-0.5834	0.0603	C9orf72	0.0407	0.2038	-2.0054	0.0967
	Fam168a	Ccdc115	Babam1	0.0072	0.7610	-1.9878	0.0275	Caprin1	-0.0586	0.3295	3.1875	0.0050
	Fiz1	Ccng2	Bcl2l1	0.0107	0.6747	-1.0661	0.0205	Ccdc183	0.0654	0.1139	ND	
	Grm4	Cers6	Bcl7a	-0.0214	0.8077	-1.0354	0.0000	Ccdc58	-0.0580	0.5985	2.2047	0.0138
	Gsk3b	Ciart	Bivm	0.0464	0.5524	0.2242	0.7360	Ccnl1	-0.0365	0.6508	3.6280	0.0016
	Hsp90b1	Cited2	Blm	0.0184	0.7215	2.5227	0.0091	Cdc42ep2	0.0788	0.3203	-2.0380	0.0013
	Itpr2	Cks2	Bnip1	0.0887	0.3523	-0.4842	0.0233	Cdca7	0.1419	0.0763	0.6679	0.1730
	Mgat3	Coro2a	Brat1	-0.1710	0.0150	-4.6107	0.0186	Cdk5rap2	0.0455	0.3189	-0.6296	0.1085
	Mrpl34	Crocc	Brwd3	0.1005	0.4031	ND	ND	Cep170	0.0212	0.8847	1.7621	0.0464
	Mrpl45	Crtap	Catspere2	ND	ND	ND	ND	Cep350	-0.0096	0.5411	-0.1962	0.3845
	Msh6	Cryz	Catsperz	ND	ND	ND	ND	Clptm1l	-0.0312	0.7611	-0.3299	0.1808
	Ncf4	Dapp1	Cda	-0.0215	0.9027	ND	ND	Clta	0.0803	0.0069	-3.7643	0.0569
	Ncln	Dbnl	Cdca3	-0.0412	0.2836	-0.6414	0.0064	CMTM7	-0.0001	0.8539	-0.8129	0.2129
	Numa1	Dcaf11	Cdk2ap2	-0.2158	0.0062	-2.2110	0.0031	Creb3	-0.0811	0.4438	1.4200	0.1620
	Psma1	Dennd2d	Cdkl4	0.0016	0.9945	-0.2633	0.7425	Ctdsp1	-0.0601	0.0773	-0.6531	0.1192
	Rbpj	Dipk2a	Chd8	0.0067	0.9109	-0.7929	0.0016	Dars	-0.0550	0.5406	0.7731	0.0116
	Rest	Dnajc5	Cish	0.0208	0.3524	2.6942	0.0509	Dido1	0.0438	0.3515	0.6168	0.0546
	Septin9	Dph5	Cmtm8	0.0903	0.0918	0.6884	0.4585	Dlx1	ND		ND	
	Serpine1	Dph6	Coq2	-0.1914	0.0072	ND	ND	Efcab2	-0.0268	0.3339	0.3253	0.2638
	Slc16a6	Elovl6	Ctsa	-0.0167	0.9450	-0.5692	0.0207	Egfl7	-0.0604	0.3301	-3.2626	0.0654
	Slc29a2	Eno1	Cux1	0.0532	0.3870	1.0931	0.0001	EGLN1	0.0790	0.3729	-0.7093	0.0055
	Spidr	Epsti1	Dchs1	0.0052	0.9777	ND	ND	Elovl5	0.0192	0.9834	0.1236	0.8558
	Srsf1	Ero1a	Ddit4	-0.0006	0.9931	-2.1869	0.0087	Exosc8	-0.1691	0.3256	0.613974	0.0057
	Ttc41	Fnip2	Dhcr24	0.0215	0.7920	-1.1844	0.0002	Extl2	0.0722	0.3067	0.5143	0.3152
	Tut4	Ftx	Dhodh	0.0309	0.8694	-4.0743	0.0132	F11r	-0.0850	0.1105	0.6809	0.2877
	Ubl7	G2e3	Dnaja1	0.0101	0.4232	0.6601	0.0207	Fam210a	0.0245	0.5733	-1.0828	0.0140
	Zfp609	Gapdh	Dok2	ND	ND	ND	ND	Fgf11	-0.1052	0.0989	1.6196	0.1235
		Gbe1	Dtwd2	-0.0219	0.5387	0.3299	0.4565	Flacc1	ND		ND	
		Glg1	Eif4ebp1	ND	ND	-1.6868	0.0027	Foxo3	-0.1010	0.0233	-1.0971	0.0000
		Gm12796	Eri2	-0.1070	0.0564	0.6115	0.0261	Fyb	ND		-0.6910	0.0432
		Gm14326	Esrra	-0.0850	0.2126	0.6531	0.1847	Gabpb2	-0.1280	0.0854	-3.4768	0.0545
		Gm2059	F13a1	ND	ND	1.9208	0.0138	Galk1	0.0261	0.2934	-1.8573	0.0082
		Gm31452	Fam117b	0.0123	0.5937	0.6845	0.2594	Gas2	0.4101	0.0000	-0.4111	0.3095
		Gm31805	Fam32a	0.1199	0.0837	-0.1592	0.4268	Gm11444	ND		ND	
		Gm32591	Fance	-0.1146	0.1279	-2.6220	0.0360	Gm11809	ND		ND	
		Gm36339	Fbxl12os	ND	ND	1.4010	0.0814	Gm13414	ND		ND	
		Gm36608	Fbxo10	0.1273	0.0579	-2.8196	0.0000	Gm16275	ND		ND	
		Gm49722	Fhit	0.1067	0.0570	ND	ND	Gm17106	ND		ND	
		Gm50149	Frs3	ND	ND	ND	ND	Gm20257	ND		ND	
		Gne	Gas2l3	ND	ND	-1.3025	0.3174	Gm33373	ND		ND	
		Golga7	Get3	ND	ND	-0.4939	0.0194	Gm37169	ND		ND	
		Gtdc1	Gls2	0.0059	0.8483	ND		Gm47648	ND		ND	
		Gtf2e2	Gm2a	ND	ND	0.0296	0.9212	Gm5857	ND		ND	
		Hagh	Gm41335	ND	ND	0.9873	0.2044	Gng5	-0.1218	0.1384	-0.3125	0.1209
		Hikeshi	Gnat2	ND	ND	1.5908	0.1336	Gpn2	-0.0784	0.4503	-1.9108	0.0058
		Hilpda	Hacd1	ND	ND	0.5051	0.3022	Gpx4	0.0884	0.2383	1.7831	0.1078
		Hnrnpf	Hdgf	0.0264	0.9599	0.0891	0.9129	Hdlbp	0.0178	0.7451	0.9864	0.0112
		Hnrnpl	Hic2	ND	ND	-0.3748	0.2106	Helb	-0.0246	0.7773	1.8790	0.0965
		Hnrnpm	Hk1	0.0485	0.4923	-2.6444	0.0003	Higd1a	-0.1308	0.0193	-1.0516	0.2800
		Hrob	Hmg20a	-0.0247	0.8911	1.2678	0.0301	HK2	-0.0312	0.5260	ND	
		Iba57	Hnrnpul1	0.0237	0.2783	1.2792	0.0283	Hmgn2	-0.1609	0.0706	-1.1254	0.0666
		Ikzf2	Hnrnpul2	-0.1087	0.1912	-0.6879	0.0047	IER3	0.0154	0.8028	-0.2601	0.2837
		Il15	Homer1	0.0095	0.8937	0.6675	0.0890	Jmjd6	-0.0247	0.7323	2.6479	0.0822
		Imp4	Hormad2	ND	ND	0.2549	0.8824	Kcnq5	0.0592	0.5856	0.9775	0.3506
		Ing5	Hspa13	0.0599	0.3649	0.2017	0.4199	Klf13	-0.0816	0.0081	-1.0685	0.0000
		Insig2	Id1	0.2043	0.1419	ND	ND	Kpna4	-0.0610	0.2791	-0.1956	0.5849
		Itprid2	Il17ra	0.0146	0.8001	-3.1360	0.0074	Lmln	0.0690	0.3864	-0.0402	0.9281
		Jund	Itsn2	-0.0753	0.1524	4.2532	0.0000	Lrmda	ND		-3.0995	0.0026
		Kdm4b	Izumo4	-0.0112	0.9277	-1.2484	0.4196	Maz	-0.1695	0.0047	-1.5074	0.0152

		Kmt2e	Kctd20	-0.0109	0.8968	1.1724	0.0248	Mcu	-0.1224	0.0464	-0.6358	0.0233
		Kmt5b	Khdrbs1	-0.0356	0.5791	-0.7592	0.0048	Mettl21a	-0.0598	0.3942	-0.4612	0.4171
		Kpna7	Khnyn	-0.0496	0.5662	-0.6585	0.0321	Mettl23	-0.0790	0.1954	-0.9449	0.0014
		Larp4	Khsrp	-0.0055	0.7076	-1.8919	0.0000	Mettl6	-0.0949	0.2379	0.6016	0.0190
		Ldha	Klf9	-0.0817	0.0960	-0.2292	0.3372	Mga	ND		-3.5223	0.0096
		Lmf1	Lbh	-0.1824	0.0017	1.4050	0.0747	Mllt10	-0.1145	0.0074	-0.6009	0.0047
		Maco1	Lipc	0.2387	0.0000	ND	ND	Msi2	-0.1693	0.0000	1.0260	0.0036
		Maml3	Lmbrd1	ND	ND	-0.4374	0.0448	NAIP	ND		0.5676	0.5009
		Mcl1	M1ap	0.0903	0.1619	-0.9628	0.0103	Nbn	0.0195	0.5482	-0.4831	0.1395
		Mcm3	Mapkapk3	-0.0282	0.7345	-1.4119	0.0163	NFE2L2	-0.0862	0.0910	0.9709	0.0138
		Med18	Mat2b	-0.1157	0.1917	-1.1419	0.0098	Nfx1	0.1026	0.0088	-0.6214	0.0022
		Mef2d	Mdm2	0.0152	0.6628	-4.7775	0.0404	Oip5os1	ND		0.4755	0.0663
		Mettl9	Metap1d	-0.0775	0.3195	0.9642	0.0077	P2ry1	-0.0818	0.5047	-0.6746	0.3499
		Mfsd8	Mindy1	ND	ND	2.5640	0.0119	Pced1a	-0.1284	0.2674	-1.0066	0.0018
		Mif	Mob3a	ND	ND	-1.0969	0.0013	Pde4dip	-0.1698	0.2214	1.6266	0.1173
		Mphosph10	Mthfd1l	0.0988	0.2135	-2.3469	0.0000	Pfkp	-0.0191	0.7376	1.7214	0.0059
		Mt1	Myo1h	ND	ND	-1.7252	0.0005	Plekha2	-0.0437	0.1559	0.1945	0.3679
		Mtss1	Nrros	-0.0560	0.4837	-1.5416	0.0030	Ppp3ca	-0.0251	0.2977	2.1786	0.0080
		Mxi1	Nudt6	0.0387	0.7670	-1.7812	0.0174	Prdx6	-0.0062	0.7983	-0.6250	0.0162
		Naa16	Nup205	-0.1168	0.2230	1.7327	0.0190	Prkacb	-0.1577	0.0040	1.449576	0.0920
		Nampt	Pa2g4	-0.1176	0.1381	-1.2240	0.0000	Ptgs2os2	ND		ND	
		Narf	Pank1	-0.0347	0.6130	-3.5241	0.0318	Ptp4a1	-0.0506	0.5158	-0.3438	0.2872
		Ncaph2	Pard6b	0.0086	0.8998	-1.7855	0.0083	Pygo2	0.0839	0.3112	-4.5206	0.0060
		Neat1	Pcna	-0.1132	0.0402	1.3462	0.0001	Rara	-0.0756	0.0569	-1.0198	0.5541
		Neu2	Pfkm	-0.1146	0.1857	-1.5951	0.0000	Rcor2	0.1270	0.1578	-0.6755	0.6192
		Npm1	Pgm1	-0.0256	0.6891	-0.8433	0.0305	Rnf113a1	-0.0044	0.8104	-0.4441	0.0371
		Nudt3	Phf12	-0.1431	0.0202	-0.7301	0.0013	Rnf13	ND		2.0777	0.0179
		Parp9	Phf5a	-0.0602	0.6062	-0.6635	0.0249	Rnf19a	0.1322	0.0074	-1.2460	0.0069
		Pcgf5	Polg	-0.0421	0.4781	-4.7052	0.0074	Rnf7	-0.0288	0.6667	-0.1786	0.3722
		Pcid2	Ppia	-0.0578	0.4633	1.8435	0.0072	Rpn2	-0.0622	0.3447	0.6946	0.0055
		Pcif1	Ppid	-0.0709	0.6687	2.6889	0.0757	Rprd2	-0.0435	0.3487	0.8010	0.1827
		Pcx	Ppp1r1c	0.3021	0.0115	ND	ND	Rrbp1	0.1374	0.0093	-0.6186	0.0657
		Pdk1	Prickle4	-0.1225	0.1068	ND	ND	Safb2	0.1668	0.0034	3.0218	0.0038
		Pdss1	Ptbp3	ND	ND	-5.0577	0.0425	Set	0.0168	0.3178	-0.7712	0.0035
		Pdxp	Rangap1	-0.0126	0.5608	-2.7535	0.0047	Shc4	0.0267	0.5273	-1.4678	0.0000
		Pgam1	Rb1	-0.0508	0.7834	1.5416	0.0169	Slc12a6	-0.0990	0.1274	0.7296	0.0073
		Pgk1	Rbp7	ND	ND	1.2211	0.2074	Slc19a2	-0.0545	0.4836	-0.9641	0.3127
		Phtf1	Rdx	ND	ND	-4.1539	0.0417	Slc2a1	-0.1854	0.0007	-0.4940	0.29469
		Polr3g	Rem2	0.0331	0.8030	-1.5814	0.0555	Slc43a1	-0.1042	0.1317	-3.0392	0.0329
		Prpf38b	Rexo5	ND	ND	0.8818	0.0013	Spata13	0.0324	0.4174	-0.9998	0.1829
		Ptprj	Rnmt	-0.0271	0.7292	0.5324	0.0182	Spred1	-0.0120	0.9315	3.1021	0.0030
		Ranbp2	Rpa2	-0.0663	0.5750	-0.5893	0.0037	Srsf10	0.0796	0.1914	-0.9648	0.0133
		Rap1gds1	Rpl21	0.0274	0.7969	-0.3141	0.1746	St7l	0.0741	0.3501	-3.6838	0.0486
		Rgs11	Rusc1	-0.0095	0.7951	-1.3189	0.0184	Surf4	0.0254	0.8010	-4.4550	0.0117
		Rmc1	Saysd1	-0.0767	0.3002	-0.7686	0.0058	Susd1	0.2631	0.0000	3.3281	0.0019
		Rnf145	Scrn3	0.0572	0.5168	-0.8524	0.0000	Syncrip	-0.0444	0.4214	-1.2429	0.0085
		Ruvbl2	Sde2	-0.0416	0.1223	-0.9624	0.0000	Tacc1	0.0672	0.0462	-0.6373	0.0021
		Sacm1l	Selenbp1	ND	ND	-1.1665	0.0000	Tesk1	0.0214	0.2106	-3.0442	0.0107
		Sdr39u1	Sertad1	0.0234	0.4931	-1.4131	0.0049	Tet2	-0.2865	0.0000	-4.5487	0.0154
		Seh1l	Sertad2	-0.0604	0.5061	-0.8989	0.0010	Ticam2	ND		0.7512	0.0404
		Sema6b	Sesn2	-0.1436	0.1276	-2.8029	0.0000	Tmem131l	ND		1.7757	0.1536
		Sil1	Skil	0.0367	0.4566	0.9684	0.0426	Tomm40	0.0985	0.0957	0.9510	0.0952
		Sinhcaf	Slc2a3	ND	ND	1.2811	0.0003	Tpm3	0.0426	0.5613	-0.7538	0.0017
		Sipa1l1	Smim4	-0.1490	0.0231	0.3669	0.1778	Tprn	0.0642	0.3051	-1.6875	0.0000
		Slc35c1	Snx1	0.0156	0.7453	-0.6050	0.0030	Traf3ip3	-0.1077	0.0928	0.6246	0.0480
		Slc43a2	Srsf3	-0.1010	0.2066	3.8115	0.0036	Trim59	-0.1755	0.0319	-0.1265	0.6081
		Spop	St13	-0.2299	0.0031	0.8913	0.0005	Trmo	ND		0.1961	0.6614
		Syce2	Strip1	-0.0797	0.2862	-1.8559	0.0058	Ttc39b	0.1413	0.0088	0.3798	0.1038
		Tgfbr2	Stxbp2	0.0939	0.1897	1.5671	0.2005	Tubb5	ND		-1.1194	0.1522
		Thra	Sun1	-0.0167	0.8440	-1.9502	0.0107	Txndc9	-0.1125	0.1423	0.6148	0.0167
		Tob1	Syvn1	-0.0516	0.5104	-6.6369	0.0000	Txnl4b	-0.0361	0.6743	-0.5935	0.0207
		Tom1	Tent2	ND	ND	2.4213	0.0192	Ubap2l	-0.0360	0.9712	-6.7411	0.0031
		Tor4a	Timm21	-0.0226	0.7245	0.2948	0.2296	Ufl1	0.0859	0.4147	-0.3033	0.1573
		Tram2	Tmem147	0.1888	0.1065	-1.3386	0.0082	Ugcg	0.1297	0.0684	0.8283	0.0483
		Trerf1	Tmem192	-0.0249	0.6713	-1.4587	0.0223	Usf1	-0.0478	0.6708	-0.6878	0.0014
		Trim33	Tmigd3	ND	ND	ND	ND	WIPF1	0.0131	0.3905	-0.0238	0.9806
		Triobp	Tnfaip8	-0.1610	0.0001	2.0930	0.0581	Zbtb7b	-0.0145	0.8393	-2.0332	0.1841
		Tut7	Tnks	0.0049	0.9851	-0.2611	0.2457	Zc3h6	-0.1618	0.0453	0.0534	0.8567
		Ubald1	Trappc5	0.0377	0.3727	-1.5558	0.0143	Zfp106	ND		-2.2667	0.0154
		Utp11	Trp53cor1	ND	ND	0.6225	0.5359	Zfp335os	ND		-0.9236	0.1213
		Vdac1	Tubb4b	0.0815	0.1143	-1.4589	0.0000	Zfp653	ND		-1.0494	0.1271
		Vps8	Tubgcp2	-0.1666	0.0093	1.0410	0.0429					
		Wipf1	Uhrf2	0.1580	0.0489	1.5678	0.0000					
		Yars2	Unc13a	ND	ND	-0.1981	0.4672					
		Zbtb16	Usp36	0.0378	0.6478	2.9738	0.0118					
		Zdhhc5	Usp5	-0.0650	0.1245	-2.6077	0.0055					
		Zfat	Vasp	-0.0722	0.3050	-1.2881	0.0106					
		Zfp24	Wsb1	-0.0640	0.5865	1.0096	0.0023					
		Zfp446	Yju2	ND	ND	0.9086	0.0025					

			Zfp451		Zdhhc19	ND	ND	1.0592	0.1475						
			Zfp607b		Zfp945	ND	ND	2.9587	0.0189						
			Zfp639		Zkscan14	ND	ND	-0.7131	0.0202						
					Zmiz1	ND	ND	2.2227	0.0174						
					Znhit3	ND	ND	0.6369	0.1567						
					Znrf4	ND	ND	ND	ND						
