# Supplementary material for: JAK2V617F reprograms Hypoxia Inducible Factor-1 to induce a non-canonical hypoxia regulon in myeloproliferative neoplasms
Source: Leukemia. 2026 Feb 2;40(3):609–21. doi: 10.1038/s41375-025-02843-9 (PMC12960217; doi:10.1038/s41375-025-02843-9)
Supplement: Supplementary file 4 — Supplementary Table S4 [file 41375_2025_2843_MOESM4_ESM.pdf]

Table S4. Patient and healthy donors included in scRNAseq analysis (original dataset GSE144568)

	Donor ID	scRNAseq sample ID	Age	Sex	Disease Status: Primary (PMF); Secondary to ET or PV (PPV MF / PET MF) or Control	Mutations
JAK2 only	010/003	ID21	79	M	PPV MF	JAK2 c.1849G>T p.(Val617Phe)
	010/054	ID20	77	M	PPV MF	JAK2 c.1849G>T p.(Val617Phe)
	010/022	ID19	65	F	PPV MF	JAK2 c.1849G>T p.(Val617Phe)
	001/131	ID05	49	F	PPV MF	JAK2 c.1849G>T p.(Val617Phe)
	001/132	ID10	75	M	PPV MF	JAK2 c.1849G>T p.(Val617Phe)
JAK2+	001/124	ID11	70	M	PMF	JAK2 c.1849G>T p.(Val617Phe); ASXL1 c.1774C>T p.(Gln592Ter)
	001/118	ID12	73	M	PPV MF	JAK2 c.1849G>T p.(Val617Phe); ASXL1 c.2362G>T p.(Glu788Ter); ASXL1 c.2421del p.(Pro808LeufsTer10); ETV6 c.1130C>T p.(Ala377Val)
	001/114	ID14	68	M	PPV-MF	JAK2 c.1849G>T p.(Val617Phe); ASXL1 c.1934dupG p.(Gly646TrpfsTer12)
	001/038	ID02	60	M	PPV MF	JAK2 c.1849G>T p.(Val617Phe); ASXL1 c.1926_1927insG p.(G646Wfs*12)
	001/040	ID03	73	F	PPV MF	JAK2 c.1849C>T p.(Val617Phe); TET2 c.4835delC p.(Ser1612LeufsTer4)
	001/110	ID07	69	M	PPV MF	JAK2 c.1849G>T p.(Val617Phe); ASXL1 c.1720-1G>C; EZH2 c.2191del p.Tyr731ThrfsTer9
Healthy	012/9002	ID17	53	M	Healthy donor control	none
	012/9006	ID18	51	F	Healthy donor control	none
	009/001	ID01	67	F	Healthy donor control	none
	009/005	ID06	60	M	Healthy donor control	none
	009/007	ID09	56	F	Healthy donor control	none
	009/008	ID13	55	M	Healthy donor control	none
