# Supplementary material for: JAK2V617F reprograms Hypoxia Inducible Factor-1 to induce a non-canonical hypoxia regulon in myeloproliferative neoplasms
Source: Leukemia. 2026 Feb 2;40(3):609–21. doi: 10.1038/s41375-025-02843-9 (PMC12960217; doi:10.1038/s41375-025-02843-9)

Supplementary Information

Supplementary Methods

Chromatin Immunoprecipitation Next Generation Sequencing: Library preparation was performed using the NEBNext DNA Ultra II library preparation kit for Illumina and performing 12 cycles PCR amplification with unique dual indexed primers. Libraries were pooled at equimolar ratios, and subject to 150 base paired end sequencing on an Illumina NovaSeq 6000 at Azenta Life Sciences.

Next Generation Sequencing Analysis: Read quality was assessed with FastQC v0.11.7 (1), removing any retained adapters and poor quality reads using a sliding window approach with trimmomatic v0.36 (2). Reads were discarded when trimmed to less than 50 base pairs and only intact read pairs were retained. Reads were aligned to the GRCm39 primary mouse assembly with STAR v2.7.10a (3) and annotated with SAMtools v1.15 (4) fixmate and markdup. ENCODE mm10 'blacklist' regions (5) were removed after GRCm39 UCSC liftOver using BEDTools v2.30.0 (6). Peaks were called with MACS v2.2.7.1 (7) and pairwise differential binding assessed using DiffBind v3.14.0 (8) in R v4.3.3 using all replicates and input controls. Absolute $\log_2$ fold changes greater than 1 and FDR-corrected p values less than 0.05 were considered in the first instance, with FDR-based ranking also used to assess condition-specific HIF-1α binding importance. Relevant peak locations were plotted using karyoploteR v1.3.0 (9). Peak locations were annotated using ENCODE and GENCODE GRCm39 annotations and HREs detected using Jellyfish 2.3.0 (10).

Gene signature derivation: Pairwise differential binding was assessed using DiffBind v3.14. in R v4.3.3 and annotated using ENCODE and GENCODE GRCm39 annotations, as above. Absolute $\log_2$ fold changes of median peak intensity (MPI) greater than 1 and FDR-corrected p values less than 0.05 for JAK2 WT hypoxic relative to normoxic, and for JAK2V617F normoxic or hypoxic (pathological) relative to JAK2 WT hypoxic (physiological). The top 200 enriched genes (FDR-corrected p values less than 0.03, binomial test less than

0.05 were included in the signatures) (11). Disparate genes between the VF_Hx/VF_Nx signatures (n=158), and VF_Hx/WT_Hx signatures (n=144), were then identified as gained and lost HIF-1 targets, respectively, in VF_Hx cells (Fig. S5A). To delineate genes that are acquired as HIF-1 targets because of chromatin alterations, genes with altered accessibility in scATAC-seq were removed (12). Genes were then analysed for differential expression in bulk and scRNAseq JAK2V617F vs WT (13,14), and differentially expressed genes were analysed for correlation with progression/survival and AML transformation in the JAK2V617F MPN patient cohort.

Investigation of VF_Hx genes in biologically motivated gene sets: Investigation of VF_Hx genes in biologically motivated gene sets JAK2V617F murine hematopoietic stem and progenitor (HSPC) bulk RNAseq (14), JAK2V617F Myelofibrosis (MF) patient single cell ATACseq (Table S2), JAK2V617F MF patient scRNAseq (13) (Table S3) and the 172-JAK2V617F MPN patient cohort was conducted in the following workflow. Disparate genes between the VF_Hx/VF_Nx signatures (n=158), and the VF_Hx/WT_Hx signatures (n=144), representing those HIF-1 genes gained and lost, respectively, in the VF_Hx context. Genes with significantly altered accessibility (12), that were acquired as HIF-1 targets as a result of chromatin alterations and not aberrant HIF-1 function, were removed. Remaining genes were analysed for significant differential expression in bulk and scRNAseq JVF vs WT. The HIF1-MPN-BP signature was derived from analysis of these differentially expressed genes in 11 matched patient samples pre- and post leukemic transformation to MPN-BP; correlation with progression/survival and AML transformation was analysed. Signature enrichment in normal hematopoiesis was assessed with BoneMarrowMap (15). For clinical AML cohorts, we leveraged logTPM normalized RNA-sequencing data and clinical data compiled from a larger meta-analysis performed by Severens et al. comprising 1079 patients spanning the TCGA, BEAT-AMLv2, Leucegene and LUMC cohorts (16). ICC 2022 classes were available for 1058 patients and used to determine patients' corresponding 2022 European LeukemiaNet (ELN) classes (17). For survival analysis in AML, we compiled overall survival

data from 1103 patients across 3 cohorts (TCGA, BEAT-AMLv2, GSE6891) (18–20). We predicted patient survival through a multivariate Cox regression stratified by patient cohort.

RIME Mass spectrometry: Protein was on-bed digested with trypsin, without reduction or alkylation. Peptides were desalted by C18 ZipTip before drying and resuspending in aqueous 0.1% TFA for LC-MS. Peptides were acquired over a 1 h acquisition with elution from a 50 cm EN PepMap C18 column driven by a Waters mClass UPLC onto an Orbitrap Fusion Tribrid mass spectrometer operated in DDA mode. MS1 spectra were acquired in the Orbitrap mass analyser at 120K resolutions and a cycle time of 1 s, with MS2 spectra acquired in parallel in the linear ion trap using TopSpeed selection. LC-MS chromatograms in .raw format were imported into Progenesis QI for peak picking and chromatographic alignment, before exporting a concatenated tandem mass list in .mgf format for Mascot database searching. Searching was performed against the mouse subset of the SwissProt database appended with common proteomic contaminants. Peptide spectral matches were filtered to 1% FDR using the percolator algorithm and as assessed empirically against a reverse database search. Accepted peptide identifications were imported into Progenesis QI, associated with the chromatographic data and mapped between runs. Data were further filtered to require a minimum of two unique peptides per protein quantification. Relative protein quantification was inferred from peptide precursor ion areas using unique, non-conflicting peptides only and after normalisation to total peptide intensity among runs. Statistical testing between groups was done using anova with the null hypothesis being that proteins are of equal abundance between all samples. The Hochberg and Benjamini FDR methodology was used for multiple test correction of p-values to q-values. For STRING analysis, structural and non-mouse proteins were discarded, RIME hit proteins were sorted by geomean (relative to HIF-1α) and those less than 0.01 discarded. The remaining proteins were then run through the STRING analysis online database (STRING: functional protein association networks) and clustered via MCL clustering (stringency 3). Resulting connections plotted by confidence (cutoff ‘medium’ – 0.04).

Phosphoproteomics: Samples immunoprecipitated for HIF-1α (see above) were digested on-bead using the RIME protocol and trypsin as the protease, before TiO_2 (MagReSyn) phosphopeptide enrichment. Resulting peptides were loaded onto an EvoTip Pure for introduction onto an 8 cm performance column. Elution was carried out using a 60 SPD pre-set gradient on an EvoSep One UPLC. DDA-PASEF data were acquired using a Bruker timsToF HT. Data were searched through FragPipe for phosphopeptide identification and relative quantification at 1% FDR. Statistical testing was performed for all pairwise comparisons using limma via FragPipe-Analyst. Zero value imputation was applied and the Hochberg and Benjamini approach was used for multiple test correction. Significance threshold was set at $q < 0.05$.

Cell culture: All cell lines were cultured in R10 media (RPMI (Gibco), 10% FBS (Gibco), 1% penicillin/streptomycin (Gibco)/L-glutamine (Gibco) except SET2 cells which were cultured in R20 (as above, 20% FBS). Cells were incubated at 5% CO_2 and the indicated O_2 concentrations. hMPL BaF3 cells were cultured with murine IL-3 (Peprotech) and UT7/TPO cells were cultured with human TPO (Peprotech). BaF3/hMPL, UT-7/TPO, SET2 and HEL cells were a gift from Prof. Ian Hitchcock. Cell lines were routinely mycoplasma tested.

Cell fractionation: UT7/TPO cells were cultured as indicated and then starved overnight in 2% FBS RPMI (Roswell Park Memorial Institute (RPMI) 1640 Medium (Gibco)) media with no TPO prior to treatment. Cells were treated as indicated with 10μM Roxadustat (Cayman Chemical #15294), 2μM Ruxolitinib (Cayman Chemical #11609), 5μM GN44028 (Cayman Chemical #37289) or DMSO control for 24hrs in hypoxic (1% O_2) conditions. Cells were split into cytoplasmic, nuclear soluble and chromatin-bound fractions using the Subcellular Protein Fractionation Kit for Cultured Cells (Thermo Scientific™ #78840) following manufacturer's instructions. Cell fractions were stored in their final elution buffers at -80°C until use.

Western blot: Cells were cultured as above and treated with either 4mM NAC (Biotechne #7874), 10μM (or dose as indicated on figure) SMI-4a (Cayman Chemical #11029), (dose as

indicated on figure) TCS PIM1.1 (Santa Cruz Biotechnology #sc-204330), (dose as indicated on figure) PIM447 (Selleckchem #S7985), 2µM Ruxolitinib (Cayman Chemical #11609), 5µM GN44028 (Cayman Chemical #37289), 10µM Roxadustat (Cayman Chemical #15294), (dose as indicated on figure) SCH874 (Selleckchem #S7101) or vehicle control (DMSO) as indicated. Cells were lysed in cell lysis buffer (20 mM Tris-HCl (pH 7.5), 150 mM NaCl, 1 mM Na_2EDTA, 1 mM EGTA, 1% Triton) plus Halt™ Protease and Phosphatase Inhibitor Cocktail, EDTA-free (ThermoFisher Scientific). Lysate (20-30 µg) was separated on 4-20% or Any kD™ Mini-PROTEAN® TGX Stain-Free™ protein gels (Biorad) and transferred onto Immun-Blot® Low Fluorescence PVDF membrane (Biorad). Membranes were probed for the indicated antibodies (list below).

Chromatin-Immunoprecipitation-qPCR: UT7/TPO cells were cultured as indicated, then $14x10^6$ cells per condition were starved overnight in 2% FBS RPMI (Roswell Park Memorial Institute (RPMI) 1640 Medium (Gibco)) media with no TPO prior to treatment. Cells were treated as indicated with 10µM Roxadustat (Cayman Chemical #15294), 2µM Ruxolitinib (Cayman Chemical #11609), 5µM GN44028 (Cayman Chemical #37289) or DMSO control for 24hrs in hypoxic (1% O_2) conditions. Cells were fixed by the addition of 1% formaldehyde (Cell Signalling Technology) for 10 minutes at room temperature (RT), then neutralised by the addition of 10x Glycine for 5 minutes at RT. Samples were then processed using SimpleChIP® Enzymatic Chromatin IP Kit, Magnetic Beads (Cell Signalling Technology) according to the manufacturer's protocol. ChIP was performed with HIF-1α (D1S7W) XP® Rabbit mAb (Cell Signalling Technology). Eluted gDNA was analysed by qPCR as described below with cycle number increased to 45.

Apoptosis assay: UT7/TPO cells were cultured as indicated. $4x10^4$ Cells were treated with indicated dose of Roxadustat (Cayman Chemical #15294), Ruxolitinib (Cayman Chemical #11609), GN44028 (Cayman Chemical #37289) or DMSO control for 72hrs in either normoxic (20% O_2) or hypoxic (1% O_2) conditions. Cell staining was performed with APC Annexin V Apoptosis Detection Kit with PI (Biolegend #640932) and stained cells were

analysed by flow cytometry using a CytoFLEX LX355 flow cytometer. See Fig. S7M for gating strategy (sample used: JAK2 WT 20µM GN44028 Hx).

Bulk RNAseq and analysis: Bulk RNAseq data of JAK2V617F knock-in mice re-analysed from publicly available dataset (GEO: GSE180853). RNA extracted, prepared and sequenced as per method specified in paper (14). Briefly, RNA extracted by TRIzol extraction from sorted-cell populations as indicated. RNA-seq libraries were generated by 3′ sequencing and SMART-Seq2 amplification and sequenced on an Illumina HiSeq 4000. SRA short read RNA-seq analysis performed as follows; raw, paired-end Illumina reads downloaded from NCBI SRA were first trimmed with Cutadapt (version 2.10) (21) to remove the Illumina universal adapters and any low-quality sequence, with a minimal length threshold of 20bp. Trimmed reads were mapped and quantified using the Salmon (version 1.10) (22) pseudo-aligner, using the GENCODE M32 (GRCm39) (23) Mus musculus transcriptome, specifying the ISR library type, 100 bootstraps, and the "validateMappings" option. Differential expression analysis was run using the Sleuth (version 0.30.1) (24) and Wasabi (version 1.0.1) (25) packages in R, on a gene-wise basis, and using the Wald test to find statistically significant, differentially expressed genes. Genes with a log2 fold change of greater than +/-1 and with a q value of less than 0.1 were taken to be significantly differentially expressed.

scRNAseq and analysis: scRNAseq of MPN patients and healthy controls taken from publicly available dataset (GEO: GSE144568). RNA extracted, prepared and sequenced as per method specified in paper (13). SRA single cell RNA-seq analysis performed as follows; CellRanger files downloaded from NCBI SRA were loaded into the Seurat package (version 5.1.0) (26) and the Read10X function. Samples read in separately and were filtered to exclude genes expressed in fewer than 3 cells, and to exclude cells with fewer than 500 genes expressed. Cells with greater than 10% mitochondrial gene expression were excluded. Filtered samples were merged and then normalised, scaled, and variable features identified. Layers in the merged object were integrated with the "RPCAIntegration" method.

The “FindNeighbors” function was run using the first 30 principal components, followed by FindClusters function with a resolution of 0.25. The RunUMAP function was used to calculate the UMAP dimensional reduction, again with the first 30 principal components. Marker genes for the UMAP clusters were found by first joining the layers with the JoinLayers function and then using the FindAllMarkers function. The UMAP clusters were renamed according to their cell types, by manually assessing the marker genes for each cluster. HIF gene module scores were calculated by providing a list of HIF-associated genes to the R UCell package (27) and the “AddModuleScore_UCell” function, and added to the Seurat object to visualise the HIF gene module scores for the cells in their respective UMAP clusters.

RT and qPCR: RNA was extracted from samples using Monarch® Total RNA Miniprep Kit #T2010S and reverse transcription performed using LunaScript RT Master Mix Kit (Primer-free) #E3025S (New England Biolabs) as per manufacturer protocols with SimpliAmp™ Thermal Cycler (Applied Biosystems™ #A24811). qPCR was performed with SYBR™ Green PCR Master Mix (Applied Biosystems™ #4309155) and primers listed below. StepOnePlus™ Real-Time PCR System (Applied Biosystems™ #4376600), QuantStudio 3 Real-Time PCR System (ThermoFisher Scientific) or QuantStudio 7 Pro Real-Time PCR System (ThermoFisher Scientific) were used and analysis performed on StepOne or QuantStudio Software.

Statistics: Unless otherwise stated, statistical analyses were performed using R or GraphPad Prism and are described in each figure legend. Sample sizes for *in vitro* assays were determined based on power analyses to ensure statistical significance, aiming to detect predefined effect sizes with adequate statistical power, typically indicated by a p-value of less than 0.05. Where systematic differences existed between experimental runs, data were analysed with mixed-model ANOVA allowing a separate intercept and effect of time for each run. Homoscedasticity and normality of all model residuals were evaluated graphically. Where residuals were non-normal or heteroscedastic, the model was refit to the $\log_{10}$-

transformed dependent variable. If model assumptions were still not met, nonparametric tests or Welch-corrected Student's *t*-tests were used instead. Where data are normalised to a group, this group was excluded from the analysis. One-sample Student's *t*-tests were used when comparing data to a standardised group (theoretical value of 1).

Data Availability: BaF3/hMPL JAK2 WT / JAK2V617F HIF-1α ChIP-seq data is available at NCBI SRA PRJNA1144472.

cDNA primers (qPCR):

**Target**	**Forward**	**Reverse**
hACVRL1	CGAGGGATGAACAGTCCTGG	GTCATGTCTGAGGCGATGAAG
hAURKB	CAGTGGGACACCCGACATC	GTACACGTTTCCAAACTTGCC
hBLM	CAGACTCCGAAGGAAGTTGTATG	TTTGGGGTGGTGTAACAAATGAT
hCMTM7	TGCGACTTGATAATGATCCTCG	CACGCGGTAGAAGCGGAAG
hEGLN1	GAAGGCGAACCTGTACCCC	TTCATGCACGGCACGATGTA
hHK2	GAGCCACCACTCACCCTACT	CCAGGCATTCGGCAATGTG
hIER3	GCAGCCGCAGGGTTCTCTACC	CTCTTCAGCCATCAGGATC
hNAIP	AAGGGATTTGTTGACATAACGGG	CAGCCGTAGTTCTTCGTAAGC
hNFE2L2	TCAGCGACGGAAAGAGTATGA	CCACTGGTTTCTGACTGGATGT
hNUDT6	ATGCTTGCCCGAACCTACG	GAGATGCCCCCGAATCTGTC
hPCNA	CCTGCTGGGATATTAGCTCCA	CAGCGGTAGGTGTCGAAGC
hTNKS	TGGACGCGGCAAACGTAAAT	AAGCGGGATGAGACCTCCAT
hWIPF1	CGGAGGCGGTGGAAGTTTT	CCGTGGATCTCAGCTTCGG

gDNA primers (ChIP-qPCR):

**Target**	**Forward**	**Reverse**
hACVRL1	AGAACGCTCTTCGCTAGGTG	CTGTGTGCTCACTGACTTGC

hAURKB	TTGGGACCCGTACTTTGGAG	GCGAGTTCCTGCTGGGAAAC
hBLM	ACCTAAACAGCATGTAAAGCCA	AACACCTGTCTTCTCCCCCT
hEGLN1	CTTAGGACTCGGAAGCGGC	GAGCGGGGAGAGAGATAGGG
hHK2	TGGCATCTCGGGATCATGTG	GGCCGACTCTTGTATTGCCT
hIER3	AGAGTGACACATGGTGAGCC	CGCCCAATTTTCAGGAGCAC
hNFE2L2	GTTGGCCCTTTCCTGCTTTA	GCGCAACAGATCAACAGCTC
hNUDT6	GTAATCCCCTGAGTGGAGCG	TGCCGTTACAGCTGTTTCAA
hPCNA	ACACCTACGAGCGCATCAAT	CTACCGGGAGGAAAAGCCTC

Antibodies:

HIF-1α - HIF-1α (D1S7W) XP® Rabbit mAb (Cell Signalling Technology)

IgG control - Normal Rabbit IgG #2729 (Cell Signalling Technology)

HIF-1β - HIF-1β/ARNT (D28F3) XP® Rabbit mAb #5537 (Cell Signalling Technology)

β-Actin - Anti-actin hFAB Rhodamine (Biorad)

β-Actin – β-Actin (13E5) Rabbit mAb #4970 (Cell Signalling Technology)

HDAC1 – HDAC1 (10E2) Mouse mAb #5356 (Cell Signalling Technology)

Histone H3 - Histone H3 Polyclonal antibody #17168-1-AP (Proteintech)

ERK1/2 - p44/42 MAPK (Erk1/2) (L34F12) mouse mAb #4696 (Cell Signalling Technology)

pERK1/2 - Phospho-p44/42 MAPK (Erk1) (Tyr204)/(Erk2) (Tyr187) (D1H6G) Mouse mAb #5726 (Cell Signalling Technology)

p70 S6K - p70 S6 kinase α Antibody (H-9): sc-8418 mouse mAb (Santa Cruz)

pp70 S6K (T389) - Phospho-p70 S6 Kinase (Thr389) (108D2) Rabbit mAb #9234 (Cell Signalling Technology)

PIM1 - PIM1 Recombinant Polyclonal Antibody (19HCLC) (Invitrogen)

pJAK2 (Y1007/8) - Phospho-Jak2 (Tyr1007/1008) (C80C3) Rabbit mAb #3776 (Cell Signalling Technology)

Additional antibodies used in multiplex fluorescent barcoding and phospho-flow cytometry (28).

Supplementary figure legends

Figure S1

(A) Mean peak intensity HIF-1α binding to chromatin in hMPL BaF3 JAK2 WT and JAK2V617F cells in normoxia and hypoxia (WT_Nx, WT_Hx, VF_Nx and VF_Hx respectively). **(B)** Hypoxia-induced occupancy-profiles of the indicated canonical HIF-1 target-gene promoters expressed as relative peak intensity normalised to normoxia from ChIP-seq data in JAK2V617F compared to JAK2 WT cells. **(C)** Binding intensity of key canonical HIF-1α binding targets. **(D)** Gene track of Aldoa genomic locus (x-axis) denoting strength of HIF-1α binding (y-axis) for each of the 4 conditions. **(E)** Top 30 genes ranked by binding intensity for each of the conditions WT_Hx (top heatmap) VF_Nx (middle) and VF-Hx (bottom), with the corresponding ranking for each gene in the other conditions. **(F-G)** Genomic feature binding analysis and HIF-1α hypoxia-responsive element (HRE) binding of ChIP-seq data shown as percentage of HIF-1α binding loci localised to the indicated genomic features. **(H)** Plot of Log_2 genome-wide gene accessibility (as derived from (13) Supplementary Figure 4 'Differential gene accessibility score in HSC and HSCMY clusters in untreated patients with myelofibrosis.') in primary JAK2V617F-mutation carrying patient cells (x-axis) plotted against log_2 genome-wide HIF-1α genomic loci binding in hypoxia-treated JAK2V617F hMPL BaF3 cells, as determined by ChIP-seq. **(I)** STRING analysis of cofactor roles, as identified by RIME LC-MS.

Figure S2

(A) Gene ontology (GO) analysis of canonical HIF-1α (Hallmark_Hypoxic) gene signature displayed as enrichment bubble plot with designated GO term (y-axis) plotted against enrichment (x-axis). GO terms plotted in descending order of statistical significance as designated by p-value (from 0.01 (blue) to 0.04 (yellow)) with 'bubble' size indicating number of genes associated with the indicated function. **(B)** List of genes from canonical HIF-1 metagene signature (29) identified in JAK2V617F normoxic (VF_Nx) (left) and JAK2V617F hypoxic (VF_Hx) (right) gene signatures with common genes between VF_Nx and VF_Hx signatures highlighted in blue.

Figure S3

(A) 172-MPN patient cohort Kaplan-Meier plot survival curves subdivided into populations with high (above cohort median) (blue) or low (below cohort median) (red) expression of indicated canonical HIF1-α target gene, with overall significance of survival difference between populations shown on graph (p-value). Remaining patients in each population shown in table below with number. **(B)** 172-MPN patient cohort gene expression enrichment of canonical HIF-1α (Hallmark_Hypoxic) gene signature plotted by MIPSS70 (Mutation-Enhanced International Prognostic Score System for transplantation eligible-aged patients with overt PMF) classification. **(C)** Gene expression enrichment of VF_Nx gene signature in a 172-MPN patient cohort, plotted by MIPSS70 and **(D)** Kaplan-Meier plot survival curve subdivided into populations with high (above cohort median) (blue) or low (below cohort median) (red) expression of canonical HIF-1α (Hallmark_Hypoxic) gene signature. **(E)** 172-MPN patient cohort gene expression enrichment of VF_Hx gene signature plotted by severity of patient anaemia.

Figure S4

(A) Workflow for the identification of specific genes associated with significant differential progression/survival and AML transformation in JAK2V617F-positive MPN patients (HIF MPN-BP signature). Disparate genes between the VF_Hx/VF_Nx signatures (n=158), and

the VF_Hx/WT_Hx signatures (n=144), representing those genes gained and lost respectively, as HIF-1 target genes in the VF_Hx context were first identified. To delineate genes that were acquired as HIF-1 targets as a result of chromatin alterations and not aberrant HIF-1 function, we next screened out those with altered accessibility as determined by scATACseq (12). The remaining genes were then analysed for differential expression in bulk and scRNAseq JAK2V617F vs JAK2 WT (13,14), and finally differentially expressed genes were analysed for correlation with progression/survival and AML transformation in the 172 JAK2V617F-positive MPN patient cohort. **(B)** Volcano plot of bulk RNAseq data (14) from mouse lineage negative, Sca-1 positive, c-kit positive (LSK) cells or **(C)** mouse megakaryocytic-erythroid progenitor (MEP) cells from JAK2 WT (n=3) and JAK2V617F homozygous (n=3) C57/BL6 mice. Transcripts from genes identified within the VF_Hx gene signature highlighted in blue with transcripts with significance of expression higher than -log10 adj p value of 1 labelled. **(D-I)** From left to right: UMAP scRNAseq plot of healthy donor (left) and JAK2V617F-positive MPN patient cells (right) with cells expressing elevated levels of the indicated gene highlighted in purple; 172-MPN patient cohort gene expression enrichment of the indicated gene plotted by MIPSS70 classification; 172-MPN patient cohort Kaplan-Meier plot survival curve subdivided into populations with high (above cohort median) (blue) or low (below cohort median) (red) expression of the indicated gene with overall significance of survival difference between populations shown on graph (p-value), for the indicated genes.

Figure S5

(A) Expression of individual genes whose differential expression is significantly correlated with spontaneous AML transformation (HIF MPN-BP signature) in cohort of 11-MPN patients who spontaneously transformed to AML, plotted by matched expression pre- (MPN) (red) and post-spontaneous AML transformation (sAML) (blue). Upregulated genes significantly associated with spontaneous AML transformation (top 5 graphs), downregulated genes significantly associated with spontaneous AML transformation (bottom 8 graphs). **(B)** 172-

MPN patient cohort gene expression of indicated canonical HIF1-α target gene plotted by whether the patient spontaneously developed AML secondary to MPN disease or not. **(C)** Expression of indicated canonical HIF1-α target gene in cohort of 11 JAK2V617F MPN patients who spontaneously transformed to blast-phase MPN, plotted by matched expression pre- (MPN) (red) and post- transformation (MPN-BP) (blue). **(D)** HIF MPN-BP signature enrichment in haematopoietic cells from the BoneMarrowMap scRNAseq reference atlas, plotted by differentiation stage of normal haematopoiesis. **(E)** HIF MPN-BP signature enrichment in BEAT-AMLv2 cohort composed of de novo and secondary AML patients, plotted by the patients AML origin (either de novo or secondary). **(F)** HIF MPN-BP signature enrichment in expanded 1058 patient AML 'meta-cohort' (comprising patients spanning the TCGA, BEAT-AMLv2, Leucegene and LUMC cohorts) plotted by ELN clinical risk class.

Figure S6

(A) Immunoblot of HIF-1α levels in JAK2 WT and JAK2V617F UT7/TPO cells starved overnight, followed by culturing in hypoxic (1% O_2) conditions and treatment as indicated with either Ruxolitinib, GN44028, Roxadustat or vehicle control for the indicated length of time. β-actin loading control (data shown representative of n=3). **(B)** Immunoblot of HIF-1α and HIF-1β levels in cytoplasmic, nuclear soluble and chromatin-bound lysate fractions from JAK2 WT and JAK2V617F UT7/TPO cells treated as indicated for 24hrs with either Ruxolitinib, Roxadustat, GN44028 or vehicle control. β-actin, HDAC1 and Histone H3 loading controls as indicated (data shown representative of n=3). **(C)** Heatmap of HIF-1α phosphorylation site signal intensity, as determined by phospho-mass spectrometry performed on hypoxic (1% O_2) treated JAK2V617F and WT JAK2 UT7/TPO cell lysate. **(D)** Gating strategy for multiplex fluorescent barcoding and phospho-flow cytometry. **(E)** Multiplex fluorescent phospho-flow cytometry heatmap of fold change of mean channel fluorescence intensity (MFI) (blue = low fold change, yellow = high fold change) by phosphorylated protein in JAK2 WT and JAK2V617F UT-7/TPO cells treated with TPO or

without. Data normalised based on fold change to mean value of WT starved, no TPO, normoxic condition (n=3). **(F)** Immunoblot of the indicated proteins in JAK2 WT UT7/TPO cells, treated as indicated with either TCS PIM1.1, SMI-4a, PIM447 or vehicle control. β-actin loading control (data shown representative of n=3). **(G)** Immunoblot of the indicated proteins in JAK2 WT hMPL BaF3 cells, treated as indicated with either TCS PIM1.1, SMI-4a, PIM447 or vehicle control. β-actin loading control (data shown representative of n=3).

Figure S7

(A) Barcoded phospho-flow cytometry heat map of the indicated protein levels in JAK2V617F and WT JAK2 UT7/TPO cells, cultured in normoxic (20% O2) or hypoxic (1% O2) conditions, starved overnight or treated with TPO and SMI-4a, Ruxolitinib or vehicle control as indicated. Heatmap of mean channel fluorescence intensity (MFI), data normalised based on fold change to mean value of WT starved, no TPO, normoxic condition (n=3). **(B) I**mmunoblot of the indicated proteins in SET2 and HEL cells treated as indicated for 6hrs with either Ruxolitinib, SMI-4a or vehicle control. Immunoblotting carried out with β-actin as a loading control (data shown representative of n=3). **(C)** Percentage of cell population undergoing the indicated stage of cell death as determined by Annexin V Apoptosis assay in JAK2 WT and JAK2V617F UT7/TPO cells treated as indicated with Roxadustat, GN44028 or DMSO control for 72hrs in either normoxic (20% O_2) or hypoxic (1% O_2) conditions. **(D)** Fold enrichment of the indicated genes from HIF-1α ChIP-isolated gDNA by qPCR. ChIP performed on lysate from JAK2V617F UT7/TPO cells starved overnight and then treated as indicated with Roxadustat, Ruxolitinib, GN44028 or DMSO control for 24hrs in hypoxic (1% O2) conditions. **(E)** Gene expression of HIF1-MPN-BP signature genes in UT7/TPO WT and JAK2V617F cells starved overnight and then treated as indicated with GN44028, Roxadustat or DMSO control for 24hrs in hypoxic (1% O2) conditions (n=3).

Supplementary References

1. Andrews S. FastQC: a quality control tool for high throughput sequence data. 2010;
2. Bolger AM, Lohse M, Usadel B. Trimmomatic: a flexible trimmer for Illumina sequence data. Bioinformatics. 2014 Aug 1;30(15):2114–20.
3. Dobin A, Davis CA, Schlesinger F, Drenkow J, Zaleski C, Jha S, et al. STAR: ultrafast universal RNA-seq aligner. Bioinformatics. 2013 Jan 1;29(1):15–21.
4. Danecek P, Bonfield JK, Liddle J, Marshall J, Ohan V, Pollard MO, et al. Twelve years of SAMtools and BCFtools. Gigascience. 2021 Feb 16;10(2):giab008.
5. Amemiya HM, Kundaje A, Boyle AP. The ENCODE blacklist: Identification of problematic regions of the genome. Sci Rep. 2019 Jun 27;9(1):9354.
6. Quinlan AR, Hall IM. BEDTools: a flexible suite of utilities for comparing genomic features. Bioinformatics. 2010 Mar 15;26(6):841–2.
7. Zhang Y, Liu T, Meyer CA, Eeckhoute J, Johnson DS, Bernstein BE, et al. Model-based analysis of ChIP-Seq (MACS). Genome Biol. 2008 Sep 17;9(9):R137.
8. Ross-Innes CS, Stark R, Teschendorff AE, Holmes KA, Ali HR, Dunning MJ, et al. Differential oestrogen receptor binding is associated with clinical outcome in breast cancer. Nature. 2012 Jan 4;481(7381):389–93.
9. Gel B, Serra E. karyoploteR: an R/Bioconductor package to plot customizable genomes displaying arbitrary data. Bioinformatics. 2017 Oct 1;33(19):3088–90.
10. Marçais G, Kingsford C. A fast, lock-free approach for efficient parallel counting of occurrences of k-mers. Bioinformatics. 2011 Mar 15;27(6):764–70.
11. Medeiros JJF, Zeng AGX, Chan-Seng-Yue M, Woo T, Bansal S, Kim H, et al. Stem cell-derived gene expression scores predict survival and blastic transformation in myelofibrosis [Internet]. bioRxiv. 2024. Available from: https://www.medrxiv.org/content/10.1101/2024.07.09.24310101v1.abstract
12. Izzo F, Myers RM, Ganesan S, Mekerishvili L, Kottapalli S, Prieto T, et al. Mapping genotypes to chromatin accessibility profiles in single cells. Nature. 2024 May;629(8014):1149–57.
13. Psaila B, Wang G, Rodriguez-Meira A, Li R, Heuston EF, Murphy L, et al. Single-Cell Analyses Reveal Megakaryocyte-Biased Hematopoiesis in Myelofibrosis and Identify Mutant Clone-Specific Targets. Mol Cell. 2020 May 7;78(3):477-492.e8.
14. Li B, An W, Wang H, Baslan T, Mowla S, Krishnan A, et al. BMP2/SMAD pathway activation in JAK2/p53-mutant megakaryocyte/erythroid progenitors promotes leukemic transformation. Blood. 2022 Jun 23;139(25):3630–46.
15. Zeng AGX, Iacobucci I, Shah S, Mitchell A, Wong G, Bansal S, et al. Single-cell transcriptional atlas of human hematopoiesis reveals genetic and hierarchy-based determinants of aberrant AML differentiation. Blood Cancer Discov. 2025 Apr 28;OF1–18.
16. Severens JF, Karakaslar EO, van der Reijden BA, Sánchez-López E, van den Berg RR, Halkes CJM, et al. Mapping AML heterogeneity - multi-cohort transcriptomic analysis identifies novel clusters and divergent ex-vivo drug responses. Leukemia. 2024 Apr 15;38(4):751–61.
17. Döhner H, Wei AH, Appelbaum FR, Craddock C, DiNardo CD, Dombret H, et al. Diagnosis and management of AML in adults: 2022 recommendations from an international expert panel on behalf of the ELN. Blood. 2022 Sep 22;140(12):1345–77.

18. Bottomly D, Long N, Schultz AR, Kurtz SE, Tognon CE, Johnson K, et al. Integrative analysis of drug response and clinical outcome in acute myeloid leukemia. Cancer Cell. 2022 Aug 8;40(8):850-864.e9.
19. Ma X, Liu Y, Liu Y, Alexandrov LB, Edmonson MN, Gawad C, et al. Pan-cancer genome and transcriptome analyses of 1,699 paediatric leukaemias and solid tumours. Nature. 2018 Mar 15;555(7696):371–6.
20. Verhaak RGW, Wouters BJ, Erpelinck CAJ, Abbas S, Beverloo HB, Lugthart S, et al. Prediction of molecular subtypes in acute myeloid leukemia based on gene expression profiling. Haematologica. 2009 Jan 1;94(1):131–4.
21. Martin M. Cutadapt removes adapter sequences from high-throughput sequencing reads. EMBnet J. 2011 May 2;17(1):10.
22. Patro R, Duggal G, Love MI, Irizarry RA, Kingsford C. Salmon provides fast and bias-aware quantification of transcript expression. Nat Methods. 2017 Apr 6;14(4):417–9.
23. Frankish A, Diekhans M, Ferreira A-M, Johnson R, Jungreis I, Loveland J, et al. GENCODE reference annotation for the human and mouse genomes. Nucleic Acids Res. 2019 Jan 8;47(D1):D766–73.
24. Pimentel H, Bray NL, Puente S, Melsted P, Pachter L. Differential analysis of RNA-seq incorporating quantification uncertainty. Nat Methods. 2017 Jul 5;14(7):687–90.
25. Veidenberg A, Medlar A, Löytynoja A. Wasabi: An integrated platform for evolutionary sequence analysis and data visualization. Mol Biol Evol. 2016 Apr;33(4):1126–30.
26. Hao Y, Stuart T, Kowalski MH, Choudhary S, Hoffman P, Hartman A, et al. Dictionary learning for integrative, multimodal and scalable single-cell analysis. Nat Biotechnol. 2024 Feb;42(2):293–304.
27. Andreatta M, Carmona SJ. UCell: Robust and scalable single-cell gene signature scoring. Comput Struct Biotechnol J. 2021 Jun 30;19:3796–8.
28. Tsutsumi N, Masoumi Z, James SC, Tucker JA, Winkelmann H, Grey W, et al. Structure of the thrombopoietin-MPL receptor complex is a blueprint for biasing hematopoiesis. Cell. 2023 Sep 14;186(19):4189-4203.e22.
29. Lombardi O, Li R, Halim S, Choudhry H, Ratcliffe PJ, Mole DR. Pan-cancer analysis of tissue and single-cell HIF-pathway activation using a conserved gene signature. Cell Rep. 2022 Nov 15;41(7):111652.

Figure S1

Keealy et al.

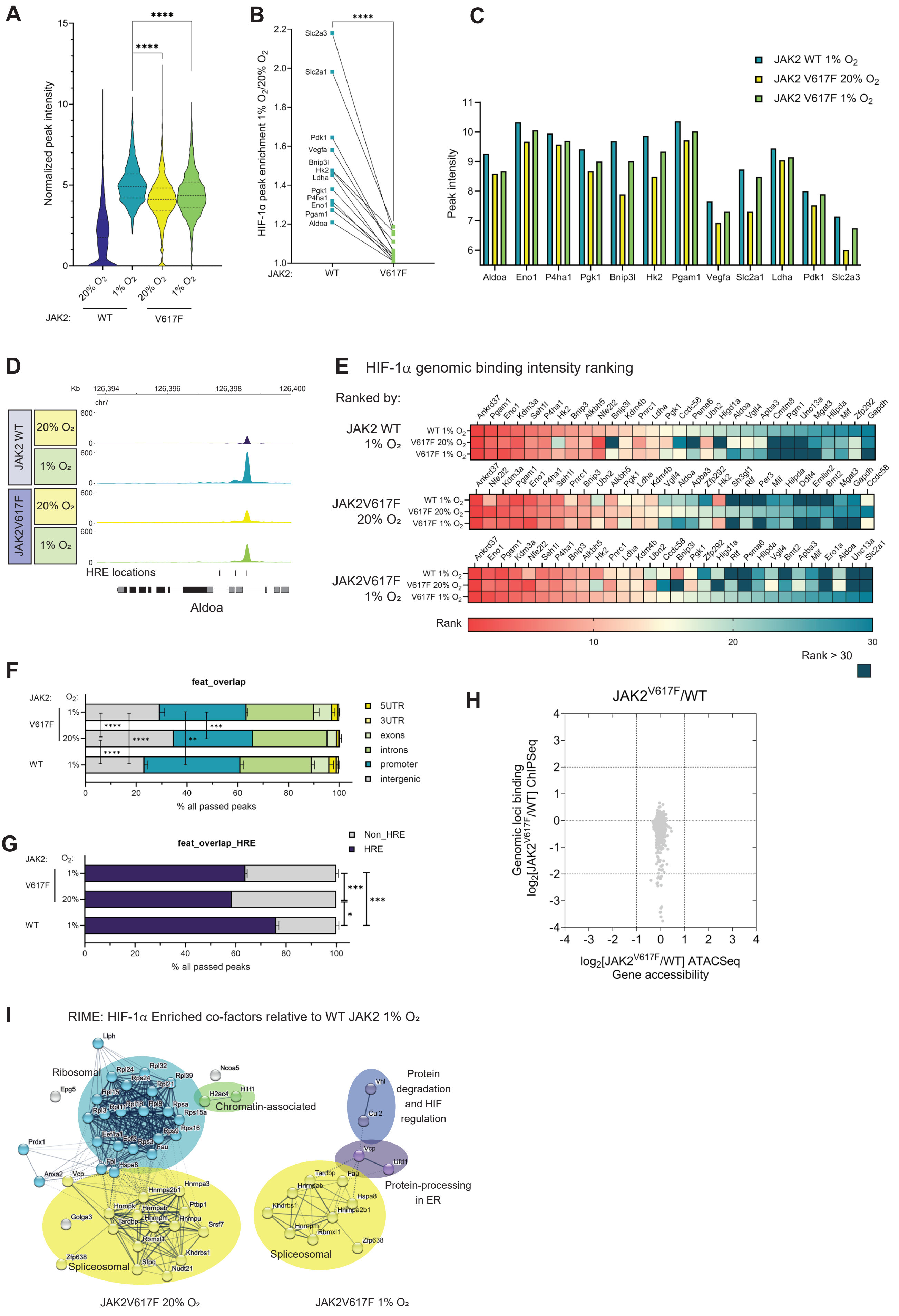

Figure S2

Kealy et al.

A

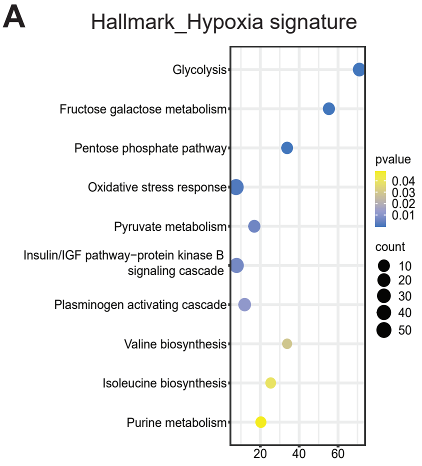

B

Signature:	VF_Nx	VF_Hx
HIF-1 Metagene	RBPJ	RBPJ
	ENO1	DDIT4
	GBE1	
	KDM4B	
	LDHA	
	MXI1	
	PGAM1	
	PGK1	
	AK4	

Figure S3

Kealy et al.

A 172 JAK2V617F MPN patient cohort : survival

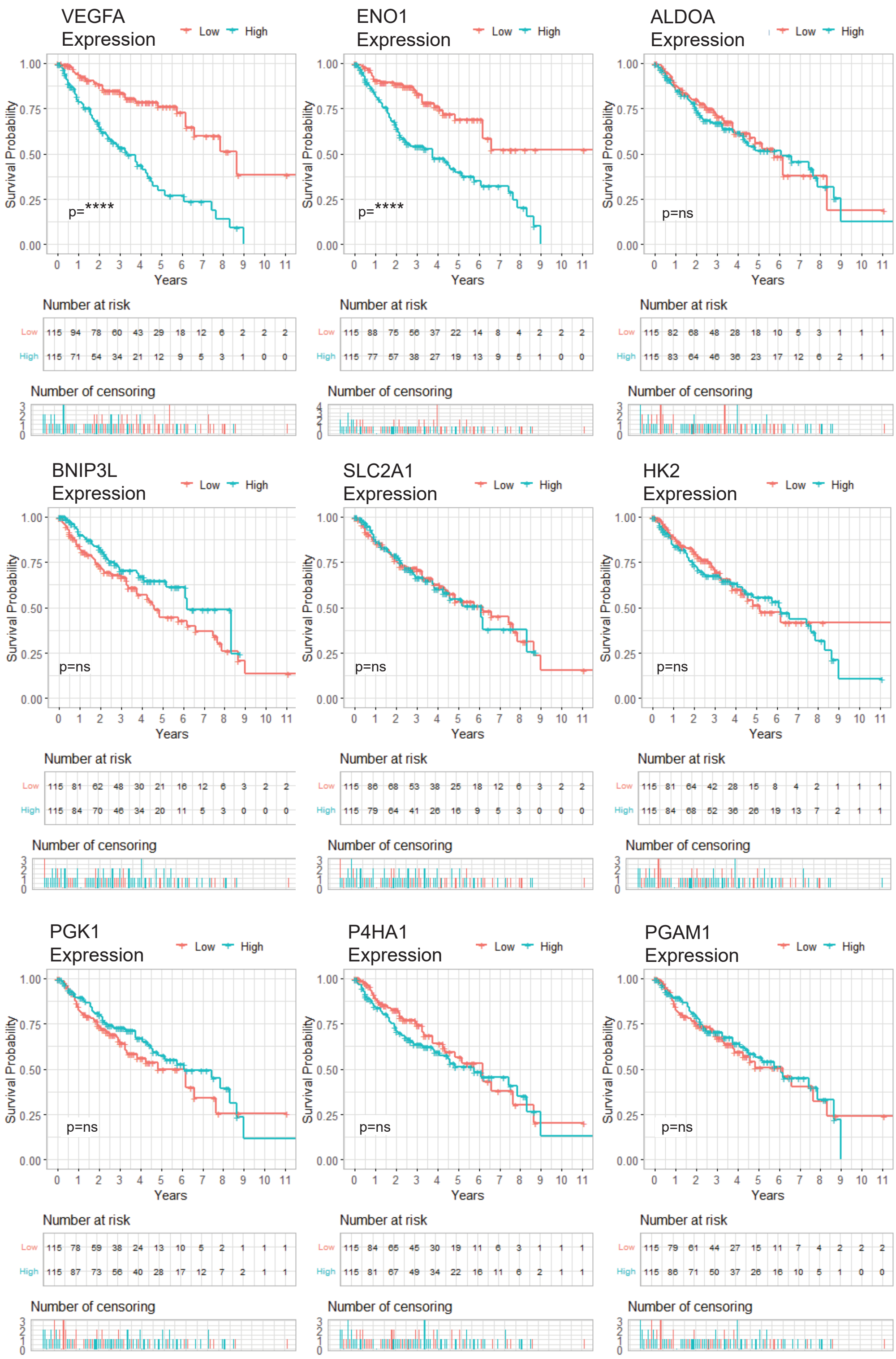

Figure S3

Kealy et al.

B 172 JAK2V617F MPN patient cohort: disease severity (MIPSS70 classification)

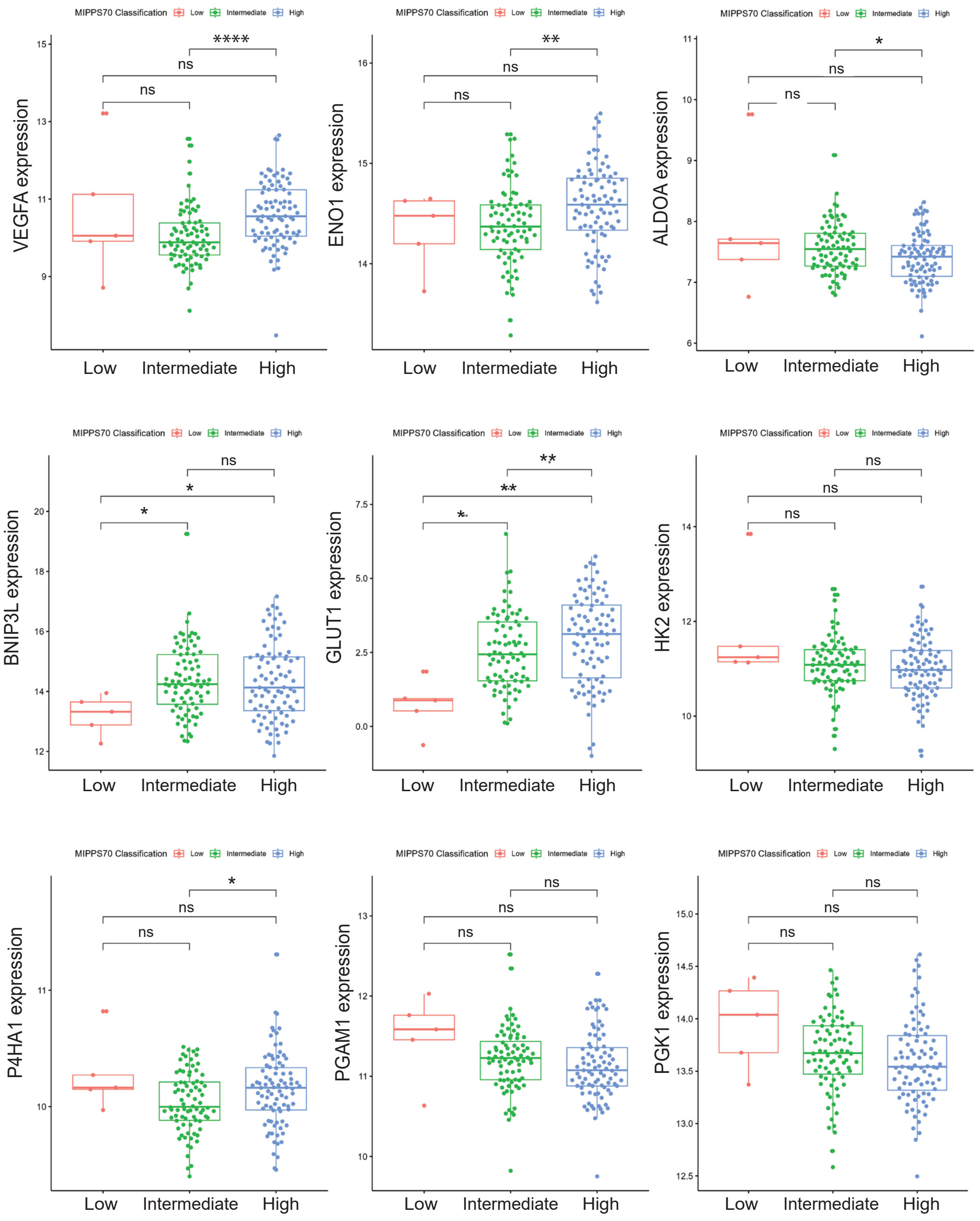

C

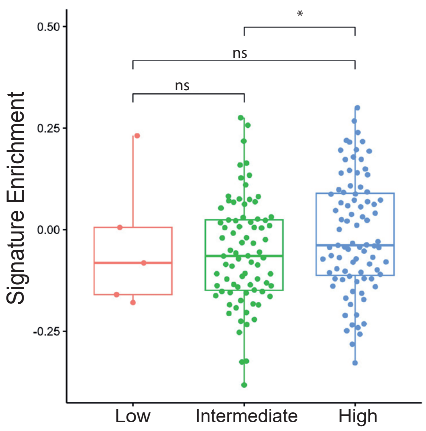

D

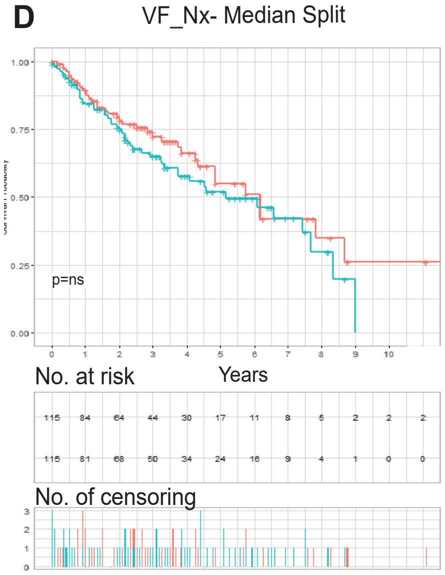

E JAK2V617F-HIF-1 signature expression in MPN patients correlation with anemia.

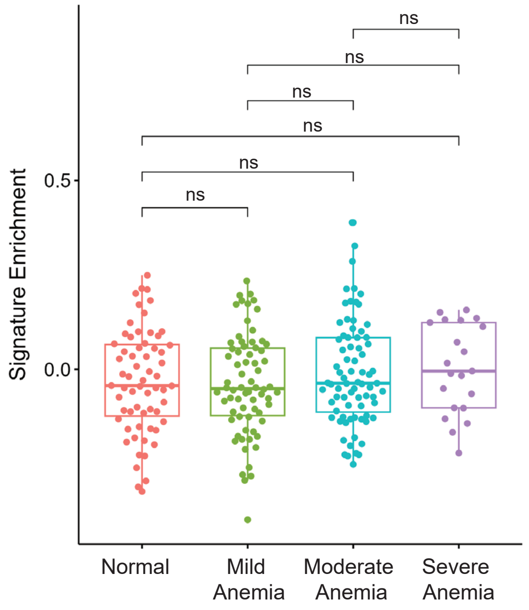

Figure S4

Kealy et al.

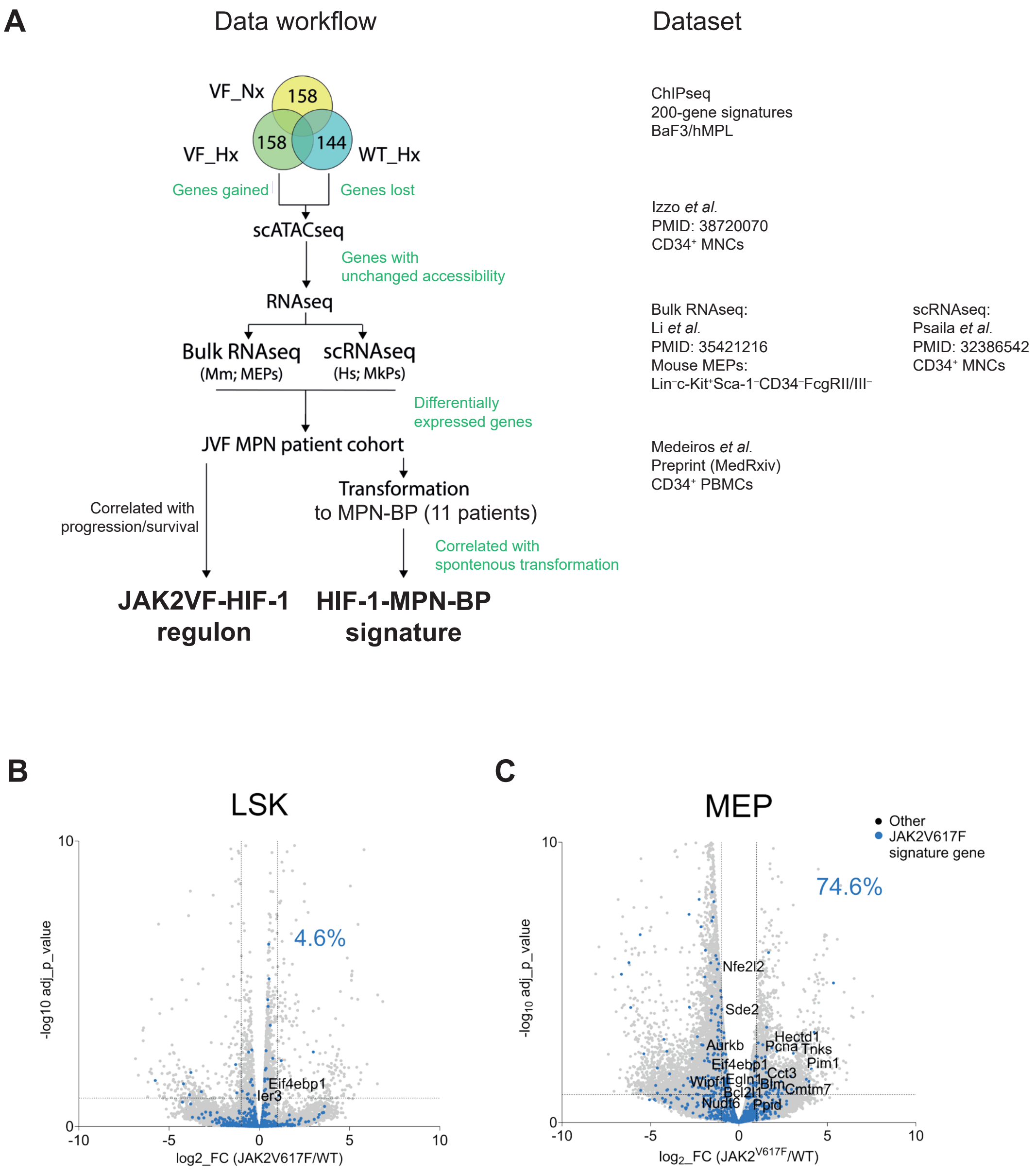

Figure S4

Kealy et al.

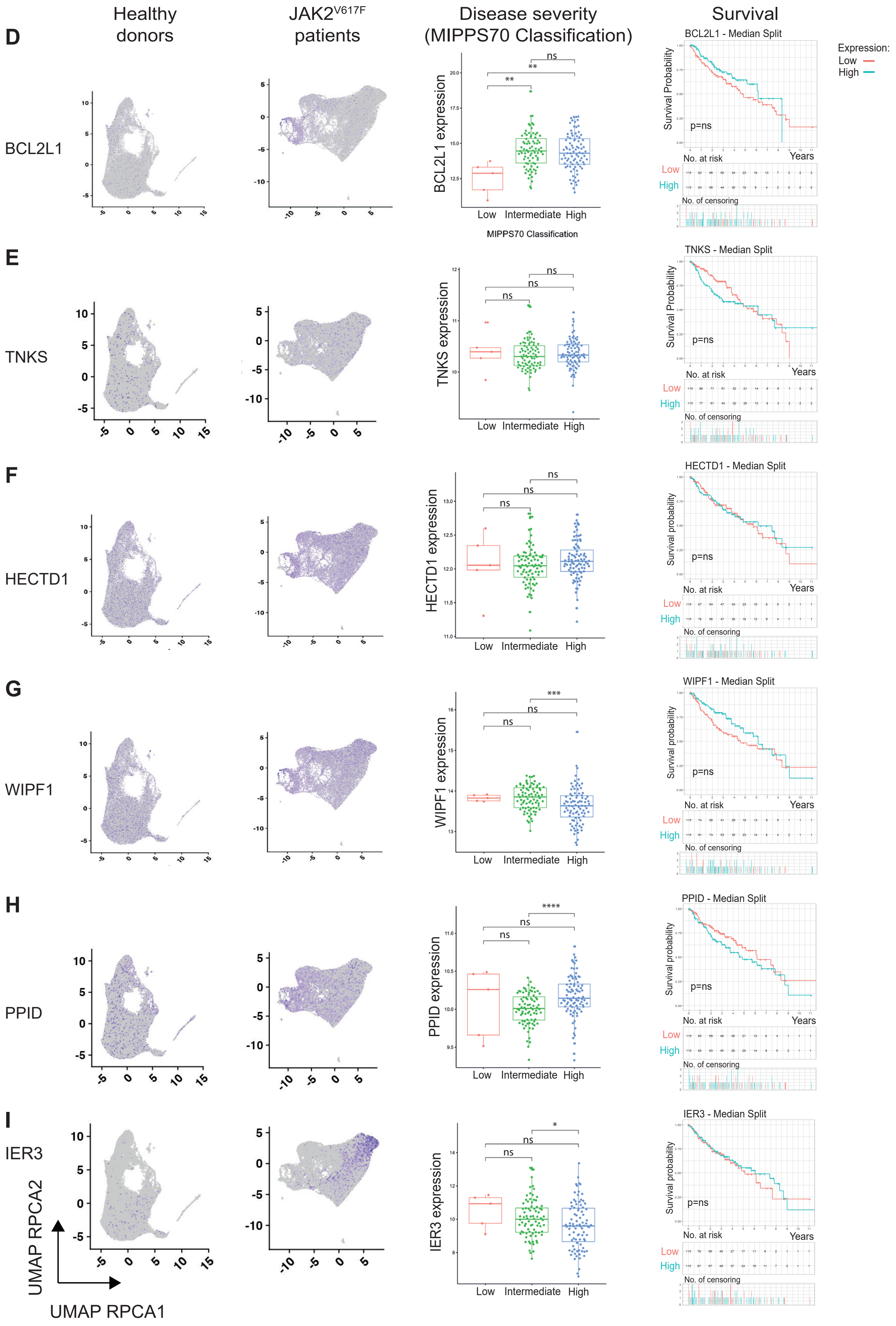

Figure S5

Kealy et al.

A ***HIF MPN-BP signature:***

JAK2V617F-HIF-1 gene expression in MPN patients correlated with spontaneous transformation to AML.

Upregulated genes

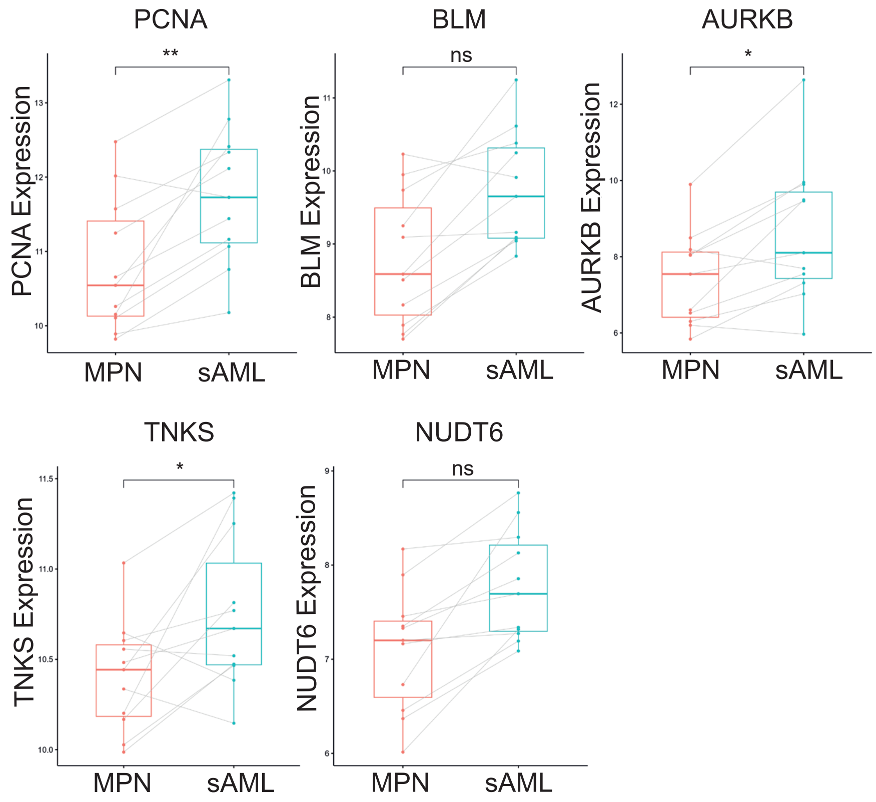

Downregulated genes

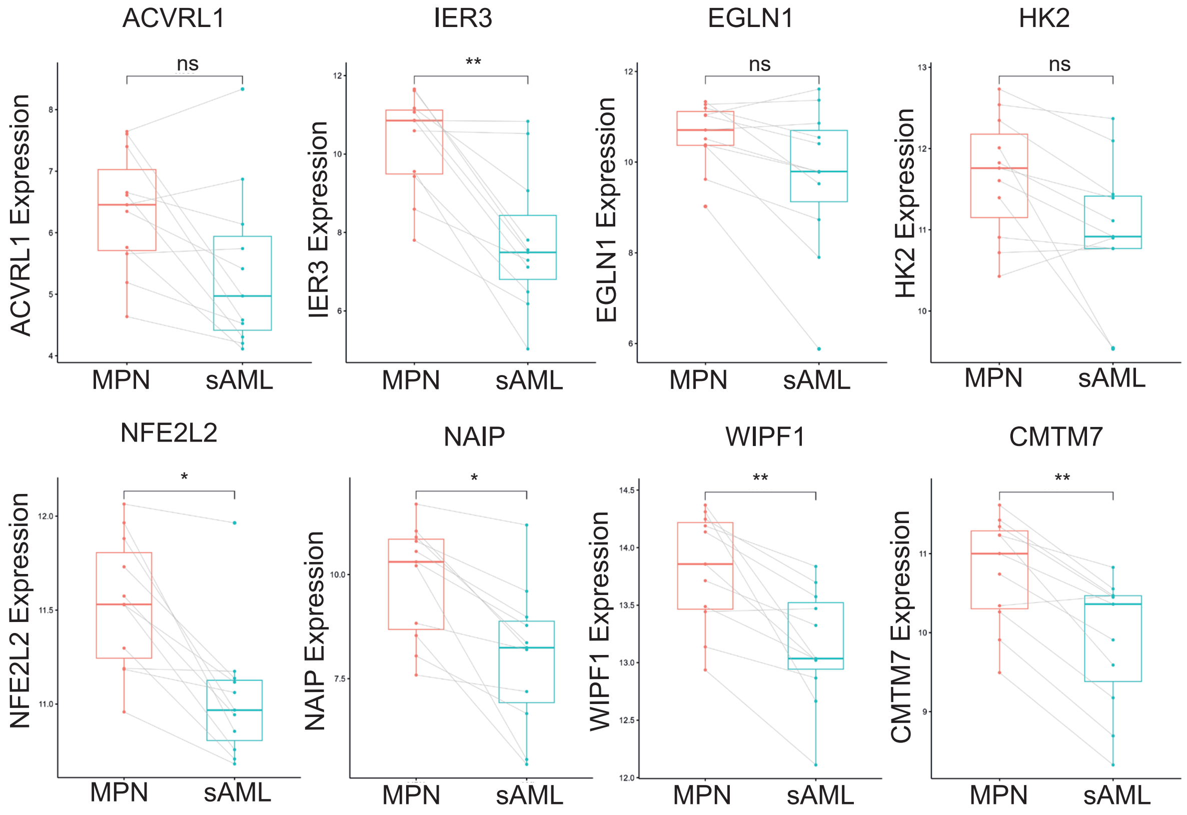

B Transformation to MPN blast-phase (MPN-BP)

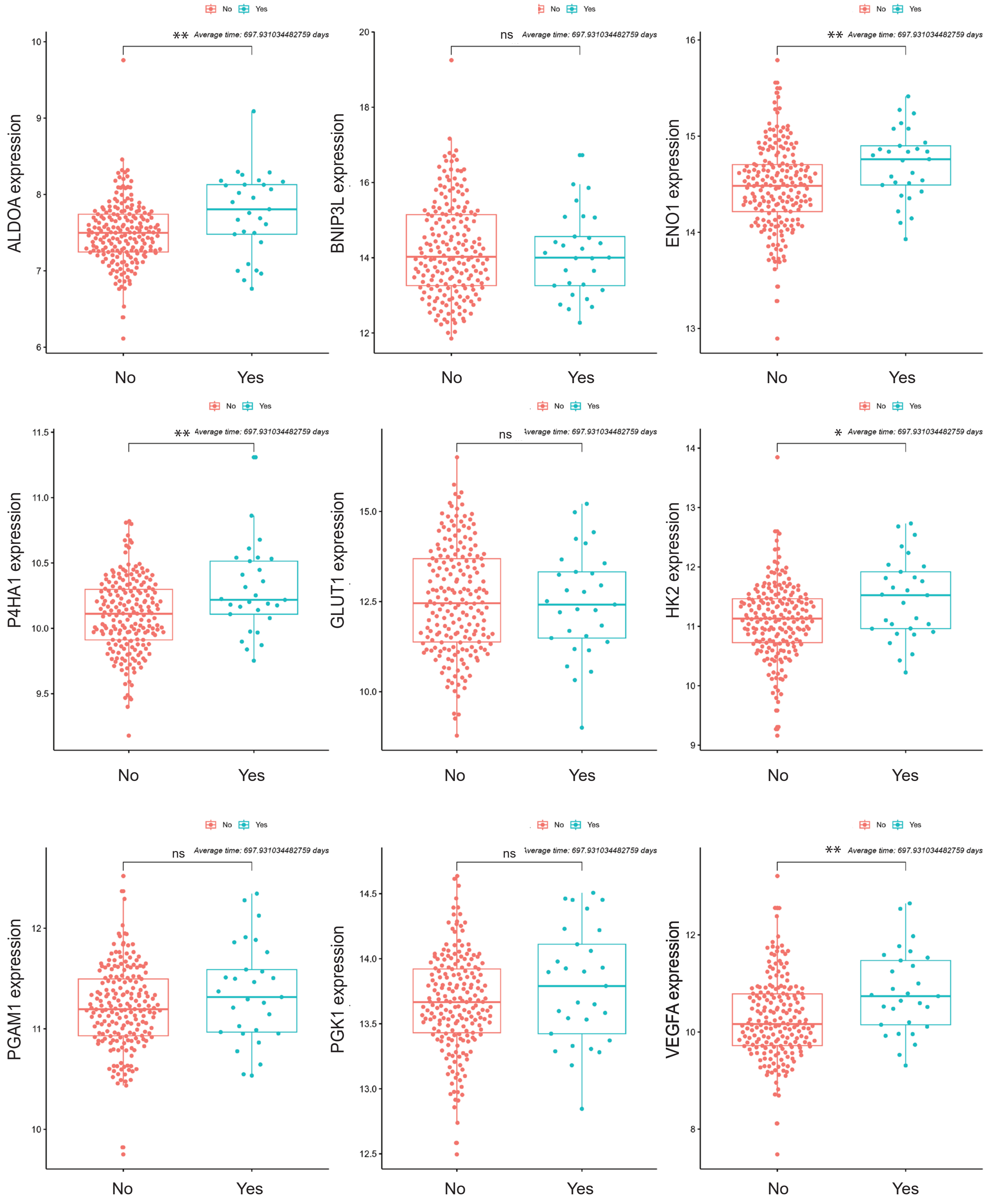

C Pre- and post-transformation to MPN-BP

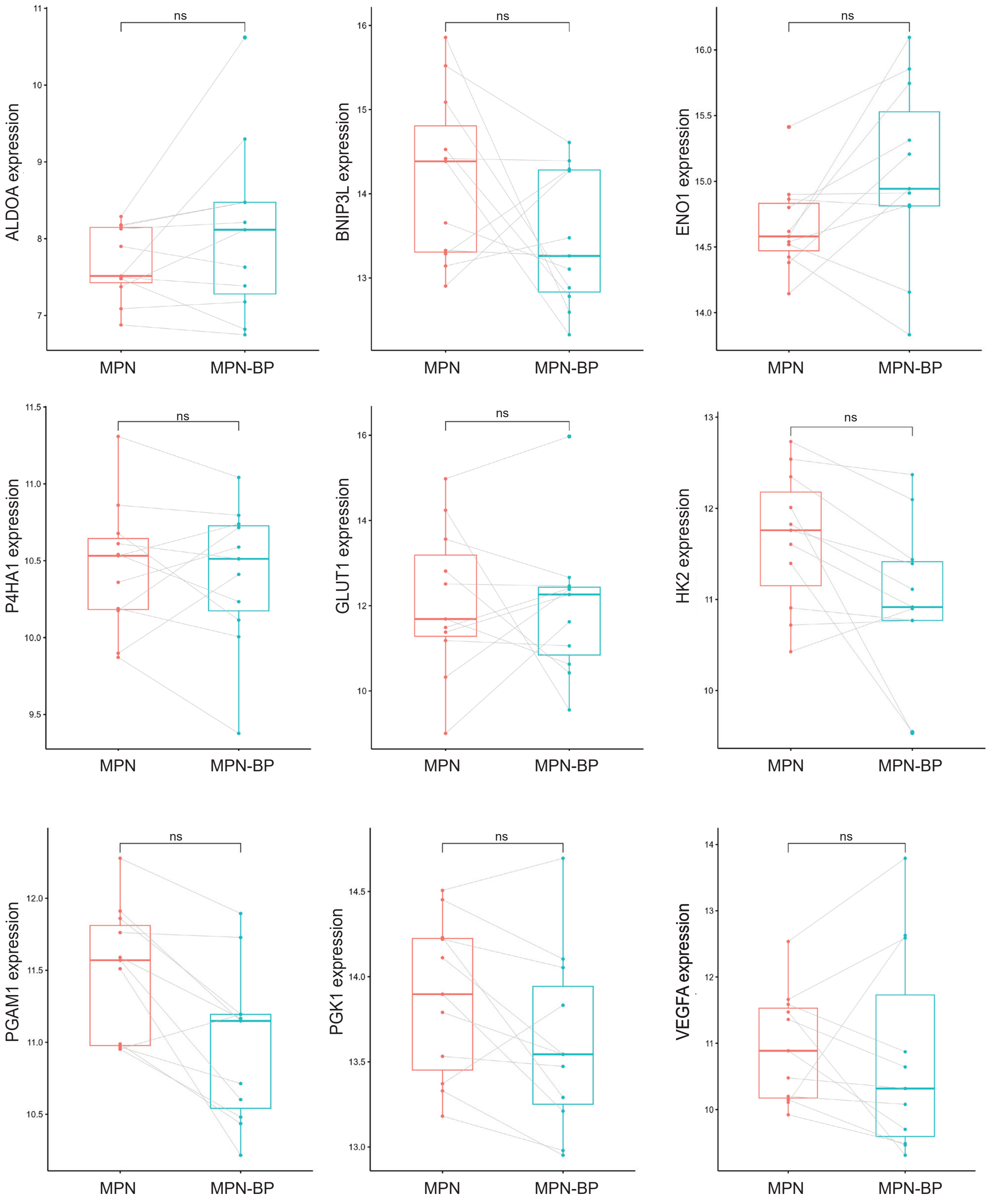

Figure S5

Kealy et al.

D

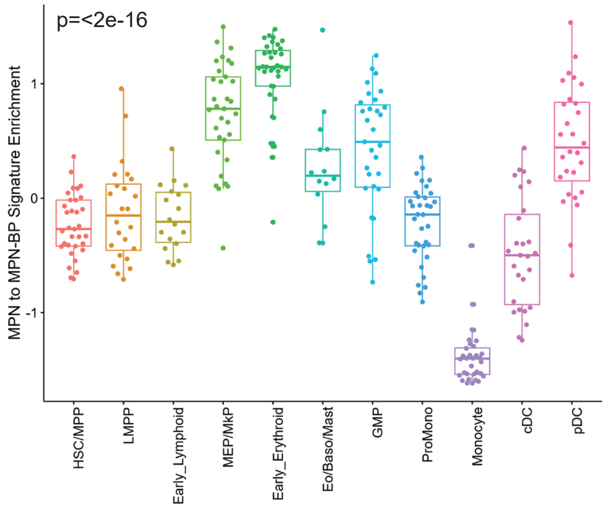

E

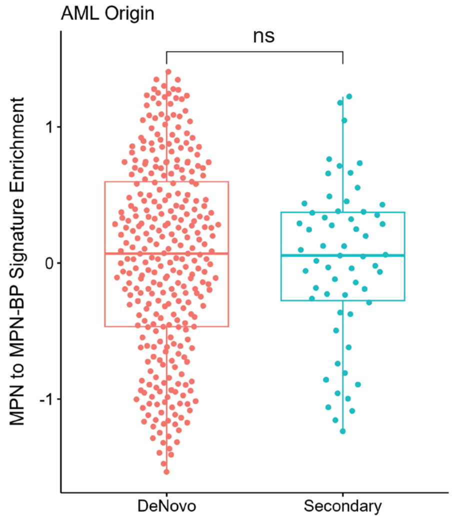

F

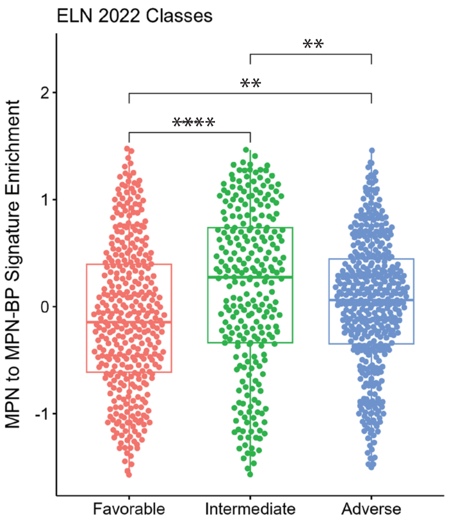

Figure S6

Kealy et al.

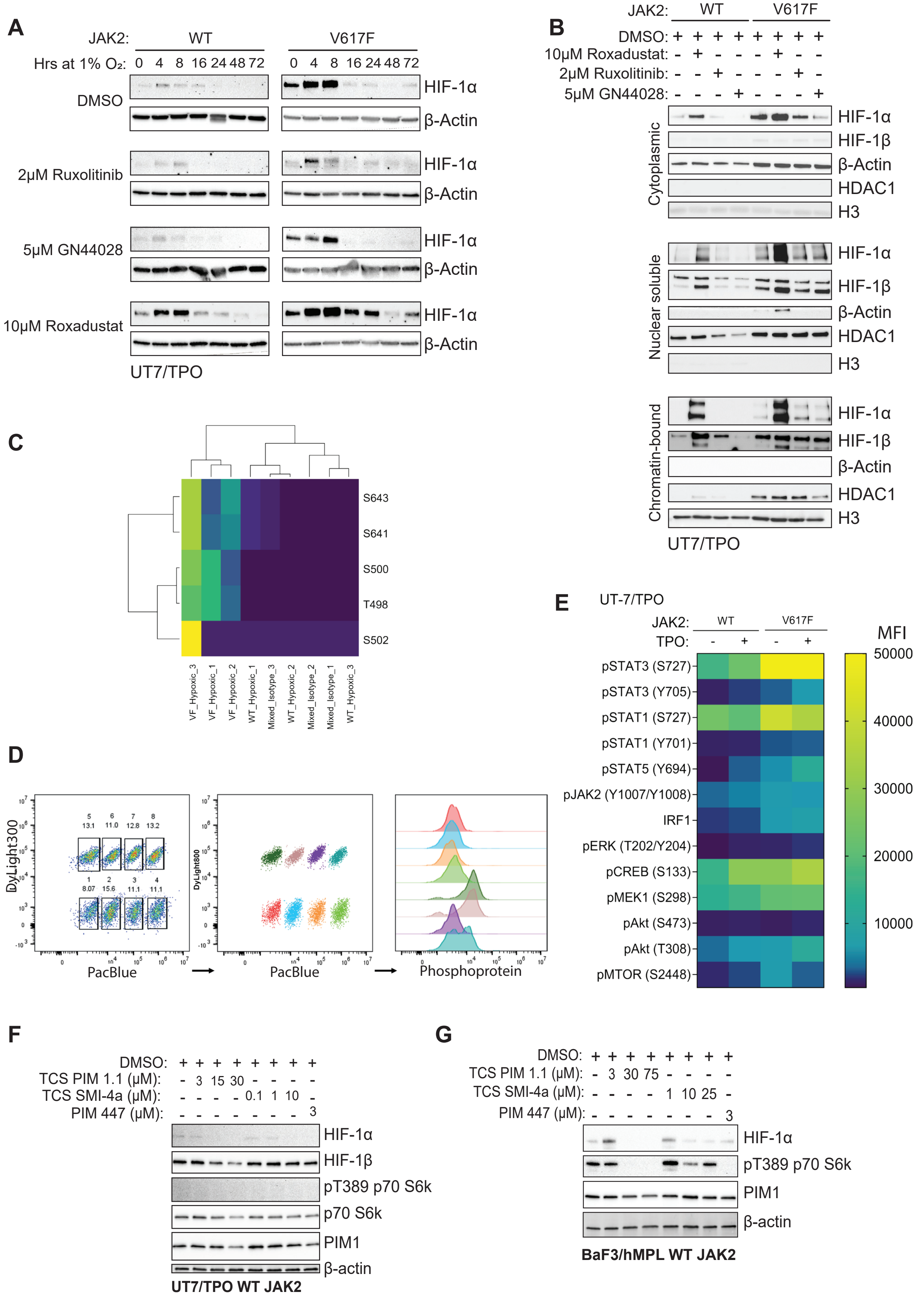

Figure S7

Kealy et al.

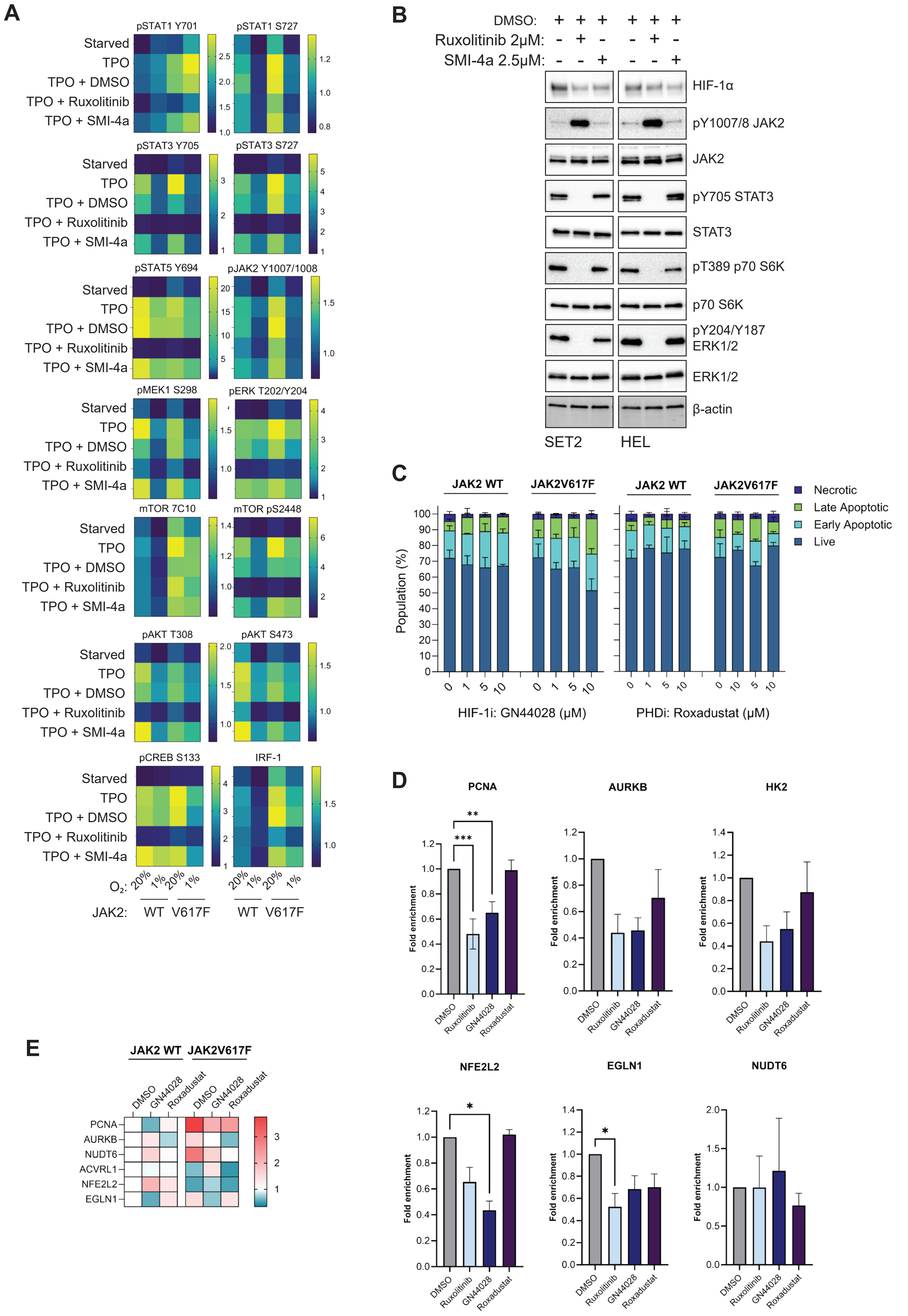

Supplement: Supplementary file 5 — Supplementary Information [file 41375_2025_2843_MOESM5_ESM.pdf]
